# Blue Light Controlled Supramolecular Soft Robotics of Phenylazothiazole Amphiphiles for Rapid Macroscopic Actuations

**DOI:** 10.1002/advs.202407130

**Published:** 2024-10-16

**Authors:** Ming‐Hin Chau, Wai‐Ki Wong, Takashi Kajitani, Franco King‐Chi Leung

**Affiliations:** ^1^ State Key Laboratory of Chemical Biology and Drug Discover Research Institute for Future Food Department of Applied Biology and Chemical Technology The Hong Kong Polytechnic University Hong Kong China; ^2^ Centre for Eye and Vision Research 17 W Hong Kong Science Park Hong Kong China; ^3^ Open facility Development Office Open Facility Center Tokyo Institute of Technology 4259 Nagatsuta, Midori‐ku Yokohama 226‐8501 Japan

**Keywords:** biocompatible soft scaffold, blue‐light driven actuation, phenylazothiazole amphiphiles, soft robotics, supramolecular chemistry

## Abstract

Nature preprograms sophisticated processes in operating molecular machines at the nanoscale, amplifying the molecular motion across multiple length‐scales, and controlling movements in living organisms. Supramolecular soft robotics serve as a new alternative to hard robotics, are able to transform and amplify collective motions of the supramolecularly assembled molecular machines in attaining macroscopic motions, upon photoirradiation. By taking advantage of oriented supramolecular macroscopic soft scaffold, here the first rapid macroscopic movements of supramolecular robotic materials driven by visible light are presented. Head‐tail amphiphilic structure is designed with the phenylazothiazole motif as the photoswitching core. Unidirectionally aligned nanostructures of the amphiphilic phenylazothiazoles are controlled by non‐invasive blue light irradiation and bends toward the light source, demonstrating a fast macroscopic actuation of supramolecular robotic systems (up to 17° s^−1^) in aqueous media. Through meticulous X‐ray diffraction and electron microscopy analyzes, macroscopic actuation mechanism is illustrated in a tight relation to molecular geometric transformations upon photoisomerization. By elucidating the key macroscopic actuation parameters, this paves the way for the next generation design of supramolecular soft robotic systems with enhanced biomimetic actuating functions.

## Graphical abstract

This study presents the macroscopic soft scaffold of supramolecularly assembled phenylazothiazoles amphiphile, prepared by shear‐flow method can be controlled by non‐invasive blue light irradiation and bends toward the light source, demonstrating a rapid macroscopic actuation of supramolecular robotic system (up to 17 ° s^−1^) in aqueous media.

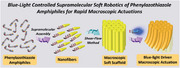

## 1 Introduction

Movement is one of the vital functions in living organisms for survival and reproduction.^[^ ^1^, ^2^, ^3^ ^]^ Sophisticated biochemical processes for signal transduction and energy conversion from chemical energy to mechanical motions are synchronized to control movements.^[^ ^4^ ^]^ Natural protein motors, such as myosin of skeletal muscle, drive efficiently for movements through amplifying collective molecular motions from nanoscale to macroscopic length‐scale.^[^ ^1^ ^]^ Movements found in nature have inspired scientists to develop artificial robotic systems. Rapid advancements in industrial manufacturing processes and materials science have enabled numerous state‐of‐the‐art technological developments.^[^ ^5^ ^]^ Namely, conventional hard robotics, which is produced from rigid structural materials, convert energy to mechanical motions for mimicking animal‐like functions, e.g., expansion, contraction, and stiffness change.^[^ ^6^ ^]^ Soft robotics has been considered as the complementary counterpart to hard robotics, though the technological development of soft robotics is still in its infancy.^[^ ^7^, ^8^ ^]^ The next generations of biocompatible and safe actuating robotic systems provide an interface between living systems and artificial systems at multiple levels.^[^ ^9^, ^10^, ^11^ ^]^ A variety of soft matters, e.g., polymeric elastomers,^[^ ^12^, ^13^, ^14^ ^]^ polymer gel,^[^ ^15^, ^16^, ^17^ ^]^ and hydrogels,^[^ ^18^, ^19^, ^20^, ^21^, ^22^, ^23^, ^24^ ^]^ have been designed into soft robotics, driven by different external stimulations. Taking inspiration from molecular motions in natural muscle tissue, an alternative fabrication method of soft actuating robotics is functionalizing molecular machines through organic synthetic strategies to create covalently bonded polymers or supramolecular systems.^[^ ^25^, ^26^, ^27^, ^28^, ^29^ ^]^ Recent developments of hierarchical supramolecular assemblies of small synthetic molecules, biomolecules, and macromolecules have enabled functional applications of the assemblies in biomedical, photoconductive, and stimuli‐responsive materials.^[^ ^27^, ^30^, ^31^, ^32^, ^33^, ^34^, ^35^, ^36^, ^37^, ^38^, ^39^, ^40^, ^41^, ^42^, ^43^ ^]^ The Supramolecular robotic system has emerged as the most promising alternative to all existing polymeric soft robotic systems, in considering its intrinsic supramolecular dynamicity, stimuli‐responsiveness, and bioactive material surface. Weak intermolecular interactions of 3D supramolecular robotic systems enable dynamic assembling and disassembling processes over time, as the fourth dimension. Molecular machinery embedded in highly dynamic and low Young‐modulus supramolecular structures inherit stimuli‐responsiveness, e.g., pH, light, solvent, ion, heat,^[^ ^44^, ^45^, ^46^, ^47^, ^48^, ^49^ ^]^ with significant 3D structural transformations. Bioactive functional surfaces of supramolecular robotic systems serve as cell‐material interfaces to connect and signal cells. These unprecedented functional properties of supramolecular robotic systems substantiate their key roles in serving as the next generation of soft robotic materials.

Molecular muscles and some microscopic motions were revealed with supramolecular nanostructures in organic media.^[^ ^50^, ^51^, ^52^, ^53^, ^54^, ^55^ ^]^ An azobenzene‐based system constructed macroscopic organogel supramolecularly assembled and released the entrapped organic solvents responsively upon photoisomerization.^[^ ^56^ ^]^ The first supramolecular robotic materials in aqueous media, reported by Feringa, was demonstrated with amplifying molecular motions of motor amphiphiles in pre‐organized supramolecular structure across multiple length‐scale to sustain macroscopic actuation upon UV‐light irradiation.^[^ ^57^, ^58^, ^59^ ^]^ The intrinsic nanostructure orientation was revealed as a key parameter to control macroscopic actuation. The construction of photoactuating extracellular matrix mimetic scaffold by supramolecular assemblies of molecular motor amphiphiles was further reported by Leung and Feringa.^[^ ^60^ ^]^ However, only the photoisomerization between stable‐isomer and unstable‐isomer of the molecular motor was designed to sustain macroscopic actuation processes, instead of harvesting all energy generated from the multiple‐states unidirectional 360° rotations of molecular motors. We envision that a two‐state molecular machine can sustain macroscopic actuation without complicated thermal (dark) processes. Besides, all reported motor amphiphiles‐based supramolecular robotic systems were driven by biodamaging high‐energy UV light. Soft robotic systems driven by NIR light were generally associated with a photothermal effect, the generated heat energy may alter the cellular environment and limit the functional versatility as a biocompatible scaffold. In this connection, a two‐state molecular machine‐based supramolecular soft robotic system, with simple and effective organic synthesis, controlled by visible light is urgently needed for developing the next generations of bioactive soft robotic materials.

**Figure 1:**
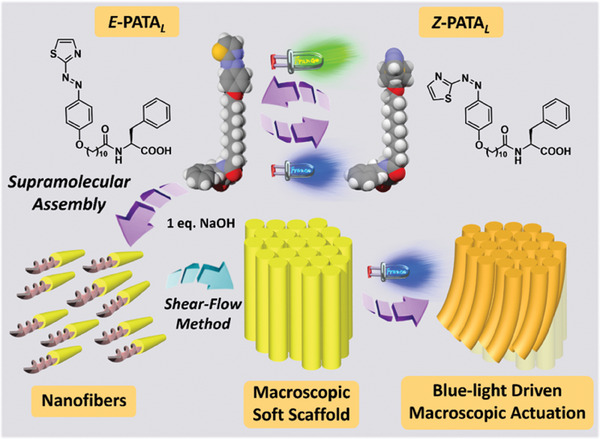
The molecular structures of PATA and the blue light‐driven macroscopic actuation process of their supramolecularly assembled structures in the macroscopic soft scaffold.

Molecular machines driven by visible light have been exemplified and shown good photoswitchabilities in aqueous media, such as indigo^[^ ^61^, ^62^ ^]^ and donor–acceptor Stenhouse adducts.^[^ ^63^ ^]^ One of the simplest molecular machines, azobenzene, and heteroaryl‐azobenzene motifs have been applied for smart functional materials and photochromic materials.^[^ ^64^, ^65^, ^66^, ^67^ ^]^ Different heteroaryl‐azobenzenes have been exemplified with improved *Z*/*E*‐ratio at the photostationary state.^[^ ^68^, ^69^, ^70^, ^71^ ^]^ Phenylazothiazole core, featured with blue and green light‐driven photoswitching, offered excellent photoswitchabilities in organic and aqueous media (**Figure** **1**). We present a phenylazothiazole amphiphile (PATA) with excellent photoswitchability in aqueous media and cyclability with limited signs of fatigue. Head‐tail amphiphilic structure of PATA enables the obtained supramolecular assemblies with a large aspect ratio. Significant supramolecular transformations of assembled structures of PATA can be driven by sequential blue and green light irradiation. The nanostructures of PATA can be stabilized by charge screening and formed as macroscopic soft scaffolds. Upon visible‐light irradiation, the macroscopic soft scaffold of PATA is bent toward the light source, sustaining a faster macroscopic actuation than other reported supramolecular soft robotics.^[^ ^59^, ^60^ ^]^ Through meticulous mechanistic investigations, the actuation mechanism of the PATA macroscopic soft scaffolds was revealed, along with remarkable robotic movement demonstrations, establishing a new generation of supramolecular robotic systems. The current approach could open up new prospects toward biocompatible and visible‐light‐controlled supramolecular robotic systems.

## 2 Results and Discussion

### 2.1 Molecular Design and Synthesis

**Scheme 1:**
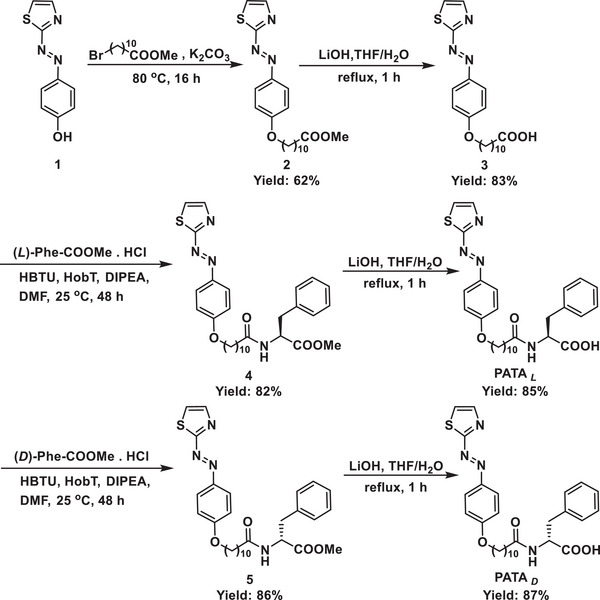
Synthesis of PATAs.

PATA was designed and functionalized with a phenylalanine amino acid end‐group, connected with a decanyl‐linker to the phenyl motif of the phenylazothiazole molecular switching core. Compound **1** was prepared according to the previously reported procedure and followed by a Williamson ether formation in the presence of alkyl bromide and potassium carbonate in dimethylformamide. After the basic deprotection of compound **2**, the obtained compound **3** was employed for an amide‐bond coupling reaction with either *L*‐phenylalanine methyl ester or *D*‐phenylalanine methyl ester, mediated by Hexafluorophosphate Benzotriazole Tetramethyl Uronium. **PATA*_L_*** and **PATA*_D_*** were obtained by hydrolysis of ester groups of compounds **4** and **5**, respectively, in the presence of lithium hydroxide in water/tetrahydrofuran mixture (**Scheme** **1**). The chemical structures of all newly obtained compounds and PATAs were unambiguously characterized by ^1^H, ^13^C nuclear magnetic resonance (NMR) and high‐resolution electrospray ionization time‐of‐flight mass spectrometry (Figures S34−S43, Supporting Information).

### 2.2 Photoisomerization of PATA*_L_*

**Figure 2:**
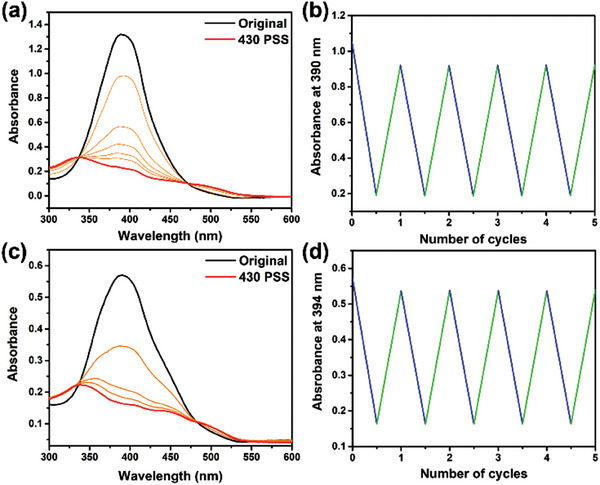
UV–vis absorption spectra of **PATA*_L_*** (50 µm), *E*‐**PATA*_L_*** to *Z*‐**PATA*_L_*** in a) MeOH and c) water. b) The change in UV–vis absorption for **PATA*_L_*** (50 *μ*M) in b) MeOH and d) water after five blue light irradiations (blue‐line)/green light irradiations (green‐line) cycles.

Photoisomerization of **PATA*_L_*** was examined by UV–vis spectroscopy and ^1^H NMR. A methanol (MeOH) solution of **PATA*_L_*** (50 µm) showed a strong absorption band at 380−410 nm in the UV–vis absorption spectrum at 20 °C (**Figure** **2**a, black‐line). Upon 430 nm blue light irradiation, increased absorbance was observed at 480−550 nm, along with a concomitant decrease in the absorption band at 380−410 nm was observed (Figure 2a, red‐line). In addition, two observed clear isosbestic points at 336 and 472 nm over the course of irradiation suggested that a selective photoisomerization process from *E*‐**PATA*_L_*** to *Z*‐**PATA*_L_*** occurred. The irradiated solution was then subjected to a reverse photoswitching process to *E*‐**PATA*_L_*** by 530 nm green light irradiation (Figure S1, Supporting Information). The photoswitching cycles between *E*‐**PATA*_L_*** and *Z*‐**PATA*_L_*** were repeated by consecutive 430 and 530 nm irradiation for 5 more cycles, resulting in no notable signs of fatigue (Figure 2b). The identical photoswitching processes of **PATA*_L_*** in a methanol‐d4 solution (5 mm) were monitored using ^1^H NMR study. ^1^H NMR spectral changes were observed in selected aromatic and aliphatic regions upon irradiation of 430 and 530 nm alternately for 15 min (Figure S2, Supporting Information). The triplet signal for the aliphatic proton had downfield‐shifted from *δ* = 4.13 ppm to *δ* = 4.04 ppm, indicating a 430 nm blue light‐induced isomerization process from *E*‐**PATA*_L_*** and *Z*‐**PATA*_L_*** (Figure S2a,b, Supporting Information). The photostationary state (PSS) ratio driven by 430 nm blue light was determined as 80:20 (*E*‐**PATA*_L_***/*Z*‐**PATA*_L_***). The *E*‐**PATA*_L_*** could nearly all be regenerated by further irradiation with 530 nm green light to give the PSS ratio as 96:4 (*E*‐**PATA*_L_***/*Z*‐**PATA*_L_***) (Figure S2b,c, Supporting Information). A circular dichroism (CD) spectroscopic study was further employed to examine the effect of point chirality associated with the presence of *L*‐phenylalanine moiety. A MeOH solution of **PATA*_L_*** (50 µm) showed no CD signal at 300−500 nm (Figure S3, Supporting Information). The result suggested that no significant supramolecular helicity was induced around the phenylazothiazole motif in the organic medium.

An aqueous solution of **PATA*_L_*** was prepared by dissolution of **PATA*_L_*** into deionized water in the presence of two equivalents of sodium hydroxide, the resulting solution was subjected to heating at 80 °C for 10 min and cooled to 20 °C at a rate of 5 °C min^−1^ (thermal annealing) to afford a clear orange solution. Compared to the UV–vis spectrum in the MeOH solution of **PATA*_L_***, an aqueous solution of **PATA*_L_*** (50 µm) showed a broadened absorption band at 380−450 nm at 20 °C (Figure 2c, black‐line) with a slight bathochromic shift (≈4 nm) of the absorption maxima. Upon 430 nm photoirradiation, absorption maxima at ≈394 nm decreased and showed two clear isosbestic points at 337 and 478 nm, respectively (Figure 2c, red line), indicating a selective photoisomerization process from *E*‐**PATA*_L_*** to *Z*‐**PATA*_L_*** occurred in aqueous medium. Followed by 530 nm green light irradiation, the irradiated solution showed a reverse photoswitching process to *E*‐**PATA*_L_*** (Figure S4, Supporting Information). The photoirradiation cycles in aqueous medium were repeated by consecutive 430 and 530 nm irradiation for five more cycles and no notable sign of fatigue was observed (Figure 2d). It is noted that no significant difference was found for the photoswitching behavior of **PATA*_L_*** between organic solvents and water, indicating that molecular changes could be controlled selectively and effectively by light. This further allows for in‐depth investigations on visible‐light‐induced molecular geometrical transformations of **PATA*_L_*** at multiple length scales in aqueous medium.

### 2.3 Supramolecular Assembly and Visible‐Light‐Induced Supramolecular Transformation of PATA

**Figure 3:**
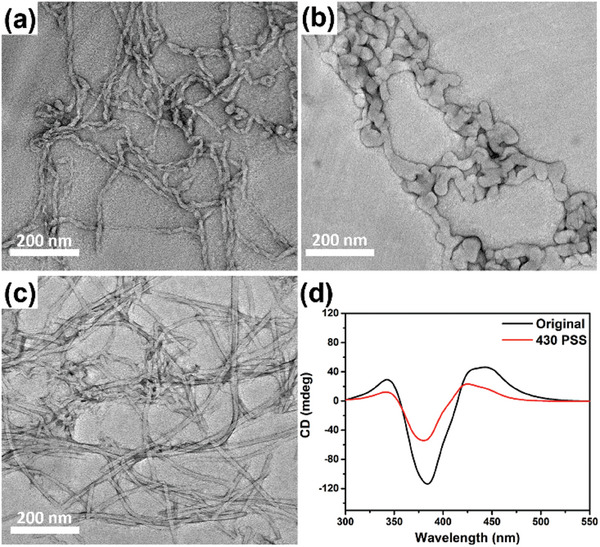
TEM images of an aqueous solution of **PATA*_L_*** a) before, b) after 430 nm blue light irradiation, and c) subsequently followed by 530 nm green light irradiation with sonication. d) CD spectra of **PATA*_L_*** over the course of irradiation with different light sources.

Thermally annealed aqueous solution of **PATA*_L_*** was analyzed by laser confocal Raman microscopy, and new vibrational peaks were observed at ≈1000 and 1250 cm^−1^, indicating the possible formation of supramolecular aggregates (Figure S5, Supporting Information). An aqueous solution of **PATA*_L_*** was prepared by the identical method mentioned previously. The critical aggregation concentration (CAC) of **PATA*_L_*** was examined by using a Nile Red Fluorescence Assay, which probes the internal hydrophobicity of the assemblies. A notable blueshift of the emission wavelength of Nile Red is observed when aqueous solutions of PATAs are diluted below 0.01 mm, and the CAC of **PATA*_L_*** was determined to be ≈1.0 µm (Figure S6, Supporting Information). It is noted that the concentration used for absorption spectroscopic analysis is above CAC, suggesting that the photoisomerization process of **PATA*_L_*** could possibly occur in the assembled states in aqueous medium (Figure 2; Figure S4, Supporting Information). To further investigate the photoisomerization processes in aqueous media, a freshly prepared solution of **PATA*_L_*** at a concentration of 3.73 mm, above the CAC, was used to analyze the supramolecular assembled structures by using transmission electron microscopy (TEM) to capture the solution‐state morphologies of **PATA*_L_***. It is found that **PATA*_L_*** assembles into helical nanofibers from hundreds of nanometers to micrometers in length and ≈13.4 ± 0.9 nm in diameter (**Figure** **3**a; Figure S7, Supporting Information). Upon irradiation with 430 nm blue light, the supramolecular transformation from helical nanofibers to irregular aggregates with a thicker diameter ≈32.4 ± 2.2 nm are observed (Figure 3b; Figure S8, Supporting Information), suggesting that the formation of *Z*‐**PATA*_L_*** destabilized the assembled structure and induced assembly transformations. A subsequent 530 nm green light irradiation of **PATA*_L_*** solution showed that the dissembling irregular aggregates had further transformed into plate‐like irregular structures (Figure S9, Supporting Information). Followed by sonication of the same solution, plate‐like irregular structures could be broken down and reformed into nanofibers with a diameter ≈11.4 ± 2.0 nm (Figure 3c; Figure S10, Supporting Information), indicating that the plate‐like irregular structures could be possibly associated with heavily entangled and aggregated nanofibers.

To further examine the supramolecular transformations of **PATA*_L_***, a CD spectroscopic analysis was conducted to monitor possible changes in the helicity of the assembled structures upon alternative 430 and 530 nm light irradiation (Figure 3d). An aqueous solution of **PATA*_L_*** (50 µm) shows a strong band at 383 nm with zero‐crossing of the negative Cotton effect at 295 and 355 nm along with two bands at 342 and 440 nm of the positive Cotton effect (Figure 3d, black‐line). Upon irradiation with 430 nm blue light, both negative and positive Cotton effects are reduced significantly in the CD spectrum (Figure 3d, red‐line), the dramatic spectral changes could be originated from the light‐triggered dissembling process as revealed in TEM investigations (Figure 3b). Further irradiation with 530 nm green light could not recover the spectral shifts back to their original (Figure S11, Supporting Information), indicating loss of helicity with destructed supramolecular structures upon isomerization process as observed by TEM (Figure 3c). The results showed that the manipulation of molecular geometry with photoirradiation can induce supramolecular transformations of **PATA*_L_***, allowing for the control of supramolecular assemblies from nanometers to hundreds of nanometers length scales systematically.

The photoisomerizations of **PATA*_D_*** (50 µm) were observed essentially identical in both organic and aqueous media (Figure S12–S13, Supporting Information) to that observed in **PATA*_L_*** (Figure 2). Besides, the CD spectrum of **PATA*_D_*** (50 µm, MeOH) shows no obvious helicity (Figure S14a, Supporting Information). An aqueous solution of **PATA*_D_*** (50 µm) shows a complementary CD Cotton effect to **PATA*_L_*** (Figure S14b, Supporting Information), while racemic mixture of **PATA*_L_*** and **PATA*_D_*** (50 µm) showed no CD signal (Figure S14c, Supporting Information). TEM images of an aqueous solution of **PATA*_D_*** (50 µm, Figure S15, Supporting Information) were observed with similar helical nanofibers to **PATA*_L_*** (Figure S7, Supporting Information). Upon irradiation with 430 nm blue light, helical nanofibers of **PATA*_D_*** converted to irregular aggregates with increased diameter (Figure S16, Supporting Information). The CD and TEM results indicated that there is no significant variation of supramolecular nanostructure formation and transformation observed between the pair of enantiomers of PATA.

### 2.4 Structural Properties and Macroscopic Actuation Mechanism of Supramolecular Macroscopic Soft Scaffolds of PATA

According to our previously reported preparation method of macroscopic soft scaffolds, an aqueous solution of **PATA*_L_*** (5.0 weight% (wt%), 93.2 mm), after thermal annealing, was ejected into a shallow pool of calcium chloride (CaCl_2_) solution (150 mm) to afford a yellow macroscopic soft scaffold with ≈450 µm in diameter (Figure S17a, Supporting Information). After rinsing with water, the obtained scaffold was imaged under optical microscopy (OM) with or without a polarizer, showing birefringent with partial unidirectional alignment of the supramolecular helical nanofibers (Figure S17b, Supporting Information).

**Figure 4:**
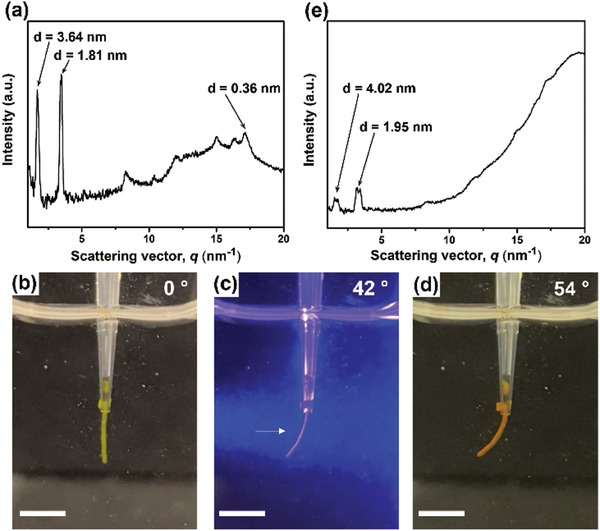
1D‐WAXD pattern of a macroscopic soft scaffold of **PATA*_L_*** (5.0 wt%, 93.2 mm) a) before and e) after 430 nm blue light irradiation, showing the diffraction pattern perpendicular to the long axis of the scaffold. Snapshots of macroscopic soft scaffold of **PATA*_L_*** (5.0 wt%) in CaCl_2_ (150 mM) b) before and after irradiation with 430 nm blue light source from the left side for c) 60 s and d) 120 s. The macroscopic soft scaffold of **PATA*_L_*** bends toward the blue light. Scale bars for all panels: 0.5 cm.

Scanning electron microscopy images of an air‐dried macroscopic soft scaffold of **PATA*_L_*** (5.0 wt%) show arrays of helical nanofibers partially aligned to the longer axis of the scaffold (Figure S18a, Supporting Information). The microscopic imaging results revealed a partial unidirectional alignment of helical nanofibers of **PATA*_L_*** to the longer axis of the scaffold. Notably, the surface morphology of the macroscopic soft scaffold of **PATA*_L_*** with 3.0 wt% (Figure S18b, Supporting Information) and 1.0 wt% (Figure S18c, Supporting Information) were similar to 5.0 wt% (Figure S18a, Supporting Information). A macroscopic soft scaffold of **PATA*_L_***, prepared from the identical method, was transferred into a capillary, and sealed to maintain stable humidity for X‐ray diffraction (XRD) studies. Using through‐view small‐angle X‐ray scattering (SAXS), the 2D‐SAXS image of the macroscopic soft scaffold of **PATA*_L_*** was observed without any sign of directional scattering in the region of *q* = 0.1–0.45 nm^−1^ (Figure S19a, Supporting Information). 1D‐SAXS pattern of the macroscopic soft scaffold of **PATA*_L_***, converted from the 2D‐SAXS image, shows diffraction peaks with *d*‐spacings of 3.84 and 1.92 nm (Figure S19b, Supporting Information). Using through‐view wide‐angle X‐ray diffraction (WAXD), the 2D‐WAXD image of the macroscopic soft scaffold of **PATA*_L_*** was similarly observed without a sign of directional diffraction (Figure S20a, Supporting Information). The 1D‐WAXD pattern of the macroscopic soft scaffold of **PATA*_L_*** shows a similar pair of diffraction peaks with *d*‐spacings of 3.64 and 1.81 nm (**Figure** **4**a; Figure S20c, Supporting Information) to that observed in SAXS (Figure S19b, Supporting Information). The pair of diffraction peaks originated from the diffraction of the head‐to‐head packed bilayer structure of **PATA*_L_*** in the supramolecular helical nanofibers. Enhanced *π*–*π* interaction of phenylazothiazole cores was observed from a diffraction peak with *d*‐spacing of 0.36 nm (Figure S20b, Supporting Information). Other diffraction peaks with *d*‐spacings of 0.76, 0.52, 0.42, and 0.38 nm originated from the higher packing order of helical nanofibers (Figure S20c, Supporting Information). The XRD results revealed the supramolecular packing of **PATA*_L_*** in the supramolecular helical nanofibers, though no alignment of supramolecular packing of **PATA*_L_*** was observed, due to a low degree of unidirectional alignment, the intrinsic helical supramolecular structure averaging, and minimizing the occurrence of aligned packing structure.

To examine the functional properties of **PATA*_L_***, a macroscopic soft scaffold was prepared in a cuvette with CaCl_2_ solution (150 mm), using a shear‐flow method. Macroscopic soft scaffolds of **PATA*_L_*** can be attached to the side of a cuvette (Figure 4b). Upon 430 nm blue light irradiation from the left side of the macroscopic soft scaffold of **PATA*_L_***, the scaffold was bending toward the direction of the light source (Figure 4b–d; Movie S1, Supporting Information) over 120 s, in attaining a macroscopic actuation of 54° (actuation speed = 0.43 ± 0.02° s^−1^). Noticeably, this is the first macroscopic actuation of a supramolecular robotic system of photoresponsive amphiphiles driven by blue light. With the advanced macroscopic actuation of the macroscopic soft scaffold of **PATA*_L_*** in aqueous media, a scaffold was subjected to WAXD study after 430 nm blue light irradiation. The obtained 2D‐WAXD image shows no sign of directional alignment (Figure S21a, Supporting Information). The converted 1D‐WAXD pattern shows significant diffraction peak shift with *d*‐spacings from 3.64 and 1.81 nm (Figure 4a) to 4.02 and 1.95 nm (Figure 4e), indicating a significantly increased distance of the head‐to‐head packed bilayer structure of **PATA*_L_***, upon 430 nm blue light irradiation. Photoisomerization of *E*‐**PATA*_L_*** to *Z*‐**PATA*_L_*** reduces its structural planarity and *π*–*π* interaction of phenylazothiazole cores, extending the head‐to‐head packed bilayer structure of **PATA*_L_*** (Figure S21b, Supporting Information). The increased distance of the bilayer structure of **PATA*_L_*** can destabilize the supramolecular helical nanofibers, consequently, significantly broadened helical nanofibers were observed in TEM (Figure 3a,b). Hence, the higher‐order diffraction peaks were diminished after photoirradiation.

Additionally, a macroscopic soft scaffold in a cuvette was irradiated with two 430 nm blue light sources from both left and right sides (Figure S22a; Movie S2a, Supporting Information) to show a linear contraction and shortened length of the scaffold. Besides, a subtle decrease in the diameter of the photoirradiated supramolecular soft scaffold of **PATA*_L_*** was observed. By mixing a 3.5 wt% annealed solution of **PATA*_L_*** and a FluoSpheres Polystyrene Microspheres suspension at a 6:1 volume ratio, it yielded a 3.0 wt% solution of **PATA*_L_*** as the final concentration. The microbead was observed under fluorescent microscopy with an excitation 470 nm light source. The microbead encapsulated macroscopic soft scaffold of **PATA*_L_*** was bent toward the 430 nm blue light source, accompanied by the movements of the microbeads without any observable leakage of microbeads (Figure S22b, Supporting Information). More importantly, the diameter of the scaffold decreased from 596 to 462 µm approximately (Figure S22b, Movie S2b, Supporting Information). The results revealed that both the longer axis and shorter axis of the supramolecular soft scaffold are contracted throughout the macroscopic actuation process, suggesting the total volume of the scaffold is reduced. Given that the distance of a particular encapsulated microbead, highlighted in Figure S22b (Supporting Information), is measured between the left (281 µm) and right (248 µm) edges of the supramolecular soft scaffold. After 430 nm blue light irradiation for 120 s, the distance between the microbead and the left edge of the scaffold was decreased significantly to 148 µm, while the distance between the microbead and the right edge of the scaffold was slightly increased to 257 µm, indicating that the microbead was moved toward the left edge of the scaffold (irradiated direction). The results indicated that the photoirradiated edge of the supramolecular soft scaffold contracted predominantly over the non‐irradiated edge of the scaffold, due to the difference in light‐penetration gradient in the scaffold with ≈450 µm in thickness. Considering only partial unidirectional alignment of supramolecular helical nanofibers of **PATA*_L_*** observed in the supramolecular soft scaffold, upon photoisomerization, the disassembled irregular aggregates of **PATA*_L_*** could not retain the entrapped water within its supramolecular soft scaffold, due to the loss of large‐aspect‐ratio structural properties. Entrapped water should be ejected to the outer surface of the scaffold, and resultantly, the macroscopic contraction on the photoirradiated side is faster than the non‐irradiated side along the light penetration gradient to offer macroscopic actuations. To examine the effect of the nanostructure orientation of the scaffold on attaining macroscopic actuation, an isotropic scaffold of **PATA*_L_*** (3.0 wt%) was irradiated with blue light for 60 s. Isotropic shrinkage of the scaffold was observed over the course of irradiation, suggesting that nanostructure orientation is one of the key parameters for controlling the degree of actuation of the macroscopic scaffold of **PATA*_L_*** (Figure S23, Supporting Information).

### 2.5 Macroscopic Actuations of Supramolecular Macroscopic Soft Scaffolds of PATA and Related Biocompatibility

**Figure 5:**
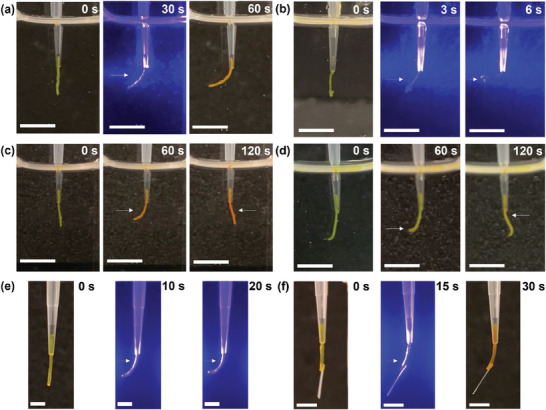
a) Snapshots of a macroscopic soft scaffold of **PATA*_L_*** (3.0 wt%) in CaCl_2_ (150 mm) after irradiation with 430 nm blue light source from left side for 60 s. b) Snapshots of a macroscopic soft scaffold of **PATA*_L_*** (1.0 wt%) in CaCl_2_ (150 mm) after irradiation with 430 nm blue light source from left side for 6 s. c) Snapshots of a macroscopic soft scaffold of **PATA*_L_*** (3.0 wt%) in CaCl_2_ (150 mm) after sequential 430 nm blue‐irradiation from alternate sides to demonstrate the scaffold was actuated toward the light source according to the irradiation direction. d) Snapshots of a macroscopic soft scaffold of **PATA*_L_*** (3.0 wt%) in CaCl_2_ (150 mm) after sequential point irradiation from alternate directions, resulting in a zigzag conformation. Snapshots of a macroscopic actuation of a macroscopic soft scaffold of **PATA*_L_*** (3.0 wt%) after irradiation with 430 nm blue light source in the air e) without weight and f) with 0.2 mg paper as weight. The direction of irradiation is indicated by white arrows. Scale bars for all panels: 0.5 cm.

With the interpretation macroscopic actuation mechanism of **PATA*_L_***, different macroscopic actuation studies were performed in air and aqueous media. A thermally annealed aqueous solution of **PATA*_L_*** (3.0 wt%, 55.9 mm) was prepared into a macroscopic soft scaffold and subjected to macroscopic actuation (**Figure** **5**a; Movie S3, Supporting Information), showing 79° flexion angle with a faster actuation speed 1.39 ± 0.12° s^−1^. Additionally, the scaffold color was changed from yellow to orange, due to *E*‐**PATA*_L_*** to *Z*‐**PATA*_L_*** photoisomerization. A macroscopic soft scaffold of **PATA*_L_*** (1.0 wt%, 18.6 mm) showed the fastest blue light‐driven macroscopic actuation with actuation speed = 17 ± 1.7° s^−1^ (Figure 5b; Movie S4, Supporting Information). Importantly, this is a faster macroscopic actuation speed compared to previously reported motor amphiphiles with carboxylate end groups (7.9 ± 0.4° s^−1^)^[^ ^59^ ^]^ and phosphite end groups (1.5 ± 0.2° s^−1^),^[^ ^60^ ^]^ made from merely 1.0 wt% supramolecular assembled materials. Higher concentrations of **PATA*_L_*** in the fabricated macroscopic soft scaffolds hinder the macroscopic actuation, due to higher structural rigidity and lower water content within scaffolds. Macroscopic soft scaffolds of **PATA*_L_*** with 3.0 wt% can afford stable macroscopic structure with faster macroscopic actuation. Atomic force microscopy (AFM) was utilized to analyze the mechanical properties of the macroscopic soft scaffolds of **PATA*_L_*** with different concentrations, the young modulus of macroscopic soft scaffolds of **PATA*_L_*** with 5.0, 3.0, and 1.0 wt% were 14.2 ± 5.4, 8.44 ± 2.03, and 1.95 ± 0.17 kPa, respectively. The results suggested that macroscopic soft scaffolds of **PATA*_L_*** with a lower elasticity would result in a faster macroscopic actuation speed. Macroscopic soft scaffolds of **PATA*_L_*** (3.0 wt%) were prepared from CaCl_2_ solution with different concentrations (20–100 mm, Figure S24, Supporting Information), and subjected to macroscopic actuation. The scaffolds of **PATA*_L_*** can be formed with the lowest CaCl_2_ concentration (20.0 mm) to attain actuation speed = 1.26 ± 0.13° s^−1^ (Figure S24a, Supporting Information). The elasticity of the macroscopic scaffolds of **PATA*_L_*** prepared by different concentrations of calcium ions was similar to that of observed for scaffolds prepared from 150 mm calcium ions (Table S1, Supporting Information), and consequently a similar actuation speed was attained (Figure S24b,c, Supporting Information). The actuation speed of macroscopic soft scaffolds of **PATA*_L_*** (3.0 wt%) was further examined with different intensities of blue light irradiation, the results revealed that faster actuation speed could be attained with increased intensity of light source, possibly due to higher occurrence of photoisomerization of **PATA*_L_*** (Table S2, Supporting Information). After optimization of macroscopic soft scaffold preparation, a macroscopic soft scaffold of **PATA*_L_*** (3.0 wt% in 150 mm CaCl_2_ solution) was irradiated with 430 nm blue light from left and right sides sequentially for 60 s (Figure 5c; Movie S5, Supporting Information), in which the scaffold was actuated toward to the light source according to the irradiation direction. Utilizing alternative irradiation from left and right sides, the actuation response of macroscopic soft scaffolds of **PATA*_L_*** with 5.0 and 3.0 wt% was hampered after 4 cycles (Figure S25a,b, Supporting Information). Notably, only two cycles could be attained for macroscopic soft scaffolds of **PATA*_L_*** with 1.0 wt% (Figure S25c, Supporting Information), possibly due to the gradual loss of structural properties over the course of irradiation.

Besides, multiple‐point actuation of macroscopic soft scaffolds of **PATA*_L_*** was demonstrated with optical‐fiber coupled 430 nm blue light irradiation (Figure 5d; Movie S6, Supporting Information), indicating potential precise robotic functions feasibility. The structural stability of the macroscopic soft scaffolds of **PATA*_L_*** enables macroscopic actuation in air. A macroscopic soft scaffold **PATA*_L_*** prepared in CaCl_2_ solution (150 mm) was taken out of the surface of the aqueous solution and held onto the sample holder (Figure 5e; Movie S7, Supporting Information). The scaffold was actuated toward the light source with a flexion angle of 90° within 30 s, revealing a faster actuation speed = 4.50° s^−1^ than that of observed in aqueous media, possibily due to light scattering in aqueous media (Figure 5a). A piece of paper weighted 0.2 mg was installed onto the surface of a macroscopic soft scaffold of **PATA*_L_*** in air and attained 30° actuation flexion angle in 30 s (Figure 5f; Movie S8, Supporting Information), upon 430 nm blue light irradiation. The calculated work done for the weightlifting process was ≈2.35 nJ (Figure S26, Supporting Information). Grasping and releasing of a 0.2 mg paper were further demonstrated by photoactuation of the macroscopic soft scaffold **PATA*_L_*** (Figure S27, Movie S9, Supporting Information).

With the demonstrated macroscopic actuations related to *E*‐**PATA*_L_*** to *Z*‐**PATA*_L_*** isomerization driven by blue light, the backward isomerization driven by 530 nm green light was investigated. A macroscopic soft scaffold of **PATA*_L_*** was irradiated and actuated with 430 nm blue light for 30 s with 60° flexion angle, it was further irradiated with 530 nm green light for 60 s on the same side and no further actuation observed based on the flexion angle (Figure S28, Supporting Information). It is noted that the scaffold was changed from orange to yellow again after green light irradiation. Besides, a scaffold of **PATA*_L_*** was irradiated solely with 530 nm green light and actuated toward the light source (Figure S29, Supporting Information), possibly due to broad excitation of the green light source. In this connection, a scaffold of **PATA*_L_*** was irradiated with a narrow excitation green light laser (≈530 nm) and showed no macroscopic actuation (Figure S30a, Supporting Information), while the scaffold was bent toward 430 nm blue‐laser source (Figure S30b, Supporting Information). The results suggested that the backward photoisomerization from *Z*‐**PATA*_L_*** to *E*‐**PATA*_L_*** is feasible at the macroscopic length scale, though no obvious macroscopic actuation can be attained, possibly due to the disorganized supramolecular packing after blue light‐driven macroscopic actuation. Finally, a thermally annealed aqueous solution of **PATA*_D_*** (3.0 wt%, 55.9 mm) was prepared into a macroscopic soft scaffold in a cuvette and irradiated with 430 nm blue light (Figure S31 and Movie S10, Supporting Information), in affording macroscopic actuation (1.28° s^−1^) similar to its enantiomer **PATA*_L_***. The similar macroscopic actuation of both enantiomers of PATA suggested a similar actuation mechanism is employed. The helical nanofibers of PATA, regardless of their structural helicity, were prepared from a shear‐flow method into the non‐helical macroscopic soft scaffold, resultantly no hardness‐biased actuation was observed. Additionally, aqueous solutions of **PATA*_L_*** and **PATA*_D_*** (**PATA*_L+D_***, 5.0 wt%, 1:1) were mixed and the resulting racemic mixture was further thermally annealed to show isotropic hydrogel‐like materials under polarized OM (Figure S32a, Supporting Information). No obvious macroscopic movement was observed for **PATA*_L+D_*** scaffold, due to loss of unidirectional alignment (Figure S32b, Supporting Information).

**Figure 6:**
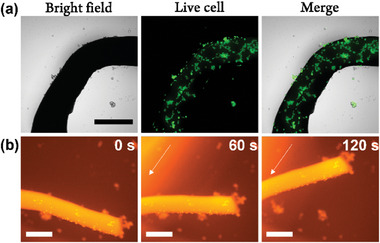
a) Live cell staining of hMSCs cultured with macroscopic soft scaffold of **PATA*_L_*** (3.0 wt%). After cell culture for 2 h, live cells were stained by 2 µm CalceinAM. b) Snapshots of hMSCs (CellTracker Orange CMRA Dye labeled) adhered macroscopic soft scaffold of **PATA*_L_*** (3.0 wt%) after irradiation with 450 nm blue light laser from left side for 120 s. The direction of irradiation is indicated by white arrows. Scale bar: 500 µm.

Given that macroscopic soft scaffolds of **PATA*_L_*** can be fabricated with a low concentration of CaCl_2_ solution (≈20 mm), a thermally annealed solution of **PATA*_L_*** (3.0 wt%) was ejected into αMEM medium (supplemented with 10% Fetal bovine serum and 1% Antibiotic‐Antimycotic). Note that an unmodified αMEM medium (Gibco) contains ≈1.8 mm calcium ion. A stable macroscopic soft scaffold (Figure S33a, Supporting Information) was observed. Human mesenchymal stem cells (hMSCs) have been an attractive research target in regenerative medicine due to their multipotency.^[^ ^18^ ^]^ We envisaged that the macroscopic soft scaffold of **PATA*_L_*** could serve as a material surface for cell culture. hMSCs were seeded and incubated for 5 h to show significant cell adhesion onto the surface of the scaffold (Figure S33b,c, Supporting Information). To demonstrate the versatility of macroscopic soft scaffolds of **PATA*_L_*** with different cell types, muscle cells (C2C12 myoblasts) were incubated and showed obvious cell attachment onto the surface of macroscopic soft scaffold of **PATA*_L_*** (Figure S33d, Supporting Information). Live hMSCs attachment was confirmed by Calcein AM staining after 2 h of incubation with **PATA*_L_*** (**Figure** **6**a), which suggested the biocompatibility macroscopic soft scaffold of **PATA*_L_***.

According to Leung and Feringa previously reported supramolecular soft robotics of motor amphiphiles,^[^ ^60^ ^]^ the macroscopic soft scaffold of motor amphiphiles could be actuated with UV light after cell attachment. To evaluate the photoactivation property of the blue light‐driven supramolecular robotic system after cell attachment, a hMSCs attached macroscopic soft scaffold of **PATA*_L_*** was irradiated with 450 nm blue light laser and showed obvious macroscopic actuation with accompanied movements of labeled hMSCs in situ (Figure 6b; Movie S11, Supporting Information), revealing the potential cell‐material interface with controllable movements. In this connection, the cell studies results revealed good biocompatibility and cell attachment of the macroscopic soft scaffold of **PATA*_L_***. Material systems with macroscopic actuation function might provide mechanical stimulation to cells, which provides a promising opportunity for regenerative medicine. For instance, a recent report by Mooney et al. demonstrated the prevention of muscle atrophy by material actuation‐induced mechanotransduction.^[^ ^72^ ^]^ **PATA*_L_*** soft scaffold comprises small molecules precisely organized by supramolecular assembling processes, the visible light‐induced macroscopic actuation is merited, due to the non‐necessity of any electrical conducting material component. We envision that the photo‐mechanical conversion of biocompatible **PATA*_L_*** macroscopic soft scaffold could serve as the next generation of artificial implant biomaterials, enabling remotely controlled mechanotransduction for regenerative medicine.

## 3 Conclusion

Phenylazothiazole amphiphile functionalized with a phenylalanine‐charged end‐group was designed for fabricating macroscopic soft scaffolds. Macroscopic actuation of the macroscopic soft scaffolds of **PATA*_L_*** were successfully illustrated with the intrinsic amplification of synchronized molecular motions across multiple length scales. Detailed macroscopic actuation mechanism, induced by the selective *E*‐**PATA*_L_*** to *Z*‐**PATA*_L_*** photoisomerization of the phenylazothiazole core embedded in oriented supramolecular assembled structure in aqueous media, was meticulously confirmed with UV‐vis absorption, electron microscopy, NMR spectroscopy, and XRD measurements. The actuation speed of macroscopic soft scaffolds of **PATA*_L_*** was optimized to realize a rapid macroscopic actuation of supramolecular robotic system with actuation speed up to 17 ± 1.7° s^−1^. Weightlifting and localized actuation experimental demonstrations showed the functional versatility of macroscopic soft scaffold of **PATA*_L_***, in serving as supramolecular robotic materials. Macroscopic soft scaffolds of **PATA*_L_*** were further demonstrated with live‐cell attachment and the accompanied movements have laid the foundation for the future development of remotely controlled mechanotransduction biomaterials. This work could develop propitiously toward visible light‐responsive biomaterials with muscle mimetic actuation for mechanically guided stem cell differentiation and regenerative medicine. For perspective, we will continue the exploration of the performance of **PATA*_L_*** as a biomaterial option for small cargo delivery and biomedical applications, such as mechanotherapy.

## Conflict of Interest

The authors declare no conflict of interest.

## Author Contributions

M.H.C. performed all the synthesis and most of the experiments for PATA in this work, except W.K.W. performed cell‐material interface and AFM studies and T.K. performed XRD analysis. M.H.C., W.K.W. drafted the paper and F.K.‐C.L. reviewed and edited the paper, conceived and supervised the research. All authors discussed the results and commented on the manuscript.

## Supporting information



Supporting Information

Supplemental Movie 1

Supplemental Movie 2a

Supplemental Movie 2b

Supplemental Movie 3

Supplemental Movie 4

Supplemental Movie 5

Supplemental Movie 6

Supplemental Movie 7

Supplemental Movie 8

Supplemental Movie 9

Supplemental Movie 10

Supplemental Movie 11

## Data Availability

The data that support the findings of this study are available in the supplementary material of this article.
