# Supplementary material for: Blue Light Controlled Supramolecular Soft Robotics of Phenylazothiazole Amphiphiles for Rapid Macroscopic Actuations
Source: Adv Sci (Weinh). 2024 Oct 16;11(45):2407130. doi: 10.1002/advs.202407130 (PMC11615827; doi:10.1002/advs.202407130)
Supplement: Supplementary file 1 — Supporting Information [file ADVS-11-2407130-s003.pdf]

ADVANCED SCIENCE
Open Access

Supporting Information

for *Adv. Sci.*, DOI 10.1002/advs.202407130

Blue Light Controlled Supramolecular Soft Robotics of Phenylazothiazole Amphiphiles for Rapid Macroscopic Actuations

*Ming-Hin Chau, Wai-Ki Wong, Takashi Kajitani and Franco King-Chi Leung**

Supporting Information

Blue-Light Controlled Supramolecular Soft Robotics of Phenylazothiazole Amphiphiles for Rapid Macroscopic Actuations

Ming-Hin Chau,[a] Wai-Ki Wong,[a,b] Takashi Kajitani,[c] and Franco King-Chi Leung*[a,b]

[a] State Key Laboratory of Chemical Biology and Drug Discovery, Research Institute for Future Food, Department of Applied Biology and Chemical Technology, The Hong Kong Polytechnic University, Hong Kong, China.
[b] Centre for Eye and Vision Research, 17W Hong Kong Science Park, Hong Kong, China.
[c] Open Facility Development Office, Open Facility Center, Tokyo Institute of Technology, 4259 Nagatsuta, Midori-ku, Yokohama 226-8503, Japan.

Email: kingchifranco.leung@polyu.edu.hk

Table of Content

General 2
Materials and methods 3
Description of supplementary movies 6
Synthesis 8
Supporting Figures 12
Analytical Data 47
References 52

General

^{1}H NMR and ^{13}C NMR spectra were recorded at 298 K on Bruker Advance III Ultrashield 400 Plus NMR spectrometer (^{1}H: 400 MHz, ^{13}C: 101 MHz) and Bruker Advance III Ultrashield 600 Plus NMR spectrometer (^{1}H: 600 MHz, ^{13}C: 151 MHz). Chemical shifts (δ) are expressed relative to the resonances of the residual non-deuterated solvent for ^{1}H [tetramethylsilane (TMS) for $CDCl_3$: $^{1}H(\delta)$ = 0.00 ppm, CD_3CN: $^{1}H(\delta)$ = 1.94 ppm] and ^{13}C [$CDCl_3$: $^{13}C(\delta)$ = 77.16 ppm, CD_3CN: $^{13}C(\delta)$ = 1.32 ppm and 118.26 ppm]. Absolute values of the coupling constants are given in Hertz (Hz), regardless of their sign. Multiplicities are abbreviated as singlet (s), doublet (d), doublet of doublets (dd), triplet (t), and multiplet (m). High-resolution mass spectrometry (HR-MS) was performed on Agilent 6540 UHD Accurate-Mass Q-TOF LC/MS system with ESI ionization.

Materials and methods

All commercial reagents are purchased from Acros Organics, Aladdin, Alfa Aesar, Bidepharm, Dieckmann, Macklin, Sigma Aldrich and Tokyo Chemical Industry Co. Ltd, and were used as received unless otherwise specified. All reactions were performed under nitrogen unless otherwise specified. Analytical thin layer chromatography (TLC) was performed with Macherey-Nagel Silica gel 60 UV254 aluminum plates and visualization was accomplished by UV light (254 / 365 nnm) or staining with phosphomolybdic acid followed by heating. Flash column chromatography was performed using Macherey-Nagel Silica gel 60 (230-400 mesh). Deuterated solvents were purchased from Cambridge Isotope Laboratories Inc. All cell culture reagents were purchased from Thermofisher unless specified.

UV-Vis Spectroscopy. UV-vis measurements were performed on Agilent Cary 60 UV-Visible Spectrophotometer with a 1 cm path length quartz cuvette. A Luma 40/ Cary 60 temperature-controlled cuvette holder with four optical ports was mounted in the sample compartment of Agilent Cary 60 UV-Visible Spectrophotometer. Measurement of all samples were carried out at 20 °C unless otherwise specified.

Preparation of aqueous sample. PATA (5.0 wt.%) was dissolved in fresh deionized water (DI water). The solution was heated at 80 °C for 10 min, then slowly cooled to 20 °C at a rate of 5.0 °C/min to form assembled structure. For TEM study, the annealed solution was further diluted to 0.2 wt.%.

Actuation experiments in water and air. The aqueous solution of **PATA** prepared by the above method was manually drawn into an aqueous solution of $CaCl_2$ (150 mM) from a pipette. The obtained noodle-like string was kept in solution or pulled out of the water and suspended onto a sample holder for actuation experiments. Irradiation studies were performed with a Thorlabs model M430F1 light-emitting diode (5.3 mW) and point irradiation studies were performed with a Thorlabs model M430F1 light-emitting diode coupled with a SMA-SMA Solarization-Resistant MM Fiber Patch Cable (Ø400 μm, 0.22 NA). The distances between the light source and macroscopic soft scaffold of **PATA$_L$** are 1.0 cm and 0.5 cm for actuation experiments in water and air, respectively.

Nile Red Fluorescence Assay.[1] The self-assembly of **PATA$_L$** was assessed by incorporation of the hydrophobic solvatochromic probe Nile Red (9-diethylamino-5-benzo[α]phenoxazinone), which exhibits a blue shift emission upon inclusion in hydrophobic environments. Nile Red dissolved at 1 mM in ethanol was diluted into solutions, with a final concentration of 250 nM. As a result, all samples contain 0.25% ethanol, which is expected to not interfere with the self-assembly behavior. Using an Agilent G9800AA Cary Eclipse fluorescence spectrophotometer, the aqueous solutions of **PATA$_L$** (concentration: 5.0 x 10^{-5} to 1.0 mM) and Nile Red (250 nM) were excited at 550 nm and the spectra were recorded over a wavelength range of 580–750 nm. Blue shifts were calculated by subtracting the emission wavelength of Nile Red in Milli-Q water (λ_{em} = 660 nm) from the emission wavelength of the

sample. Blue shifts were plotted against concentrations to determine a critical aggregation concentration.

Transmission Electron Microscopy (TEM). TEM was performed on a JEOL Model JEM-2010 Transmission Electron Microscope with hair pin type tungsten filament operating at 120 kV equipped with Gatan 794 CCD camera. TEM samples were prepared by depositing sample solutions (5.0 μL) onto a carbon grid (Micro to Nano, EMR Carbon support film on copper, 400 square mesh) for 20 s. The sample solution was removed by blotting and UranyLess EM stain solution (Electron Microscopy Science, 5.0 μL) was directly deposited onto the grid for 20 s and the stain was removed by blotting.

Scanning Electron Microscopy (SEM) and Polarized Optical Microscopy (POM). SEM was performed on a Tescan VEGA3 Scanning Electron Microscope. Preparation of a string of **PATA*L*** on a glass substrate: An aqueous solution of **PATA*L*** (5.0 wt.%) was manually drawn into an aqueous solution of $CaCl_2$ (150 mM) from a pipette, a string with arbitrary length was formed. After removal of a solution of metal chloride, the string was washed with MilliQ water for three times and was used directly for POM and SEM experiments. A string for SEM was directly prepared on conductive carbon adhesive tape and dried in air for 48 h before measurement. The air-dried samples were subject to gold sputtering for 20 min prior to SEM measurement. POM was performed on a Leica DM2700-P optical polarizing microscope.

Laser Confocal Raman Microscopy. Raman spectra of **PATA*L*** were recorded on a Renishaw Micro-Raman Spectroscopy System with 785 nm laser from 200 to 3400 cm^{-1}.

Wide Angle X-ray Diffraction (WAXD) and Small-angle X-ray scattering (SAXS). WAXD and SAXS of **PATA*L*** sample was measured in a Borosilicate Glass Capillary Tubes (φ = 2.0 cm) using the Rigaku NANOPIX equipped with a HyPix-6000 (Rigaku) detector. The scattering vector ($q = 4\pi\sin\theta/\lambda$), scattering angle θ and the position of the incident X-ray beam on the detectors were calibrated using several orders of layer reflections from silver behenate (d = 58.380 Å), where λ refers to the wavelength of the X-ray beam (Cu $K\alpha$, 1.54 Å). The sample-to-detector distance was ca. 100 mm for WAXD and ca. 729 mm for SAXS. The obtained diffraction patterns were integrated along the Debye-Scherrer ring to afford 1D intensity data using the Rigaku 2DP software.

Atomic Force Microscopy (AFM). The atomic force microscopy was performed on a JPK NanoWizard V BioScience AFM (Bruker) equipped on Nikon ECLIPSE Ti-2 microscope with a cantilever (PFQNM-LC-V2) to measure the young’s modulus of the scaffold. The macroscopic scaffolds of **PATA*L*** were prepared on a confocal dish mounted on a microscopic slide. The measurement was conducted in water using contact mode force spectroscopy. Five replications were performed, and the data was averaged to show the young modulus of the macroscopic scaffold of **PATA*L*** and the corresponding standard deviation.

Cell culture and cell attachment study. C2C12 myoblasts were purchased from ATCC (CRL1772). The cells were expanded using DMEM based medium, Dulbecco's Modified Eagle Medium (Gibco™, thermofisher:11965092) supplemented with 10% Fetal Bovine Serum (Gibco™, thermofisher:10270106) and 1% Antibiotic-Antimycotic (100X) (Gibco™,

thermofisher:15240062), under 37 °C, 5% CO_2 for cell expansion. Human mesenchymal stem cells (hMSCs) were purchased from Lonza (PT-2501). The cells were expanded using MEM α (Gibco™, thermofisher:12571063), supplemented with 10% Fetal Bovine Serum (Gibco™, thermofisher:10270106) and 1% Antibiotic-Antimycotic (Gibco™, thermofisher:15240062). 10 ng/mL fibroblast growth factor (Sino Biological: 10014-HNAE) was supplemented during hMSCs expansion.

To minimize the cell adhesion on the background culture substrate, we employed bio-inert cell culture dish for our cell-material interface study. For hMSCs *in vitro* experiment, **PATA*L*** was prepared at 3.0 wt.% and heated at 80 °C for 10 mins. After that, the **PATA*L*** solution was injected into MEM α based medium, supplemented with 10% Fetal bovine serum and 1% Antibiotic-Antimycotic to form macroscopic scaffolds in bio-inert culture dish. hMSCs was prepared in suspension at density of 10^5 cells/mL. We added the live cell actin probe, SPY555-FastAct™, to the cell suspension at 1 μM concentraion. After that, we seeded 2×10^5 hMSCs in **PATA*L*** macroscopic scaffolds containing bio-inert dish. The concentration of SPY555-FastAct™ after cell seeding is around 0.8 μM. After 5 hours cell culture, we imaged the sample using Leica DM2700 P, equipped with 530 nm light source (Thorlabs) and Y3 filter cube (Leica).

For C2C12 myoblasts *in vitro* experiment, **PATA*L*** was prepared at 3.0 wt.% and heated at 80°C for 10 mins. After that, the **PATA*L*** solution was injected into DMEM based medium, supplemented with 10% Fetal bovine serum and 1% Antibiotic-Antimycotic to form macroscopic scaffolds in bio-inert culture dish. C2C12 myoblasts was prepared in suspension at density of 5×10^4 cells/mL. We seed 10^5 C2C12 myoblasts in **PATA*L*** macroscopic scaffolds containing bio-inert dish. After 4 hours cell culture, C2C12 myoblasts were labelled by CellMask™ Deep Red Actin. The imaging was performed using Leica TCS SPE Confocal Microscope, excitation: 635 nm, emission: 650–700 nm.

Live cell staining and photo-actuation of cell adhered PATA soft scaffold. 2×10^5 hMSCs were cultured with 3.0 wt.% **PATA*L*** scaffold using bioinert culture dish. After 2 hours incubation, the samples were stained by either 2 μM Calcein AM (Invitrogen™, thermofisher C3100MP) or 3 μg/mL CellTracker™ Orange CMRA Dye (Invitrogen™, thermofisher C34551) for live cell observation or photo-actuation experiment respectively. Live cell sample was imaged using Leica DM2700 P, equipped with 470 nm light source (Thorlabs) and I3 filter cube (Leica). Photo-actuation of cell adhered PATA soft scaffold was induced by 450 nm laser light source, and was recorded using Leica DM2700 P, equipped with 530 nm light source (Thorlabs) and Y3 filter cube (Leica).

Microbead encapsulation in PATA soft scaffold. 3.5 wt.% annealed **PATA*L*** solution was mixed with FluoSpheres™ Polystyrene Microspheres (Invitrogen™, thermofisher: F8836) at 6:1 volume ratio to achieve 3.0 wt.% as final concentration of **PATA*L***. The mixture solution was injected into αMEM medium, supplemented with 10% Fetal bovine serum and 1% Antibiotic-Antimycotic to form a macroscopic scaffold. Photo-actuation of microbead encapsulating PATA soft scaffold was induced by 430 nm light source (Thorlabs), and was recorded using Leica DM2700 P, equipped with 470 nm light source (Thorlabs) and I3 filter cube (Leica).

Description of supplementary movies

Irradiation studies were performed with Thorlabs model M430F1 LED (500 mA), M530F2 LED (1000 mA) or blue-light laser (450 nm). The movies are recorded directly with an iPhone Camera or Lumenera INFINITY3-6URC 6.0 Megapixel USB 3 Microscopy Camera for actuation studies under Leica DM2700-P optical polarizing microscope.

Movie S1: Photoactuation of macroscopic soft scaffold of PATA*L* (5.0 wt.%) in $CaCl_2$ (150 mM) after irradiation with 430 nm blue-light source from left side. When an aqueous solution of **PATA*L*** (5.0 wt.%) was manually drawn into an aqueous solution of $CaCl_2$ (150 mM) from a pipette, a noodle-like scaffold with an arbitrary length was formed. Upon photo-irradiation (λ = 430 nm), the soft scaffold of **PATA*L*** bent toward light from an initial angle of 0° to a flexion angle of 54° after 120 s.

Movie S2a: Photoactuation of macroscopic soft scaffold of PATA*L* (5.0 wt.%) in $CaCl_2$ (150 mM) after irradiation with 430 nm blue-light source from both sides. A freshly prepared soft scaffold of **PATA*L*** (5.0 wt.%) was placed in a cuvette and two identical light sources were placed at both sides of the scaffold with the same distance (1.0 cm). Upon irradiation (λ = 430 nm) at the same time, the scaffold showed linear contraction.

Movie S2b: Photoactuation of a microbead (FluoSpheres™ Polystyrene Microspheres, Invitrogen™) encapsulated macroscopic soft scaffold of PATA*L* (3.0 wt.%) after irradiation with 430 nm blue-light source. A freshly prepared microbead encapsulated soft scaffold of **PATA*L*** (3.0 wt.%) was placed in a dish. Upon photo-irradiation (λ = 430 nm, distance = 5.0 cm), the microbead encapsulated soft scaffold of **PATA*L*** bent toward light source.

Movie S3: Photoactuation of macroscopic soft scaffold of PATA*L* (3.0 wt.%) in $CaCl_2$ (150 mM) after irradiation with 430 nm blue-light source from left side. When an aqueous solution of **PATA*L*** (3.0 wt.%) was manually drawn into an aqueous solution of $CaCl_2$ (150 mM) from a pipette, a noodle-like scaffold with an arbitrary length was formed. Upon photo-irradiation (λ = 430 nm), the soft scaffold of **PATA*L*** bent toward light from an initial angle of 0° to a flexion angle of 79° after 60 s.

Movie S4: Photoactuation of macroscopic soft scaffold of PATA*L* (1.0 wt.%) in $CaCl_2$ (150 mM) after irradiation with 430 nm blue-light source from left side. When an aqueous solution of **PATA*L*** (1.0 wt.%) was manually drawn into an aqueous solution of $CaCl_2$ (150 mM) from a pipette, a noodle-like scaffold with an arbitrary length was formed. Upon photo-irradiation (λ = 430 nm), the soft scaffold of **PATA*L*** bent toward light from an initial angle of 0° to a flexion angle of 90° after 6 s.

Movie S5: Photoactuation of macroscopic soft scaffold of PATA*L* (3.0 wt.%) in $CaCl_2$ (150 mM) after irradiation with 430 nm blue-light source from left and right sides sequentially. A freshly prepared soft scaffold of **PATA*L*** (3.0 wt.%) was placed in a cuvette and two identical light sources were placed at both sides of the scaffold with the same distance (1.0 cm). Upon irradiation (λ = 430 nm) at a sequential manner, the soft scaffold of **PATA*L*** bent towards to left side and bent backward to the right side with response to direction of irradiation.

Movie S6: Photoactuation of macroscopic soft scaffold of PATA*L* (3.0 wt.%) in $CaCl_2$ (150 mM) after multiple point irradiation with optical-fiber coupled 430 nm blue-light source. A freshly prepared soft scaffold of **PATA*L*** (3.0 wt.%) was placed in a cuvette and an optical

fiber (Ø400 μm, 0.22 NA) was coupled with light source Upon irradiation (λ = 430 nm) alternatively from various positions, the soft scaffold of **PATA*L*** resulted in a zigzag conformation.

Movie S7: Photoactuation of macroscopic soft scaffold of PATA*L* (3.0 wt.%) in air. When an aqueous solution of **PATA*L*** (3.0 wt.%) was manually drawn into solution of $CaCl_2$ (150 mM) from a pipette, a noodle-like scaffold with an arbitrary length was formed. The scaffold was pulled out of water carefully and suspended on a sample holder. Upon irradiation (λ = 430 nm) for 30 s, the scaffold bent by 90° toward the light source.

Movie S8: Photoactuation of macroscopic soft scaffold of PATA*L* (3.0 wt.%) in air with 0.2 mg paper. A scaffold was prepared by the same method in Movie S7, and a piece of 0.2 mg paper was adhered onto the end of a scaffold. Upon photoirradiation, the scaffold was capable of bending by 30° toward the light source.

Movie S9: Photoactuation of macroscopic soft scaffold of PATA*L* (3.0 wt.%) in $CaCl_2$ (150 mM) to grasp and release a 0.2 mg paper. A scaffold was prepared by manually draw an aqueous solution of **PATA*L*** (3.0 wt.%) into solution of $CaCl_2$ (150 mM) with a pipette, a noodle-like scaffold with an arbitrary length was formed. A piece of 0.2 mg paper was placed near the scaffold. Upon photoirradiation at different sides, the scaffold was capable of grasping and releasing the paper.

Movie S10: Photoactuation of macroscopic soft scaffold of PATA*D* (3.0 wt.%) in $CaCl_2$ (150 mM) after irradiation with 430 nm blue-light source from left side. When an aqueous solution of **PATA*D*** (3.0 wt.%) was manually drawn into an aqueous solution of $CaCl_2$ (150 mM) from a pipette, a noodle-like scaffold with an arbitrary length was formed. Upon photo-irradiation (λ = 430 nm), the soft scaffold of **PATA*L*** bent toward light from an initial angle of 0° to a flexion angle of 77° after 60 s.

Movie S11: Photoactuation of hMSCs (CellTracker™ Orange CMRA Dye labelled) adhered macroscopic soft scaffold of PATA*L* (3.0 wt.%) after irradiation with blue-light laser from left side. A freshly prepared hMSCs adhered macroscopic soft scaffold of **PATA*L*** was placed in a dish. Upon photo-irradiation (λ = 450 nm, laser source), the hMSCs adhered soft scaffold of **PATA*L*** bent toward light source.

Synthesis

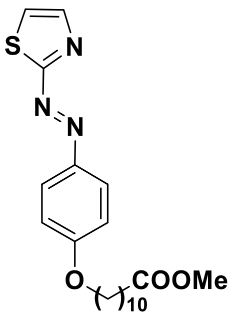

Compound **2**

To a dimethylformamide solution (20 mL) of compound **1**[2] (1 g, 4.87 mmol), methyl 11-bromoundecanoate (2.04 g, 7.31 mmol) and potassium carbonate (1.35 mg, 9.74 mol) was added under nitrogen and the mixture was heated at 80 °C for overnight. The mixture was washed with brine (1x), water (2x) and brine (1x). The combined organic layers were dried over sodium sulphate. The solvent was evaporated under vacuum and then the obtained residue was subjected to column chromatography on silica gel (n-hexane/ethyl acetate; v/v = 3/1, R_f = 0.3) to allow isolation of the compound **2** as a yellow solid (1.21 g, 3.01 mmol) in 62% yield.

^{1}H NMR (600 MHz, $CDCl_3$) δ 8.05 – 7.83 (m, 3H), 7.32 (d, J = 3.3 Hz, 1H), 6.96 (d, J = 8.8 Hz, 2H), 4.00 (t, J = 6.5 Hz, 2H), 3.63 (s, 3H), 2.26 (t, J = 7.6 Hz, 2H), 1.77 (p, J = 6.8 Hz, 2H), 1.58 (t, J = 7.3 Hz, 2H), 1.46 – 1.38 (m, 2H), 1.35 – 1.24 (m, 10H).

^{13}C NMR (151 MHz, $CDCl_3$) δ 177.68, 174.31, 163.39, 145.79, 143.47, 126.20, 120.48, 115.01, 77.34, 77.12, 76.91, 68.52, 51.44, 34.07, 29.44, 29.33, 29.29, 29.21, 29.11, 29.07, 25.94, 24.92.

HRMS (ESI): calcd. for $C_{21}H_{29}N_3O_3S$ [M+H] 404.2002, found 404.2005.

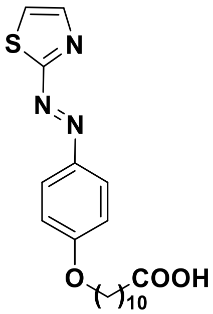

Compound **3**

To a mixture solution of THF (5 mL) and water (5 mL), compound **2** (500 mg, 1.24 mmol) and lithium hydroxide (89.1 mg, 3.72 mmol) were added. The mixture was stirred at 25 °C overnight. Ethyl acetate (25 mL) and water (25 mL) were added to the mixture. The mixture was acidified by addition of HCl until pH reach 2. The resulting yellow precipitate was filtrated, washed with water to afford compound **3** as yellow solid (401 mg, 1.03 mmol) in 83% yield without further purification.

HRMS (ESI): calcd. for $C_{20}H_{27}N_3O_3S$ [M−H] 388.17, found 388.1702.

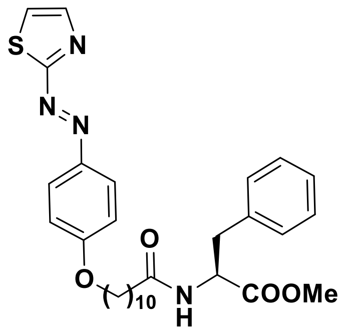

Compound **4**

To a dimethylformamide solution (15 mL) of compound **3** (250 mg, 0.64 mmol), *L*-phenylalanine methyl ester (208 mg, 0.96 mmol), Hexafluorophosphate Benzotriazole Tetramethyl Uronium (HBTU, 487 mg, 1.28 mmol), Hydroxybenzotriazole monohydrate (HoBT • H2O, 174 mg, 1.28 mmol) were added under nitrogen. After dropwise addition of *N,N'*-diisopropylethylamine (DIPEA, 0.6 mL, 3.4 mmol), the reaction mixture was stirred at 25 °C for 48 h. The mixture was washed with brine (25 mL x 1), water (25 mL x 2) and brine (25 mL x 1). The combined organic layers were dried over sodium sulphate. The solvent was evaporated under vacuum and then the obtained residue was subjected to column chromatography on silica gel (dichloromethane/ethyl acetate; v/v = 19/1, R_f = 0.4) to allow isolation of the compound **4** as a yellow solid (282 mg, 0.51 mmol) in 82% yield.

^{1}H NMR (600 MHz, $CDCl_3$) δ 7.99 (d, *J* = 8.8 Hz, 2H), 7.35 (d, *J* = 3.4 Hz, 1H), 7.27 (t, *J* = 7.4 Hz, 2H), 7.24 (d, *J* = 7.2 Hz, 1H), 7.10 (d, *J* = 7.3 Hz, 2H), 7.00 (d, *J* = 8.6 Hz, 2H), 6.08 (d, *J* = 7.9 Hz, 1H), 4.90 (d, *J* = 7.2 Hz, 1H), 4.03 (t, *J* = 6.5 Hz, 2H), 3.72 (s, 3H), 3.15 (dd, *J* = 13.9, 5.8 Hz, 1H), 3.08 (dd, *J* = 13.9, 6.1 Hz, 1H), 2.21 – 2.13 (m, 2H), 1.80 (t, *J* = 7.4 Hz, 2H), 1.58 (t, *J* = 7.2 Hz, 2H), 1.45 (t, *J* = 7.7 Hz, 2H), 1.34 (q, *J* = 7.2 Hz, 2H), 1.32 – 1.24 (m, 10H).

^{13}C NMR (151 MHz, $CDCl_3$) δ 177.71, 172.75, 172.23, 163.40, 145.82, 143.52, 135.98, 129.25, 128.54, 127.08, 126.22, 120.55, 115.04, 68.54, 52.95, 52.30, 37.89, 36.48, 29.48, 29.36, 29.32, 29.30, 29.17, 29.09, 25.96, 25.54.

HRMS (ESI): calcd. for $C_{30}H_{38}N_4O_4S$ [M+H] 551.2687, found 551.2689.

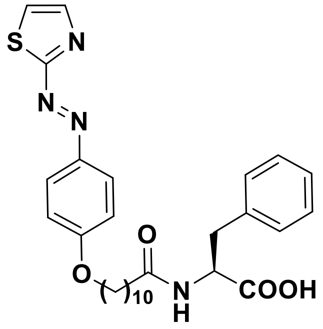

PATA*L*

To a mixture solution of THF (5 mL) and water (5 mL), compound **4** (300 mg, 0.54 mmol) and lithium hydroxide (65.2 mg, 2.72 mmol) were added. The mixture was stirred at 25 °C overnight. Ethyl acetate (25 mL) and water (25 mL) were added to the mixture. The mixture was acidified by addition of HCl until pH reach 2. The resulting yellow precipitate was filtrated, washed with water to afford compound **6** as yellow solid (251 mg, 0.47 mmol) in 85% yield.

^{1}H NMR (600 MHz, MeOD) δ 8.01 – 7.95 (m, 3H), 7.66 (d, *J* = 3.3 Hz, 1H), 7.29 – 7.17 (m, 5H), 7.14 – 7.09 (m, 2H), 4.67 (dd, *J* = 9.7, 4.8 Hz, 1H), 4.12 (t, *J* = 6.5 Hz, 2H), 3.22 (dd, *J* = 14.1, 4.9 Hz, 1H), 2.92 (dd, *J* = 14.0, 9.7 Hz, 1H), 2.14 (t, 2H), 1.90 – 1.77 (m, 2H), 1.55 – 1.45 (m, 4H), 1.39 (t, *J* = 7.8 Hz, 2H), 1.35 – 1.11 (m, 8H).

^{13}C NMR (151 MHz, $CDCl_3$/MeOD) δ 177.85, 173.62, 173.45, 163.52, 145.74, 143.07, 136.18, 129.30, 128.37, 126.90, 126.21, 120.62, 115.04, 77.34, 77.12, 76.91, 68.55, 52.90, 37.35, 36.32, 29.41, 29.29, 29.24, 29.21, 29.08, 29.01, 25.88, 25.51.
HRMS (ESI): calcd. for $C_{29}H_{36}N_4O_4S$ [M−H] 535.2382, found 535.2381.

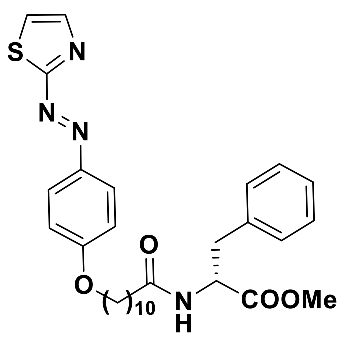

Compound **5**

To a dimethylformamide solution (15 mL) of compound **3** (250 mg, 0.64 mmol), *D*-phenylalanine methyl ester (208 mg, 0.96 mmol), Hexafluorophosphate Benzotriazole Tetramethyl Uronium (HBTU, 487 mg, 1.28 mmol), Hydroxybenzotriazole monohydrate (HoBT • H2O, 174 mg, 1.28 mmol) were added under nitrogen. After dropwise addition of N,N-diisopropylethylamine (DIPEA, 0.6 mL, 3.4 mmol), the reaction mixture was stirred at 25 °C for 48 h. The mixture was washed with brine (25 mL x 1), water (25 mL x 2) and brine (25 mL x 1). The combined organic layers were dried over sodium sulphate. The solvent was evaporated under vacuum and then the obtained residue was subjected to column chromatography on silica gel (dichloromethane/ethyl acetate; v/v = 19/1, R_f = 0.4) to allow isolation of the compound **5** as a yellow solid (301 mg, 0.55 mmol) in 86% yield.
^1H NMR (600 MHz, $CDCl_3$) δ 8.03 – 7.96 (m, 3H), 7.36 (d, *J* = 3.3 Hz, 1H), 7.31 – 7.21 (m, 3H), 7.09 (d, *J* = 7.0 Hz, 2H), 7.01 (d, *J* = 9.0 Hz, 2H), 5.89 (d, *J* = 7.8 Hz, 1H), 4.91 (dd, 1H), 4.05 (t, *J* = 6.5 Hz, 2H), 3.73 (s, 3H), 3.16 (dd, *J* = 13.9, 5.8 Hz, 1H), 3.09 (dd, *J* = 13.9, 5.8 Hz, 1H), 2.17 (t, *J* = 8.3 Hz, 2H), 1.86 – 1.78 (m, 2H), 1.59 (t, *J* = 7.2 Hz, 2H), 1.40 – 1.30 (m, 2H), 1.32 – 1.24 (m, 8H).
^{13}C NMR (151 MHz, $CDCl_3$) δ 177.73, 172.64, 172.21, 163.42, 145.83, 143.52, 135.91, 129.27, 128.57, 127.13, 126.24, 120.46, 115.03, 68.56, 52.91, 52.33, 37.93, 36.56, 29.49, 29.37, 29.33, 29.31, 29.19, 29.11, 25.97, 25.53.
HRMS (ESI): calcd. for $C_{30}H_{38}N_4O_4S$ [M+H] 551.2687, found 551.2688.

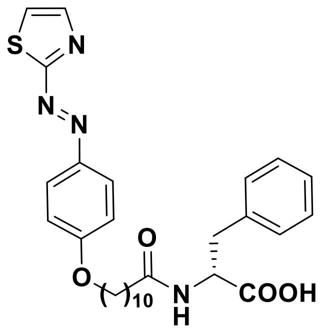

PATA*D*

To a mixture solution of THF (5 mL) and water (5 mL), compound **5** (250 mg, 0.45 mmol) and lithium hydroxide (54.4 mg, 2.27 mmol) were added. The mixture was stirred at 25 °C

overnight. Ethyl acetate (25 mL) and water (25 mL) were added to the mixture. The mixture was acidified by addition of HCl until pH reach 2. The resulting yellow precipitate was filtrated, washed with water to afford compound **7** as yellow solid (209 mg, 0.39 mmol) in 87% yield.
^{1}H NMR (400 MHz, MeOD) δ 8.01 – 7.93 (m, 3H), 7.64 (d, *J* = 3.4 Hz, 1H), 7.22 (d, *J* = 7.1 Hz, 5H), 7.10 (d, *J* = 8.7 Hz, 2H), 4.61 (dd, *J* = 9.0, 4.8 Hz, 1H), 4.10 (t, *J* = 6.4 Hz, 2H), 3.21 (dd, *J* = 13.8, 4.8 Hz, 1H), 2.92 (dd, *J* = 13.8, 9.0 Hz, 1H), 2.13 (t, *J* = 7.4 Hz, 2H), 1.81 (p, *J* = 6.8 Hz, 2H), 1.47 (p, *J* = 7.5 Hz, 4H), 1.42 – 1.27 (m, 10H).
^{13}C NMR (151 MHz, $CDCl_3$/MeOD) δ 177.99, 174.60, 174.36, 163.86, 145.75, 142.65, 137.38, 129.05, 127.95, 126.27, 125.94, 121.05, 115.01, 68.41, 54.17, 37.41, 35.77, 29.28, 29.15, 29.12, 29.09, 28.89, 28.81, 25.75, 25.55.
HRMS (ESI): calcd. for $C_{29}H_{36}N_4O_4S$ [M−H] 535.2382, found 535.2383.

Supporting Figures

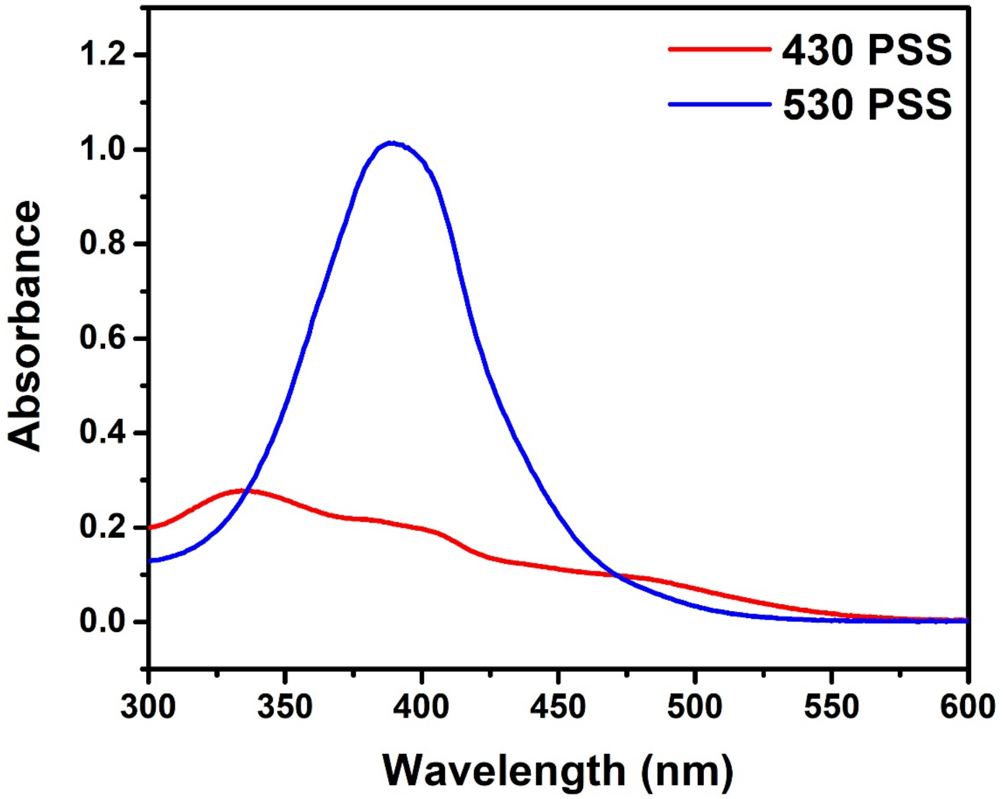

Figure S1. UV-vis absorption spectra of **PATA*L*** (50 μM), *Z*-**PATA*L*** to *E*-**PATA*L*** in MeOH.

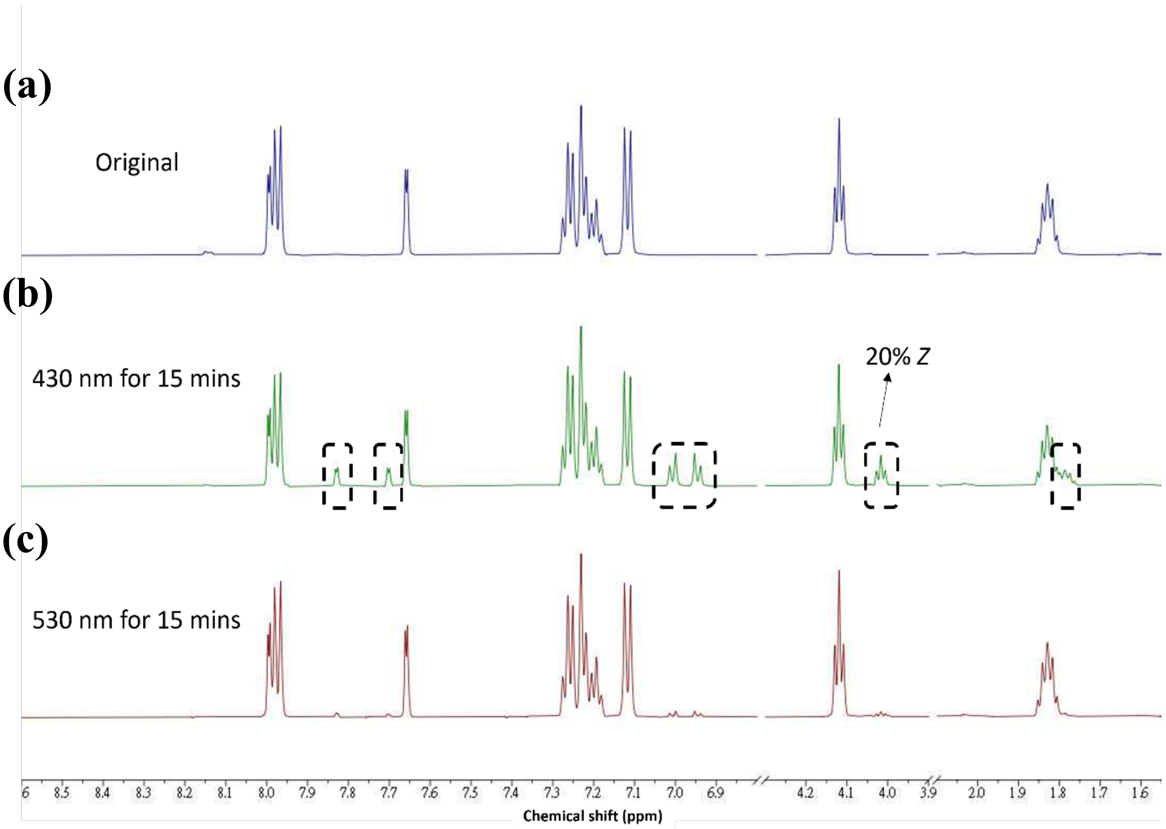

Figure S2. (a) Selected aromatic and aliphatic regions in the ^{1}H NMR spectra of *E*-**PATA*L*** (2.0 mM, MeOD, 25 °C, 600 MHz), (b) was irradiated with 430 nm for 15 min, (b) and consecutively irradiated with 530 min for 10 min.

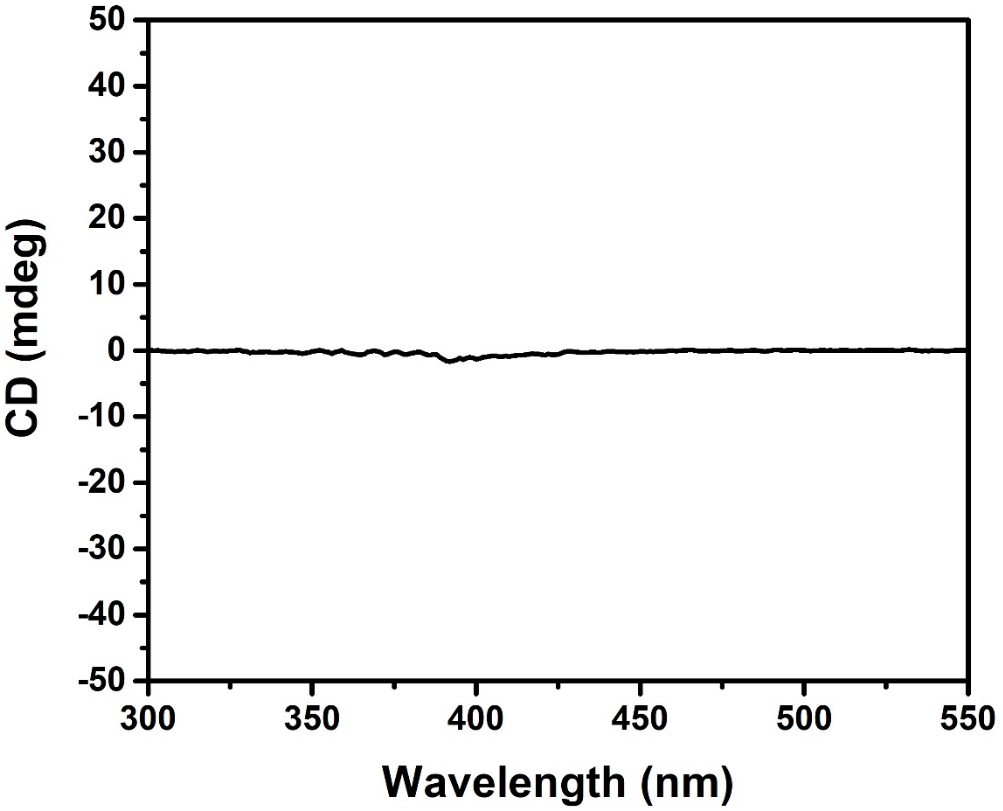

Figure S3. CD spectrum of **PATA**$_L$ (50 μM) in MeOH.

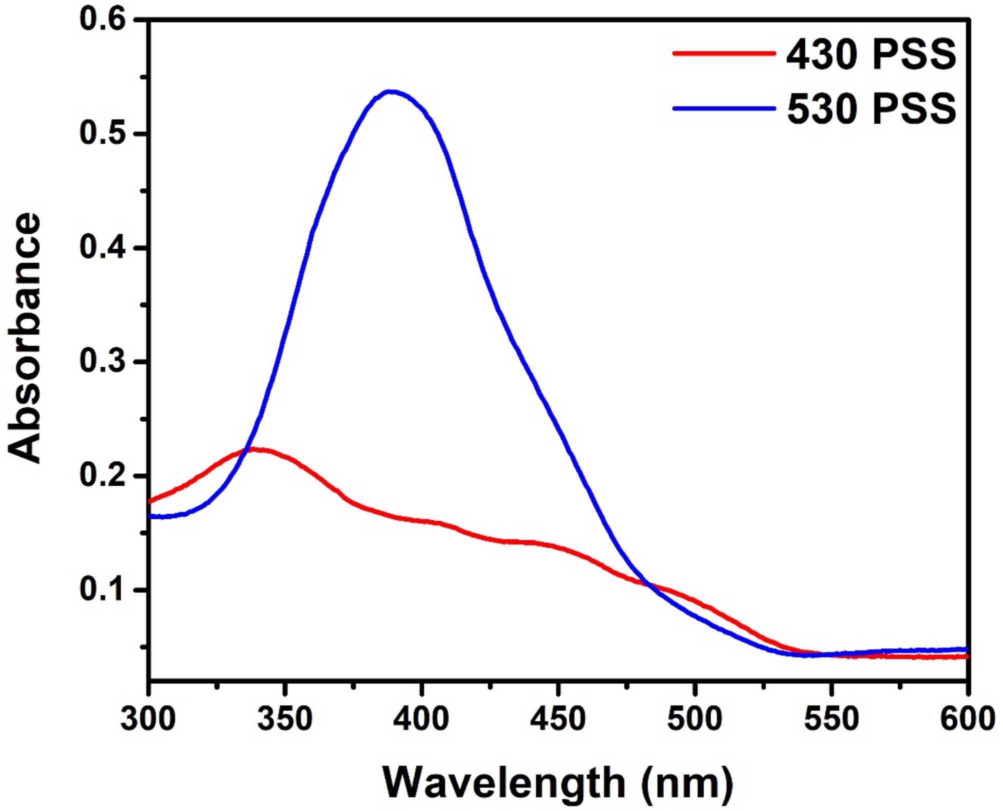

Figure S4. UV-vis absorption spectra of **PATA***L* (50 μM), *Z*-**PATA***L* to *E*-**PATA***L* in water.

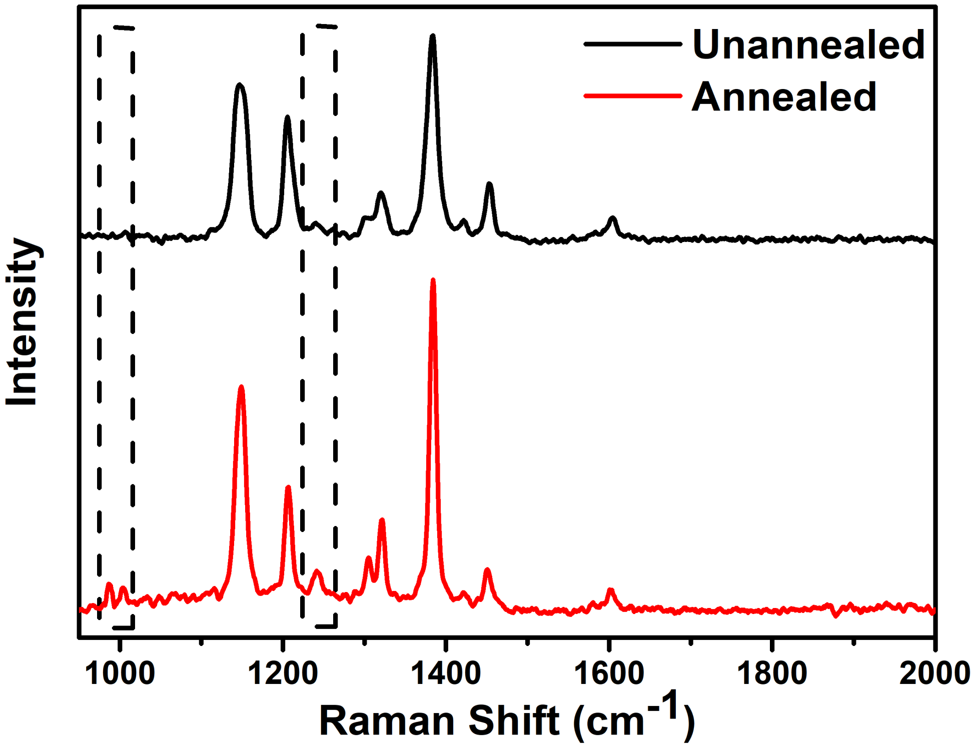

Figure S5. Laser confocal Raman spectra of **PATA*L*** before and after thermal annealing process.

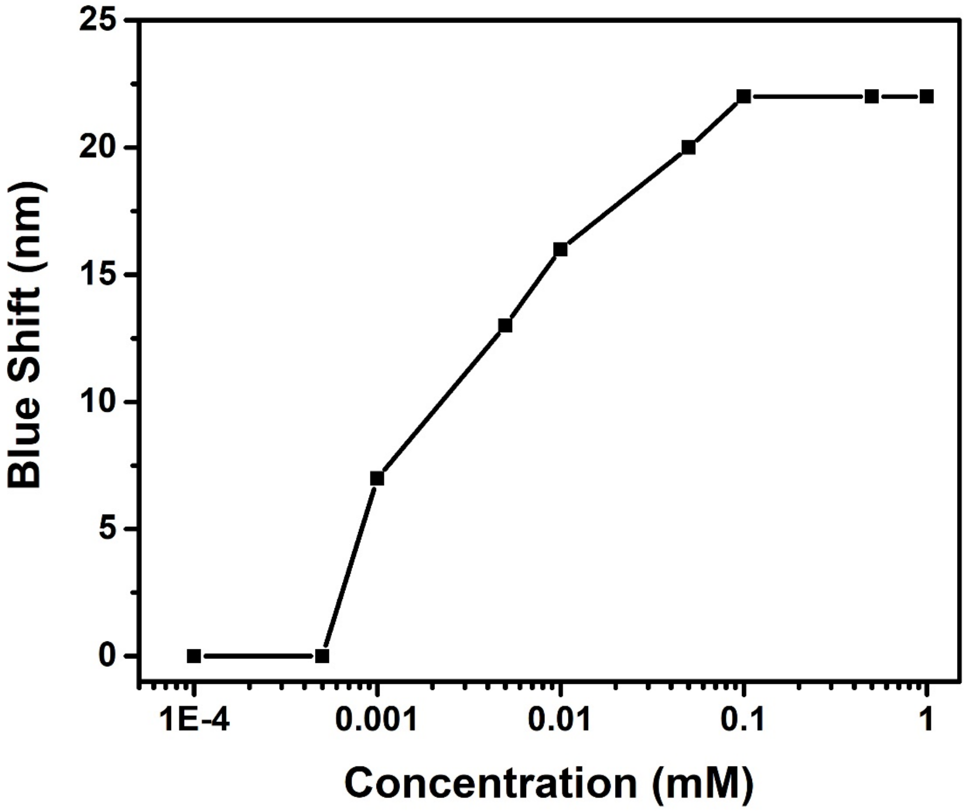

Figure S6. Nile Red fluorescence assay for determination of the critical aggregation concentration of **PATA*L*** (concentration: 5.0 ×10^{-5} to 1.0 mM).

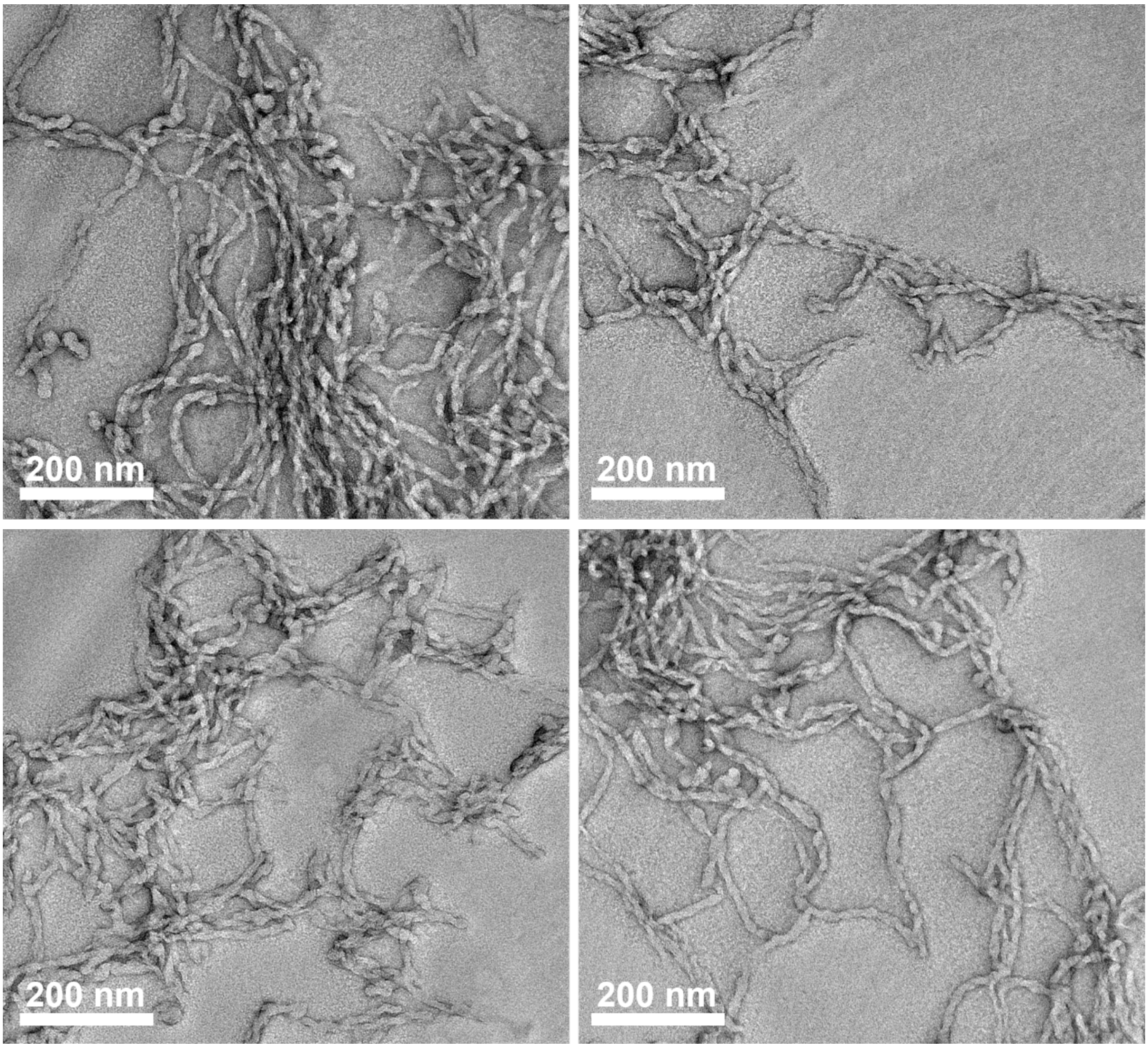

Figure S7. TEM images of an aqueous solution of **PATA$_L$**.

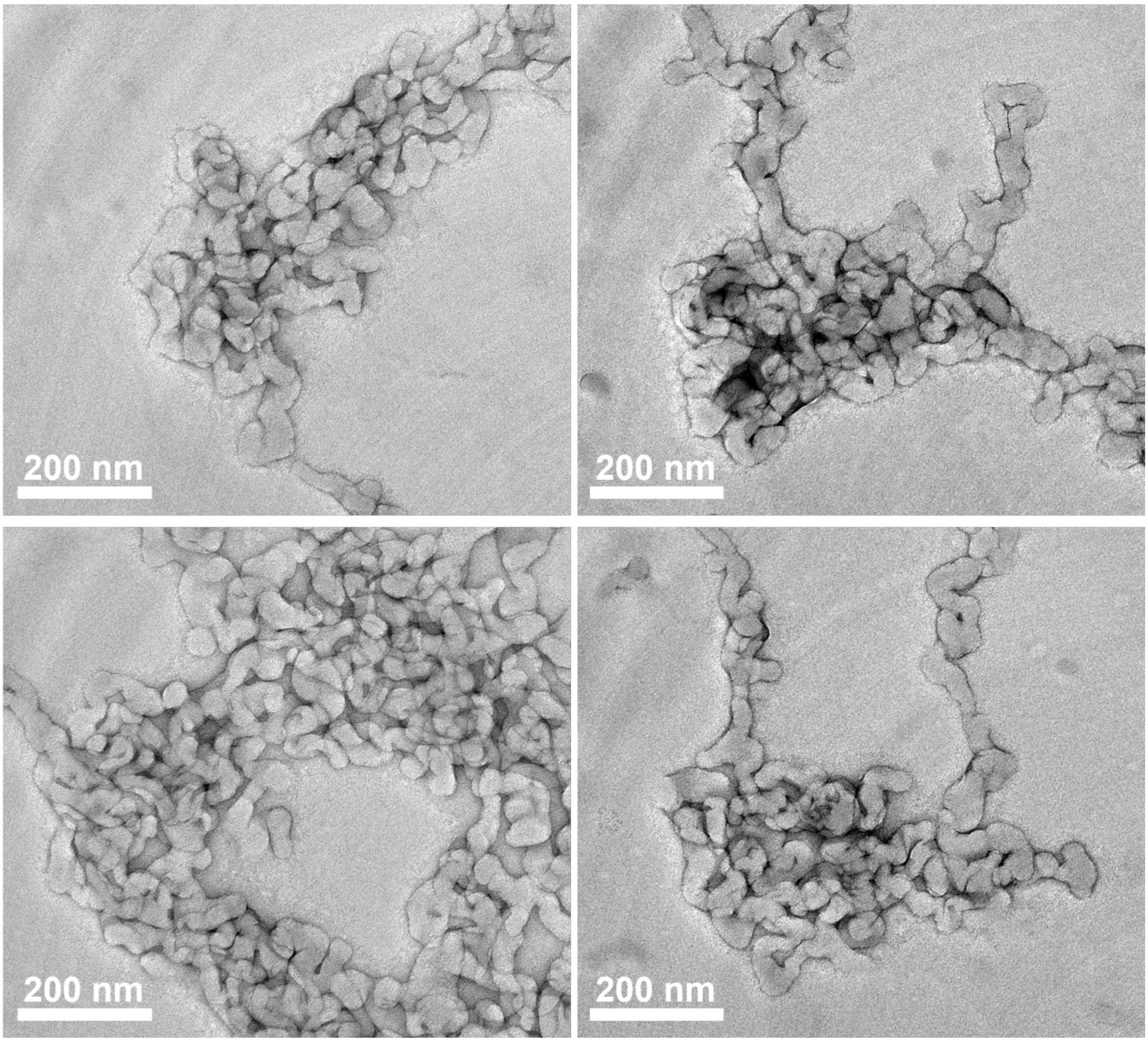

Figure S8. TEM images of an aqueous solution of **PATA**$_L$ after 430 nm blue-light irradiation.

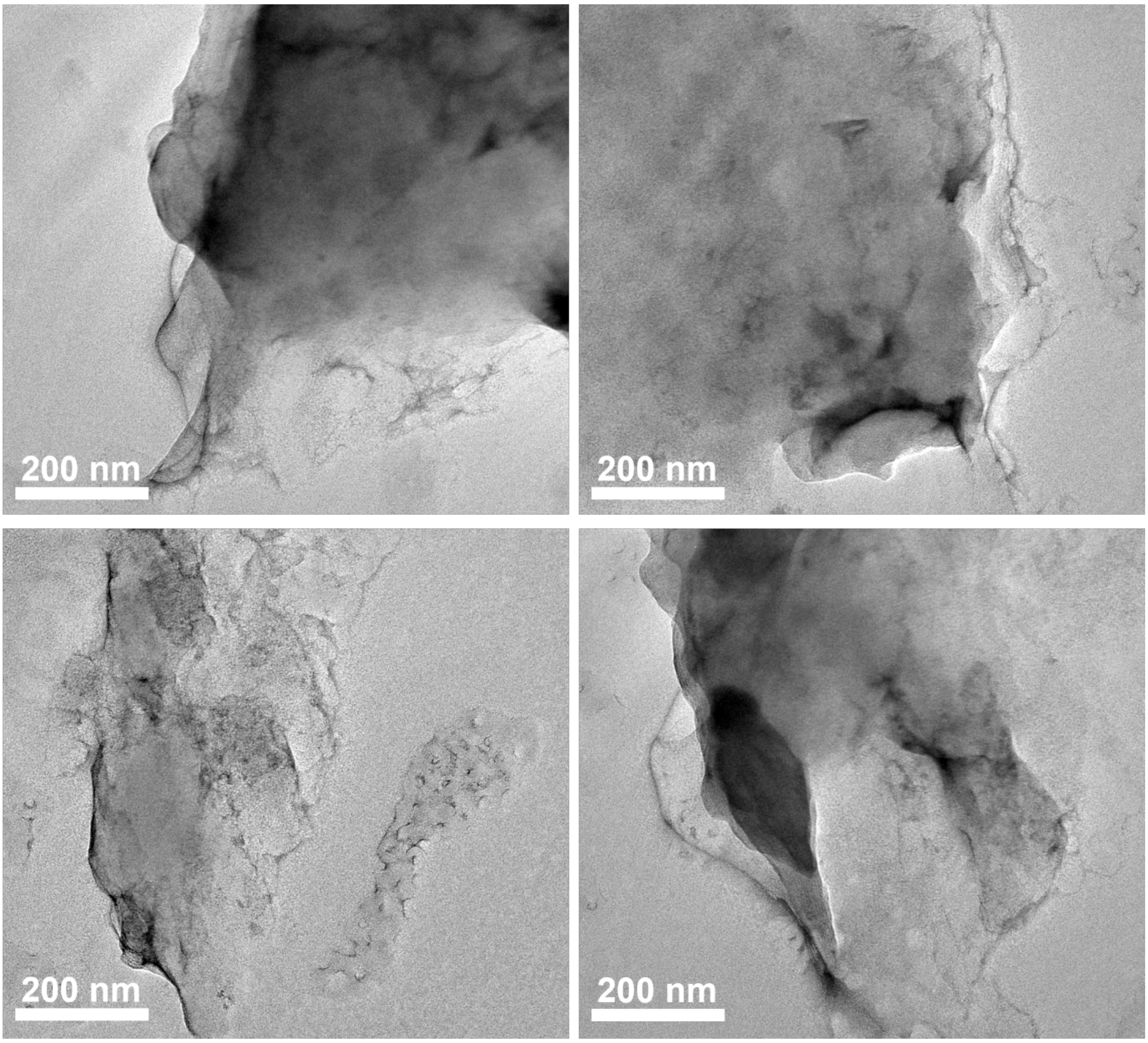

Figure S9. TEM images of an aqueous solution of **PATA$_L$** after 430 nm blue-light irradiation and subsequently followed by 530 nm green-light irradiation.

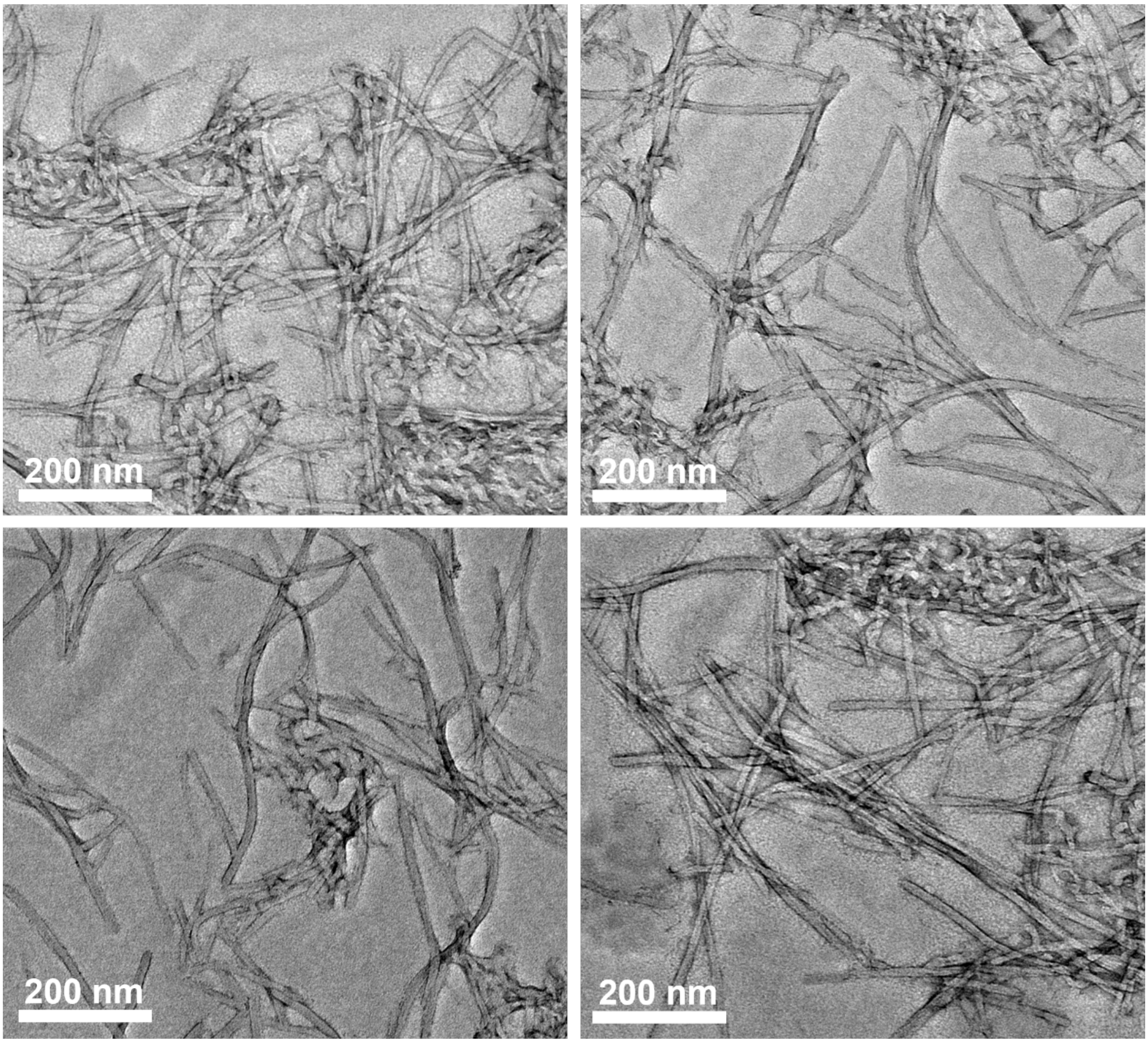

Figure S10. TEM images of an aqueous solution of **PATA**$_{L}$ after 430 nm blue-light irradiation and subsequently followed by 530 nm green-light irradiation with sonication.

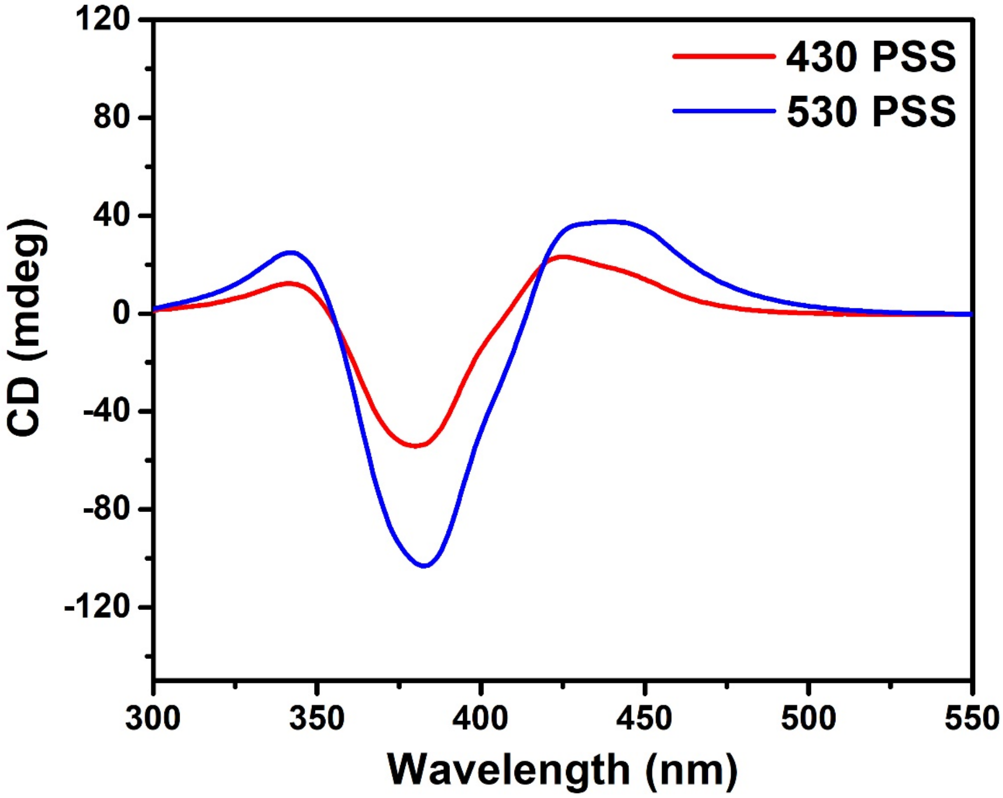

Figure S11. CD spectra of **PATA*L*** (50 μM), *Z*-**PATA*L*** to *E*-**PATA*L*** in water.

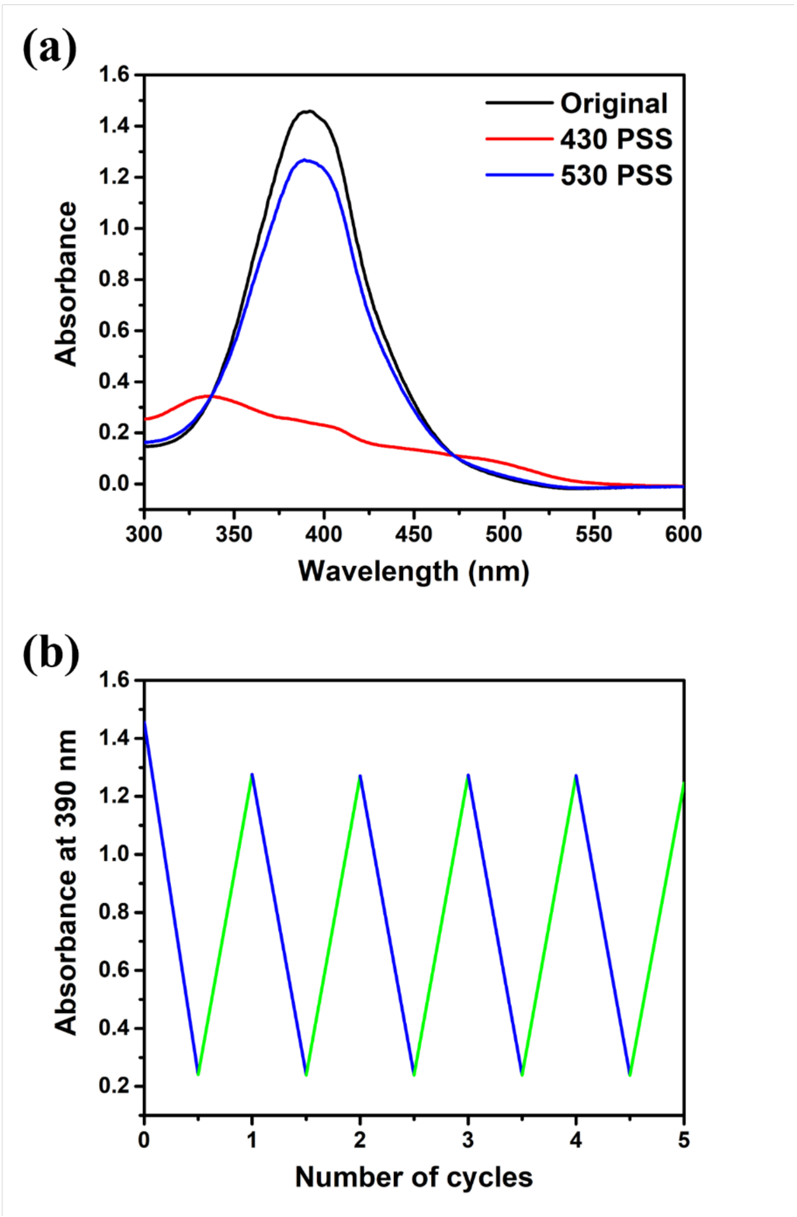

Figure S12. UV-vis absorption spectra of **PATA*D*** (50 μM) MeOH. (b) The change in UV-vis absorption for **PATA*D*** (50 μM) in MeOH after five blue-light irradiations (blue-line) / green-light irradiations (green-line) cycles.

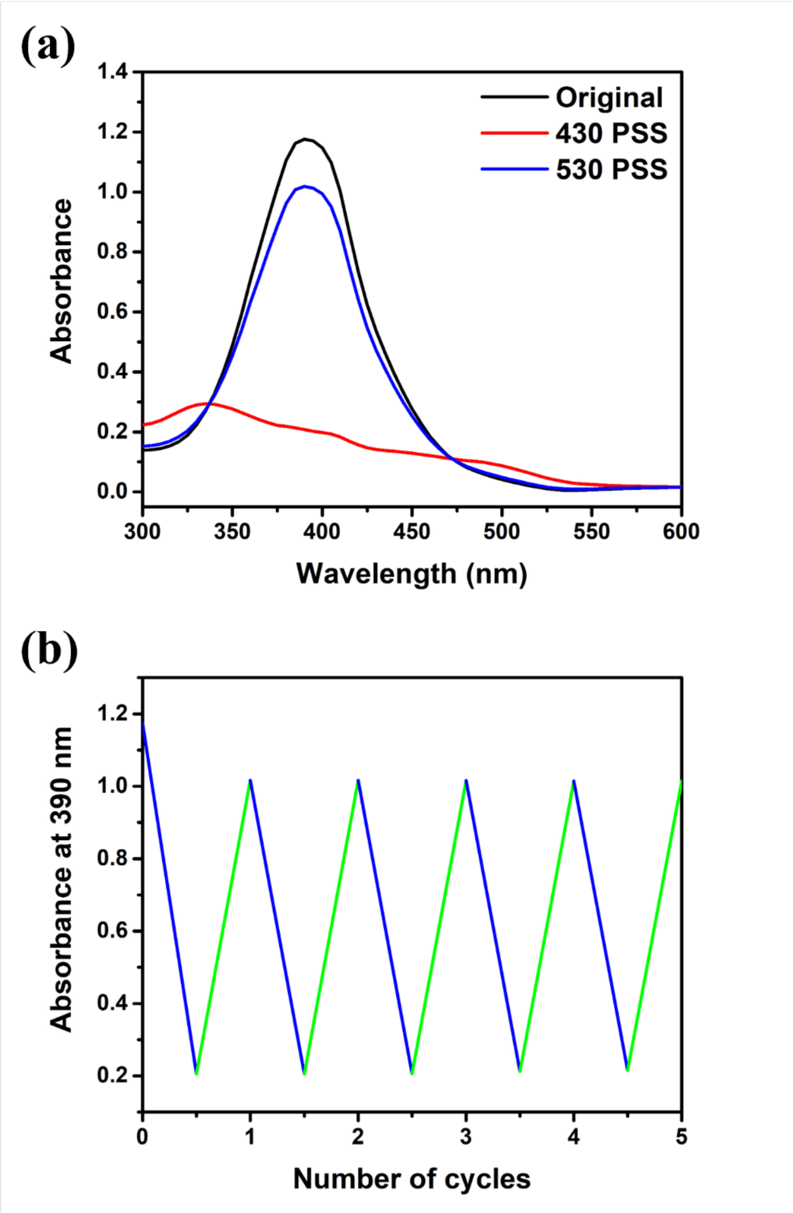

Figure S13. UV-vis absorption spectra of **PATA*D*** (50 μM) water. (b) The change in UV-vis absorption for **PATA*D*** (50 μM) in water after five blue-light irradiations (blue-line) / green-light irradiations (green-line) cycles.

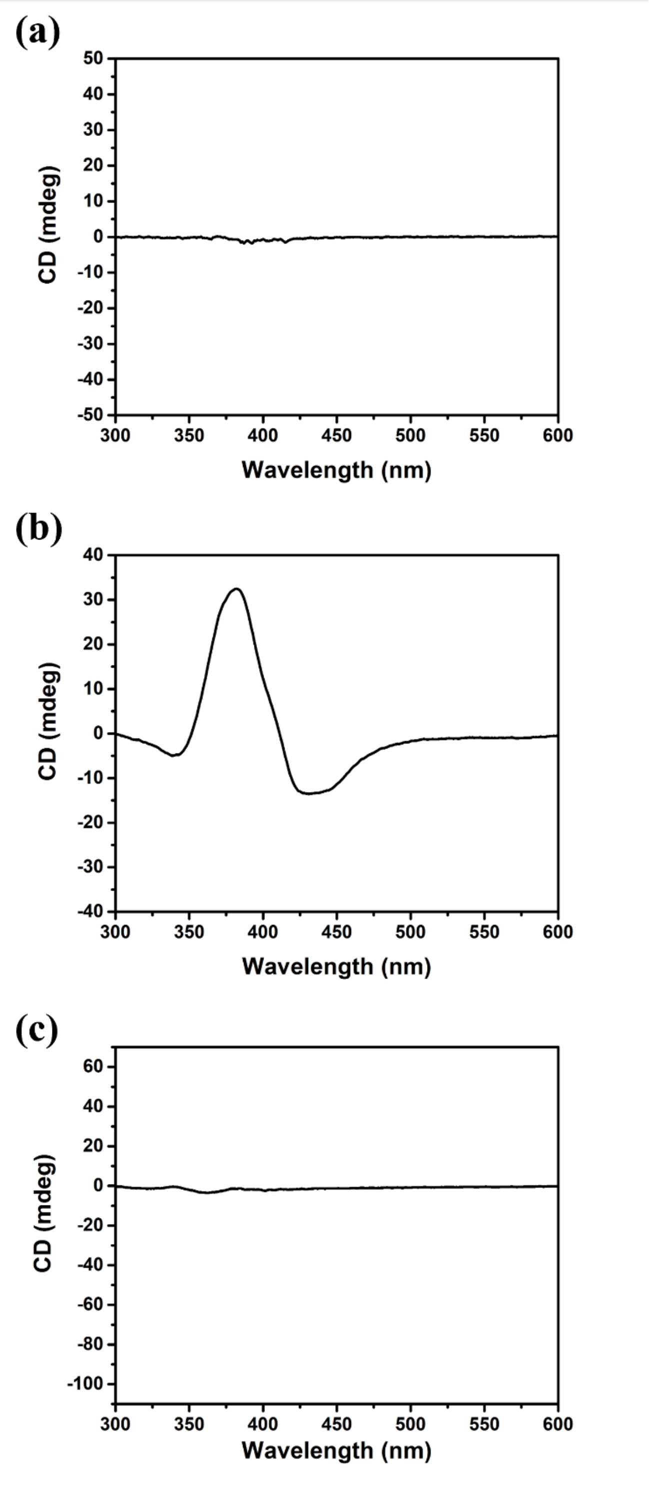

Figure S14. CD spectra of **PATA*D*** (50 μM) in (a) MeOH and (b) water. (c) CD spectrum of racemic mixture of **PATA*L*** and **PATA*D*** (50 μM) in water.

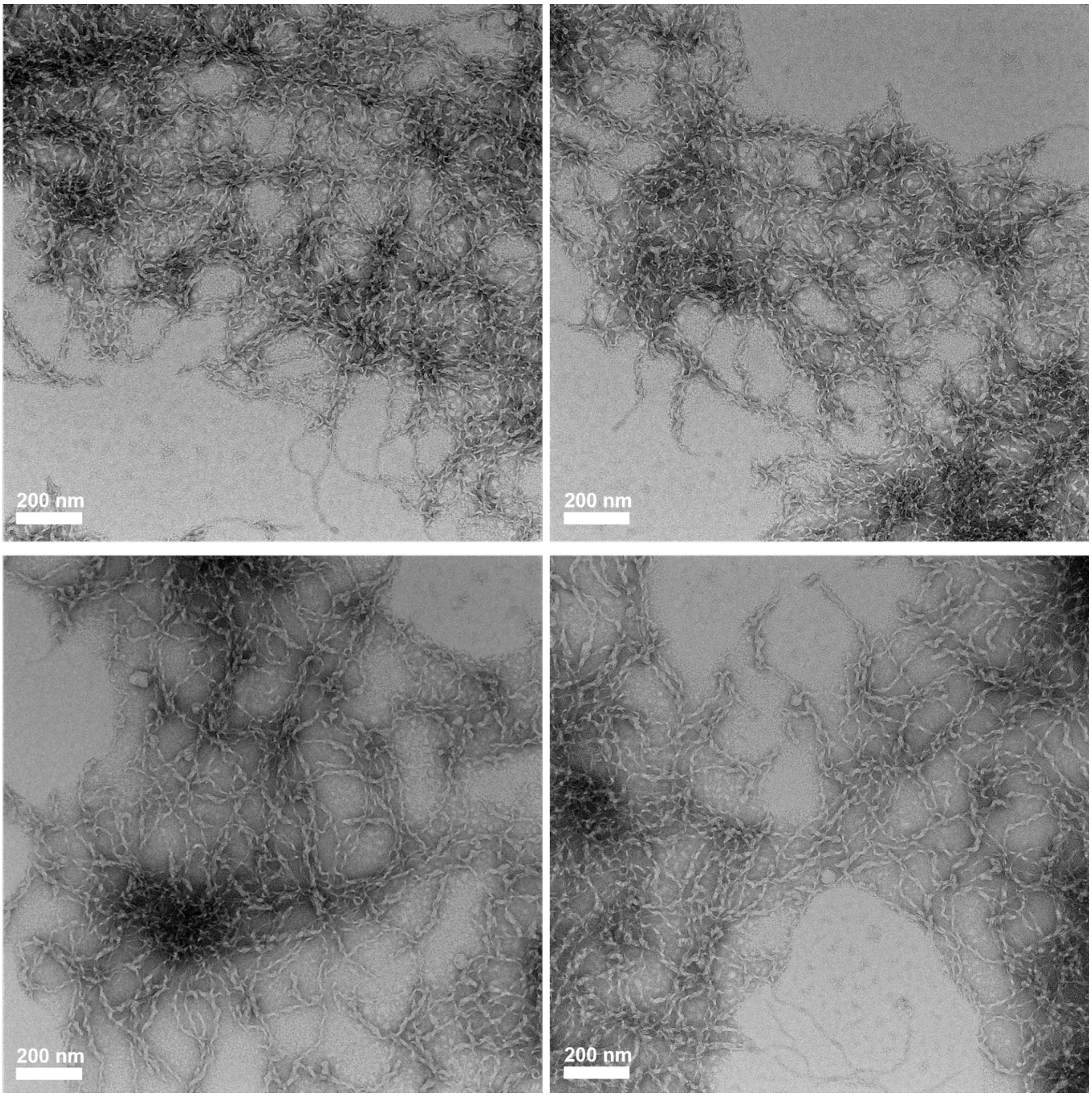

Figure S15. TEM images of an aqueous solution of $\mathbf{PATA}_{D}$.

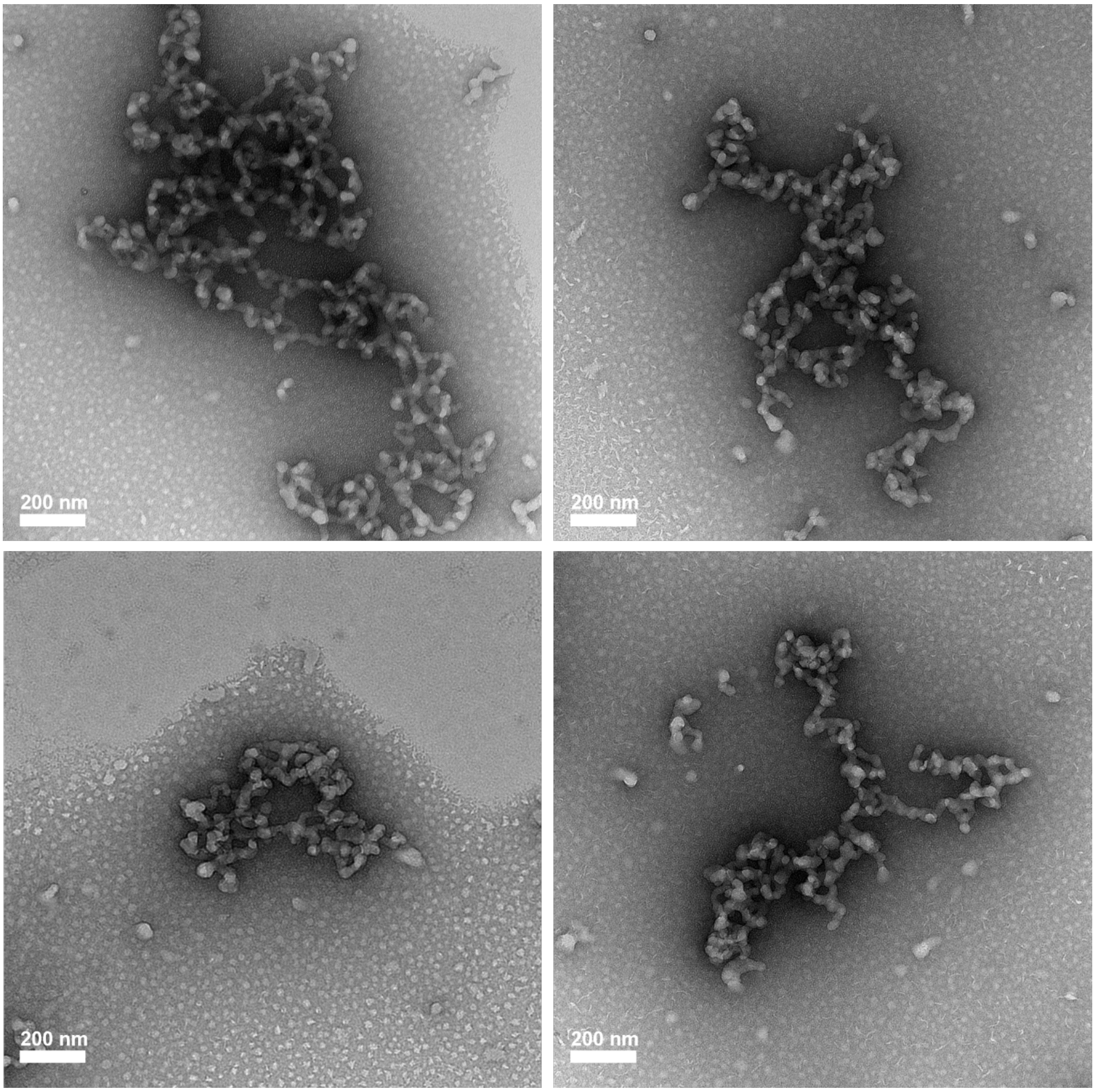

Figure S16. TEM images of an aqueous solution of **PATA**$_{\boldsymbol{D}}$ after 430 nm blue-light irradiation.

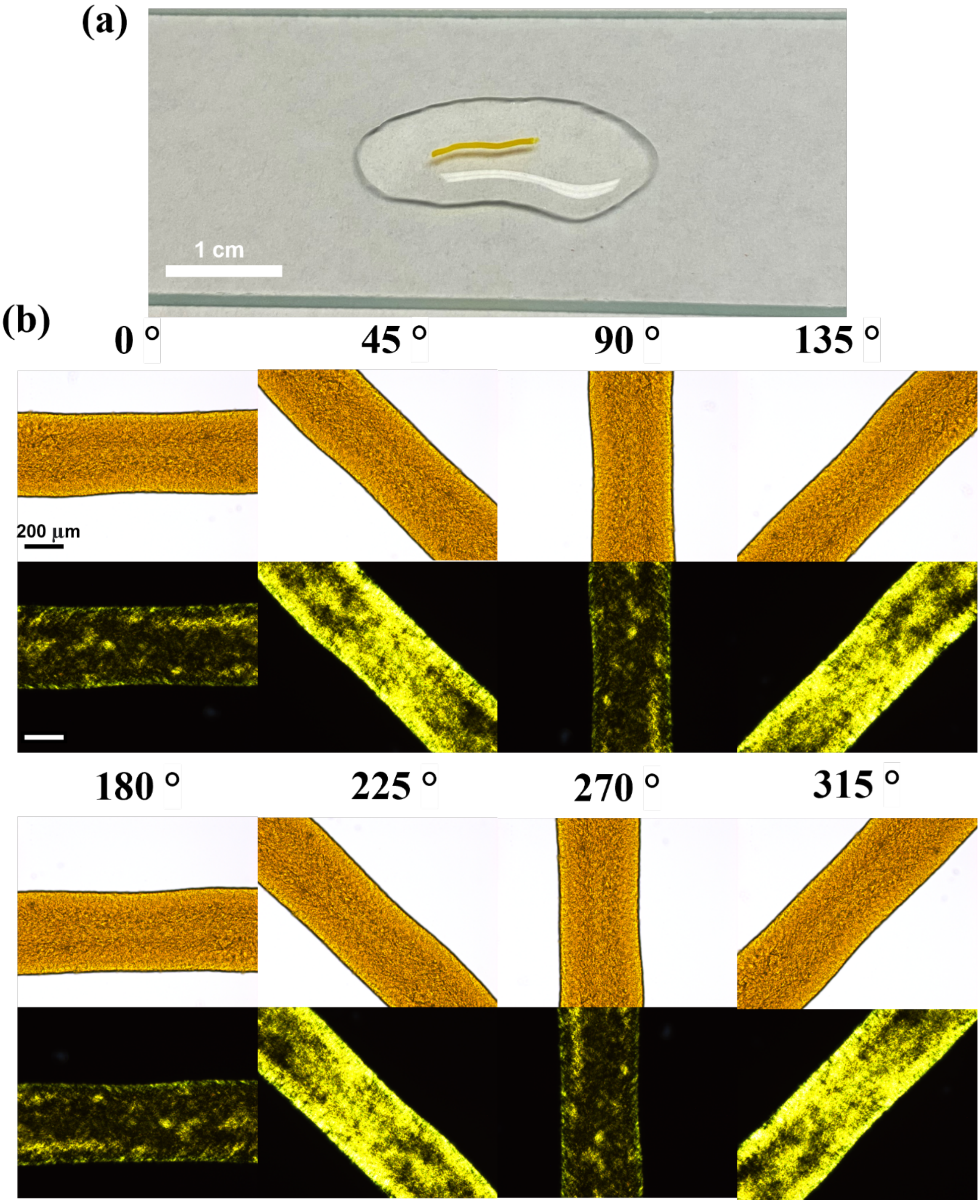

Figure S17. (a) Photograph of a macroscopic soft scaffold of **PATA$_L$** (5.0 wt.%) prepared from an aqueous solution of calcium chloride (150 mM). (b) Optical microscopic images of a macroscopic soft scaffold of **PATA$_L$** (5.0 wt.%) prepared from a solution of $CaCl_2$ (150 mM) under crossed polarizers. The POM and OM images of the soft scaffold were tilted at 0°, 45°, 90°, 135°, 180°, 225°, 270° and 315° relative to the transmission axis of the analyzer. Scale bar 200 μm for all panels.

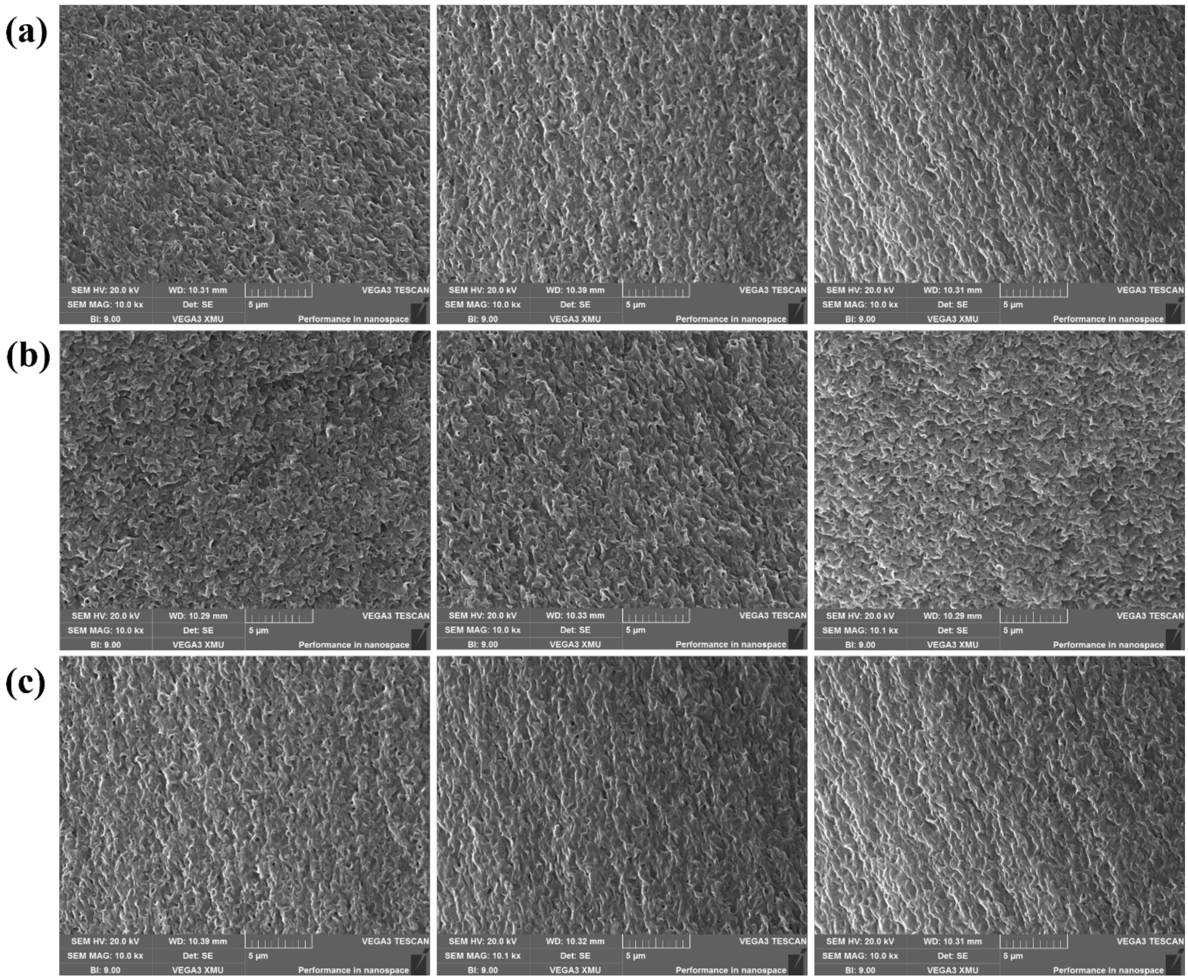

Figure S18. SEM images of an air-dried macroscopic soft scaffold of **PATA*L*** with (a) 5.0 wt.%, (b) 3.0 wt.% and (c) 1.0 wt.%.

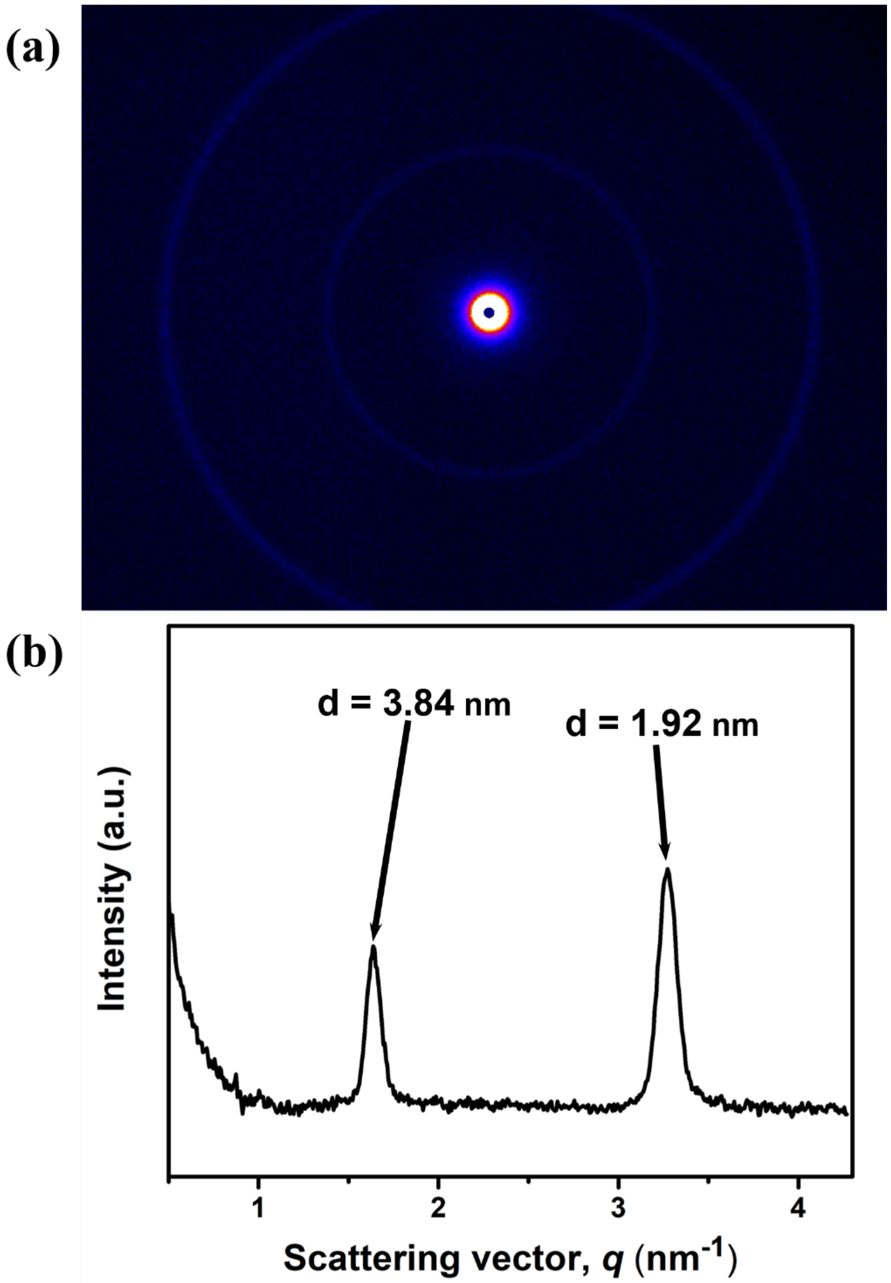

Figure S19. (a) 2D-SAXS image of a macroscopic soft scaffold of **PATA**$_L$ (5.0 wt.%, 93.2 mM). (b) 1D-SAXS pattern of a macroscopic soft scaffold of **PATA**$_L$ of 2D-SAXS image in (a), showing the diffraction pattern in perpendicular to the long axis of the scaffold.

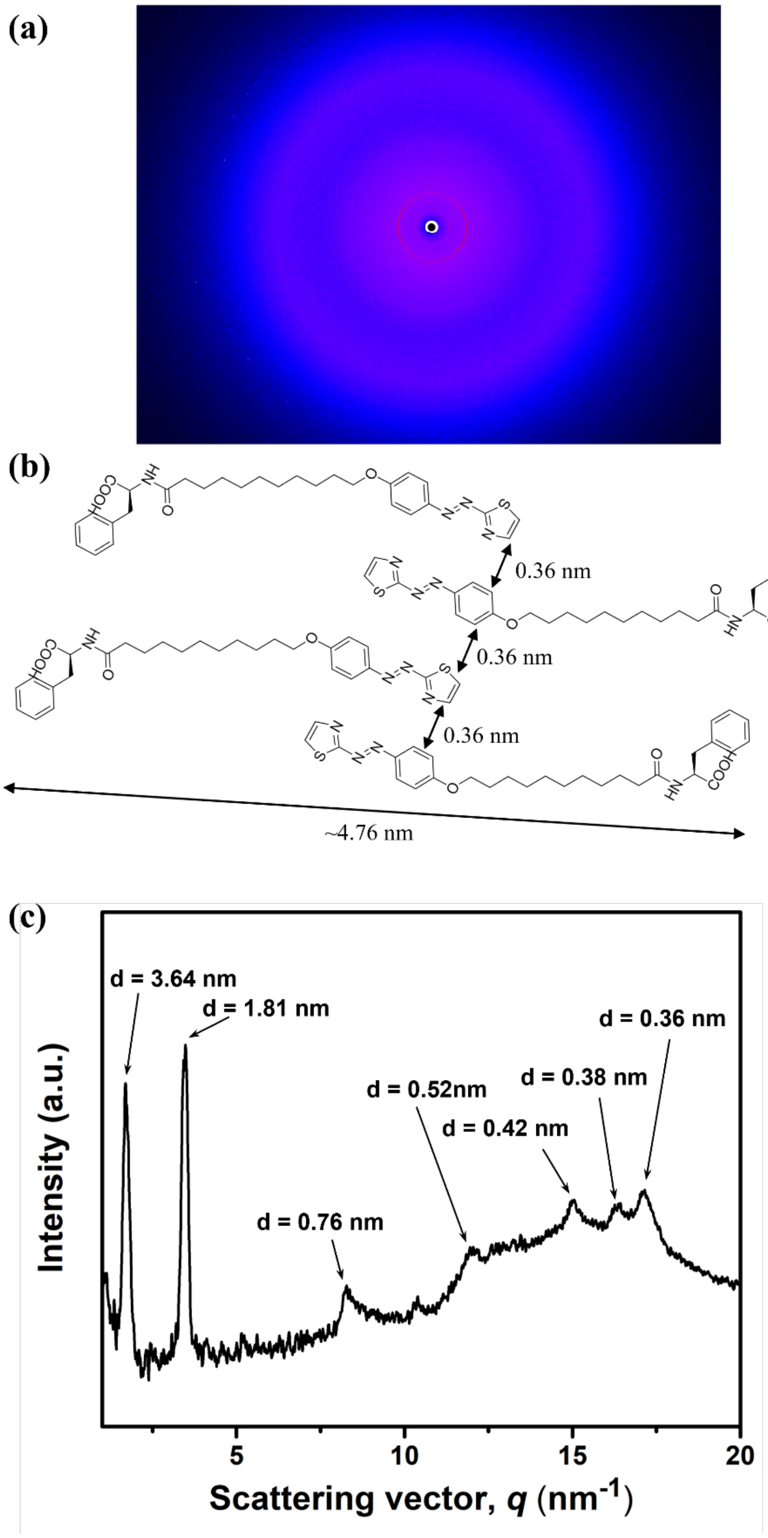

Figure S20. (a) 2D-WAXD image of a macroscopic soft scaffold of **PATA*L*** (5.0 wt.%, 93.2 mM). (b) Schematic illustration of assembled **PATA*L*** leads to diffraction in WAXD. The calculated molecular length of the assembled *E*-**PATA*L*** (~ 4.76 nm) is estimated by Chem3D with fully extended model, the actual length should be shorter. (c) 1D-WAXD pattern of a macroscopic soft scaffold of **PATA*L*** (5.0 wt.%, 93.2 mM), showing the diffraction pattern perpendicular to the long axis of the scaffold.

(a)

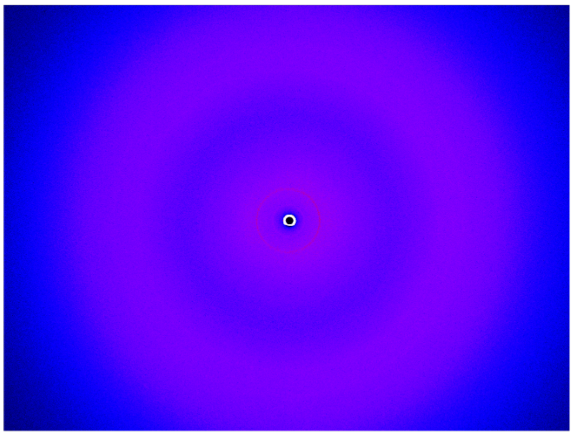

(b)

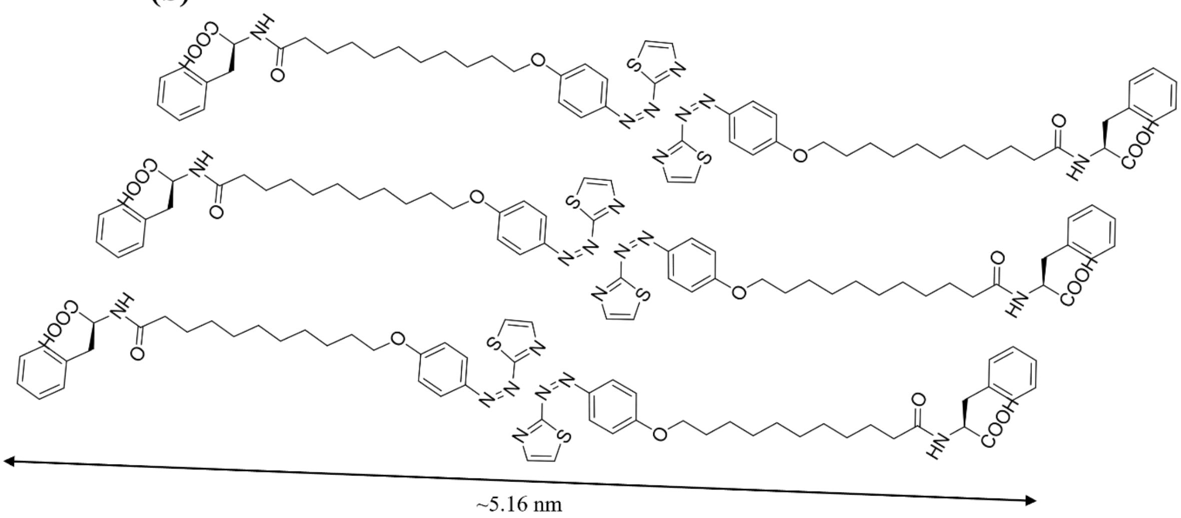

Figure S21. (a) 2D-WAXD image of a macroscopic soft scaffold of **PATA*L*** (5.0 wt.%, 93.2 mM) after irradiation with 430 nm blue-light. (b) Schematic illustration of assembled **PATA*L*** leads to diffraction in WAXD after irradiation with 430 nm blue-light. The calculated length of the assembled *Z*-**PATA*L*** (~5.16 nm) is estimated by Chem3D with fully extended model, the actual length should be shorter.

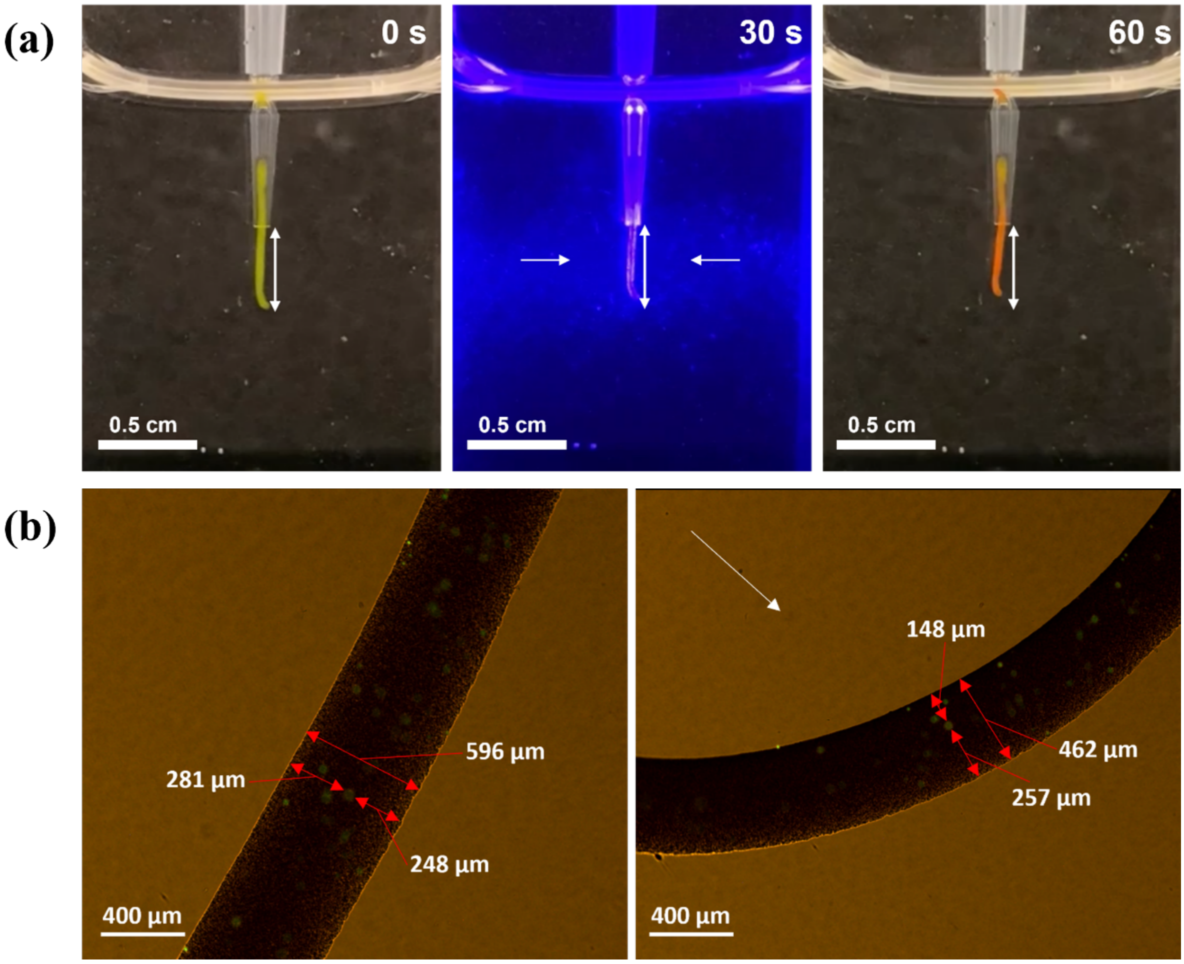

Figure S22. (a) Snapshots of macroscopic soft scaffold of **PATA*L*** (3.0 wt.%) in $CaCl_2$ (150 mM) before and after irradiation with two 430 nm blue-light sources from both sides. The length of macroscopic soft scaffold of **PATA*L*** is shortened after 60 s of irradiation. **(b)** Snapshots of a microbead (FluoSpheres™ Polystyrene Microspheres, Invitrogen™) encapsulated macroscopic soft scaffold of **PATA*L*** (3.0 wt.%) before and after irradiation with 430 nm blue-light source from left side for 120 s. The distances (red arrows) between a selected microbead and both sides of the scaffold were measured before and after irradiation. Direction of irradiation is indicated by white arrows. Scale bar: 400 μm.

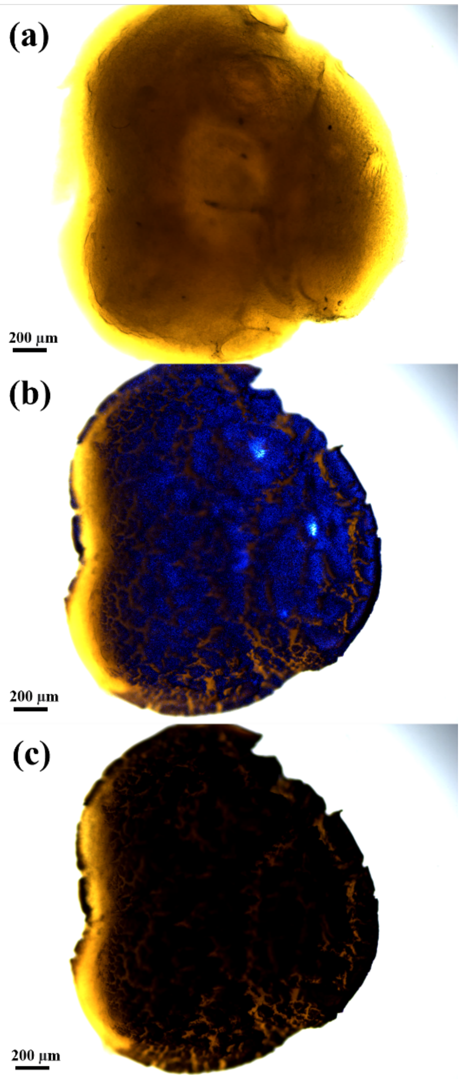

Figure S23. Snapshots of an isotropic soft scaffold of **PATA*L*** (3.0 wt.%) in 150 mM of $CaCl_2$ (a) before and after irradiation with 430 nm blue light source for (b) 30 s and (c) 60 s.

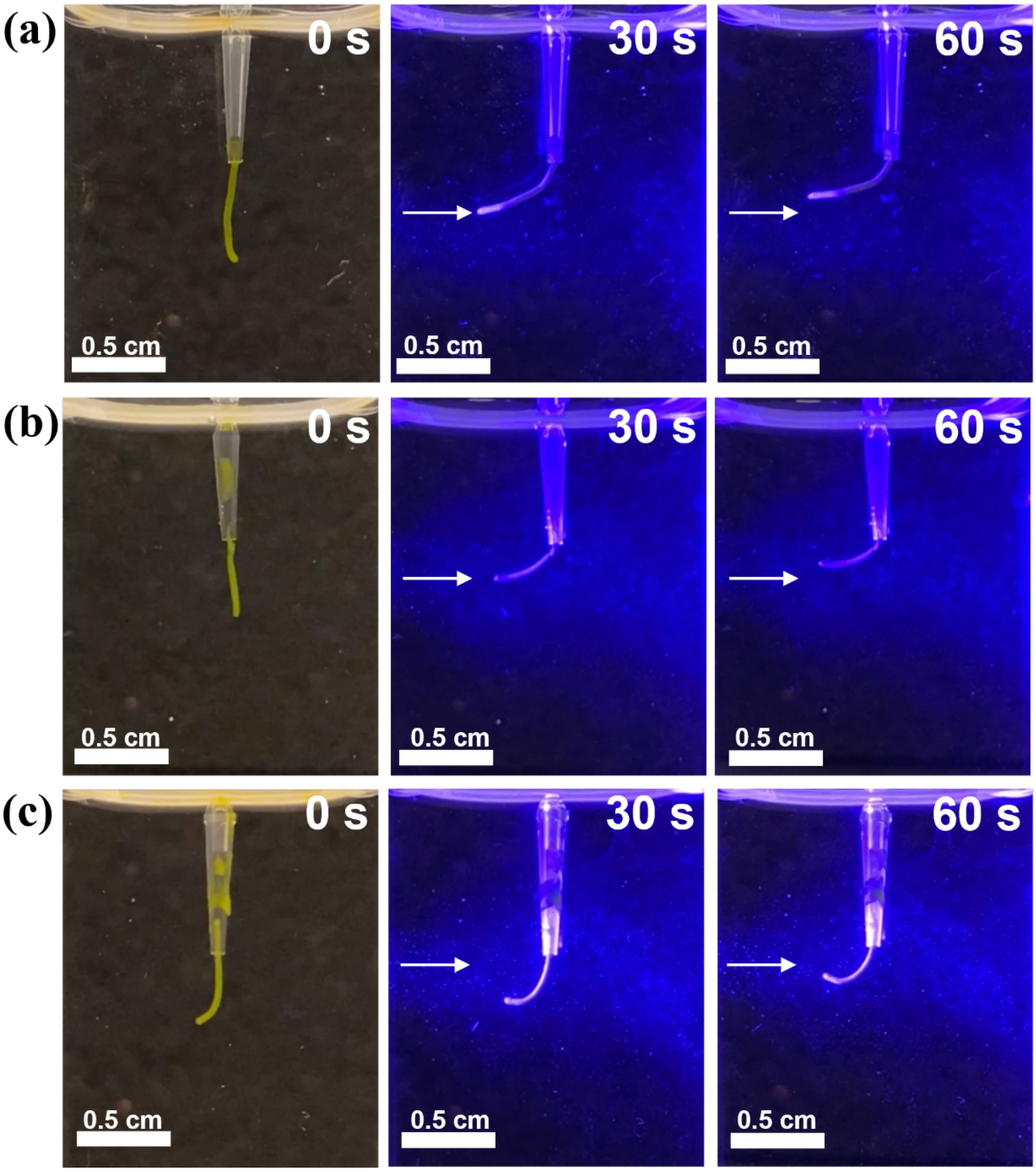

Figure S24. Snapshots of a macroscopic soft scaffold of **PATA*L*** (3.0 wt.%) in (a) 20 mM (actuation speed = 1.26 ± 0.13 °/s), (b) 50 mM (actuation speed = 1.29 ± 0.06 °/s) and (c) 100 mM of $CaCl_2$ (actuation speed = 1.17 ± 0.12 °/s) before and after irradiation with 430 nm blue-light source from left side. The macroscopic soft scaffold of **PATA*L*** bends towards the blue light source.

Concentration of $CaCl_2$	Young modulus (kPa)
20	7.75 ± 0.63
50	8.42 ± 1.11
150	8.44 ± 2.03

Table S1. The Young modulus of the macroscopic soft scaffold of **PATA*L*** (3.0 wt.%) prepared by different concentrations of calcium chloride.

Intensity (Lux)	Actuation Speed (°/s)
261	0.17 ± 0.03
638	0.55 ± 0.03
1060	0.86 ± 0.12
3190	1.07 ± 0.05
8020	1.39 ± 0.12

Table S2. The actuation speed of macroscopic soft scaffold of **PATA*L*** (3.0 wt.%) in 150 mM $CaCl_2$ against the intensity of Thorlabs M430F1 light sources. The distance between the macroscopic soft scaffold and light source is 1 cm. The intensities of irradiation light source were measured by a TASI model TA8121 digital light meter.

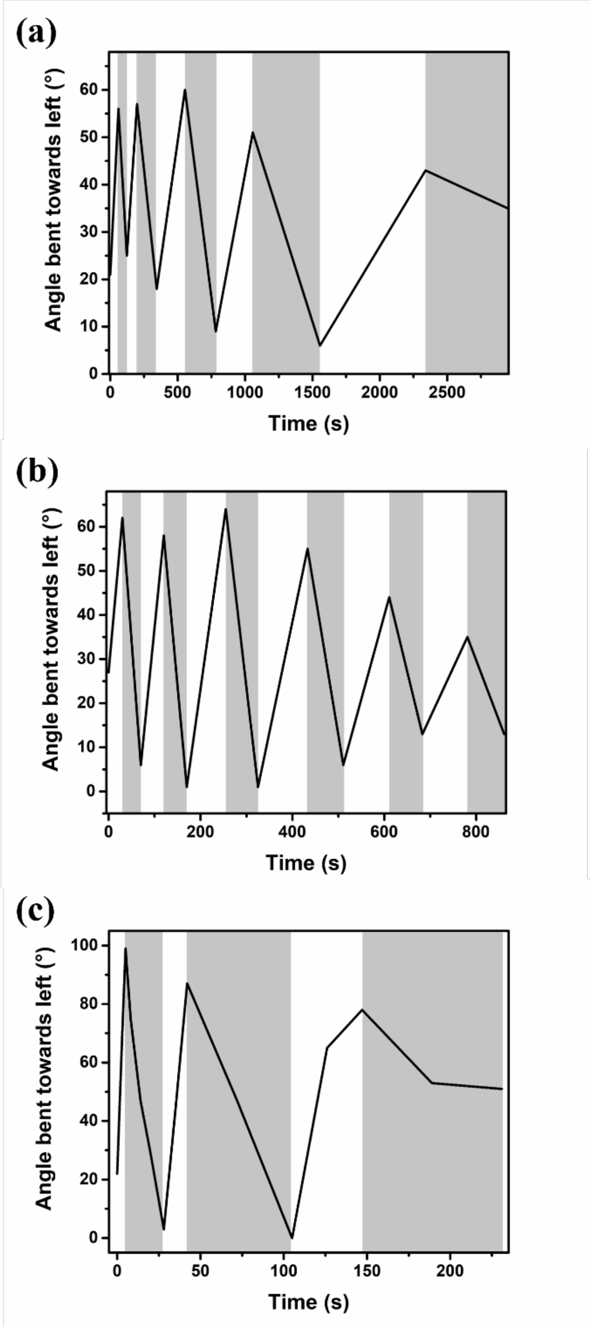

Figure S25. Graph of angle bent towards left as a function of irradiation time for macroscopic soft scaffold of **PATA*L*** with (a) 5.0 wt.%, (b) 3.0 wt.% and (c) 1.0 wt.% irradiated from left side (white region) and right side (grey region).

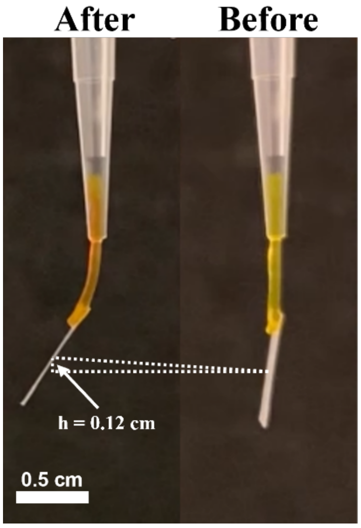

$W = mgh$

m, mass of the weight (center of the mass);

g, gravity acceleration;

h, distance of the weight that has been lifted vertically.

$W = 0.2\ \text{mg} \times 9.81\ (\text{m/s}^2) \times 0.12\ (\text{cm})$

$W = 0.2 \times 10^{-6}\ (\text{kg}) \times 9.81\ (\text{m/s}^2) \times 0.12 \times 10^{-2}\ (\text{m})$

$W = 2.35\ \text{nJ}$

Figure S26. Calculation of the work over the weightlifting process.

(a)
(b)
(c)

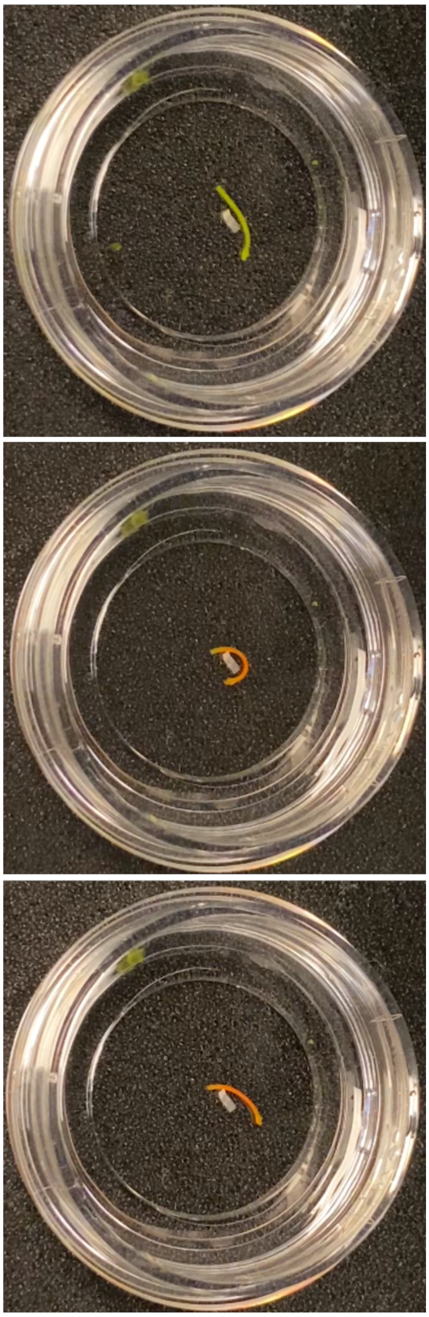

Figure S27. Snapshots of a macroscopic soft scaffold of **PATA*L*** (3.0 wt.%) in $CaCl_2$ (150 mM) (a) before, (b) changed to a curved-shape upon photoirradiation to grasp a paper and (c) changed to a linear-shape upon photoirradiation on opposite side to release the paper.

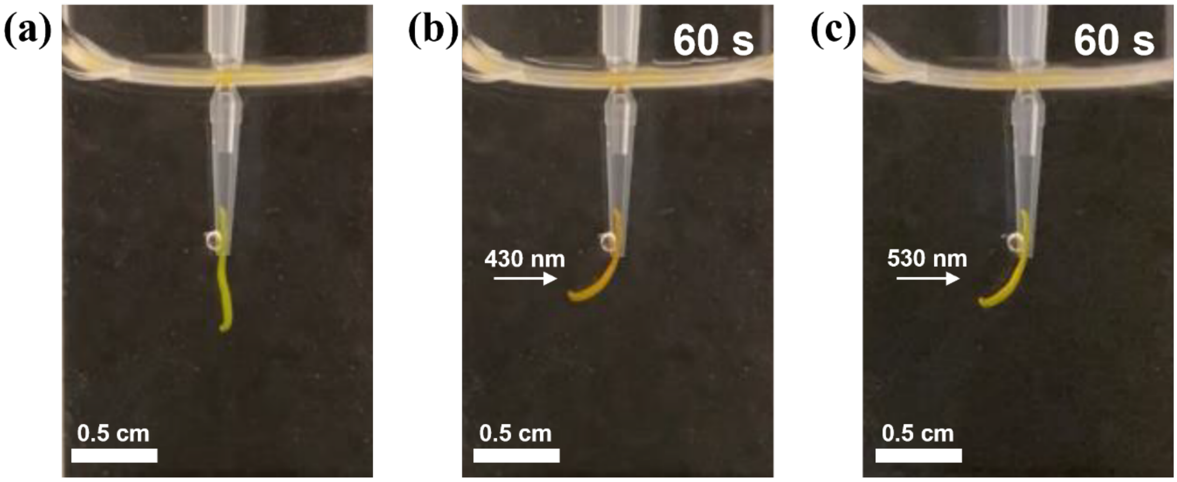

Figure S28. Snapshots of a macroscopic soft scaffold of **PATA$_L$** (3.0 wt.%) in $CaCl_2$ (150 mM) (a) before irradiation, (b) after irradiation with 430 nm blue-light source from left side and (c) sequent irradiation with 530 nm green-light source from left side.

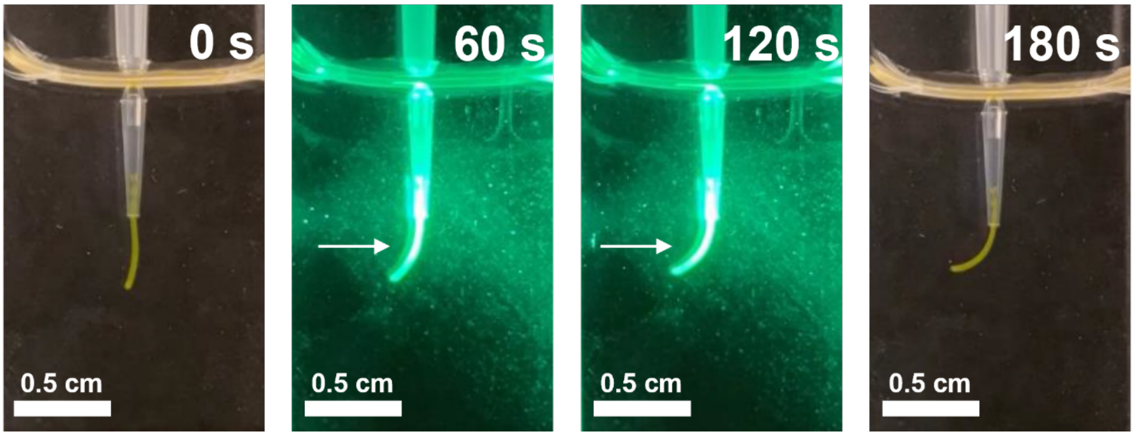

Figure S29. Snapshots of a macroscopic soft scaffold of **PATA**$_L$ (3.0 wt.%) in $CaCl_2$ (150 mM) before and after irradiation with 530 nm green-light source from left side for 180 s. The macroscopic soft scaffold of **PATA**$_L$ bends towards the green light, possibly due to broad-excitation of the green-light source.

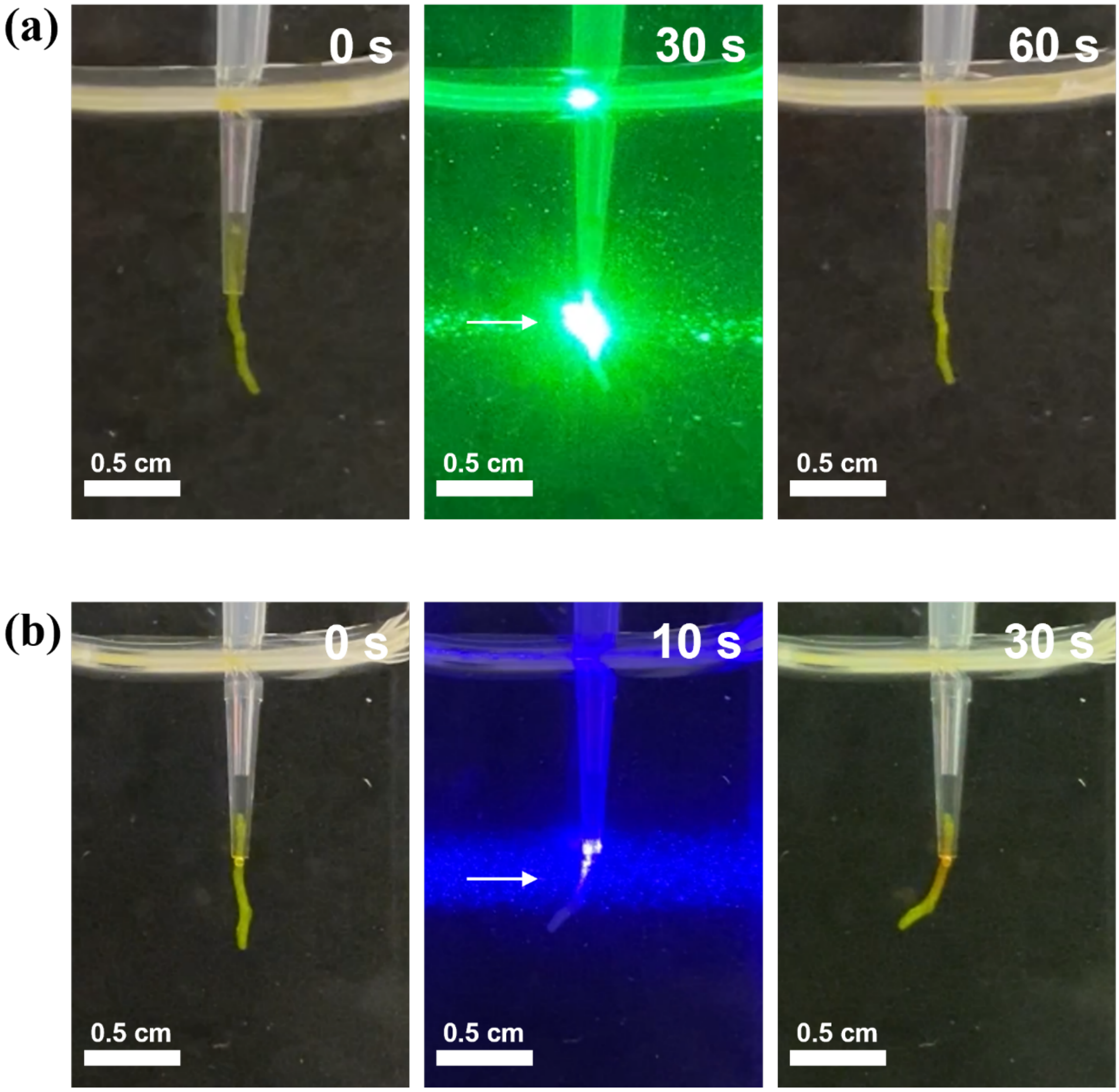

Figure S30. Snapshots of a macroscopic soft scaffold of **PATA$_L$** (3.0 wt.%) in $CaCl_2$ (150 mM) before and after irradiation with (a) green-light laser and (b) blue-light laser from left side for 60 s and 30 s, respectively.

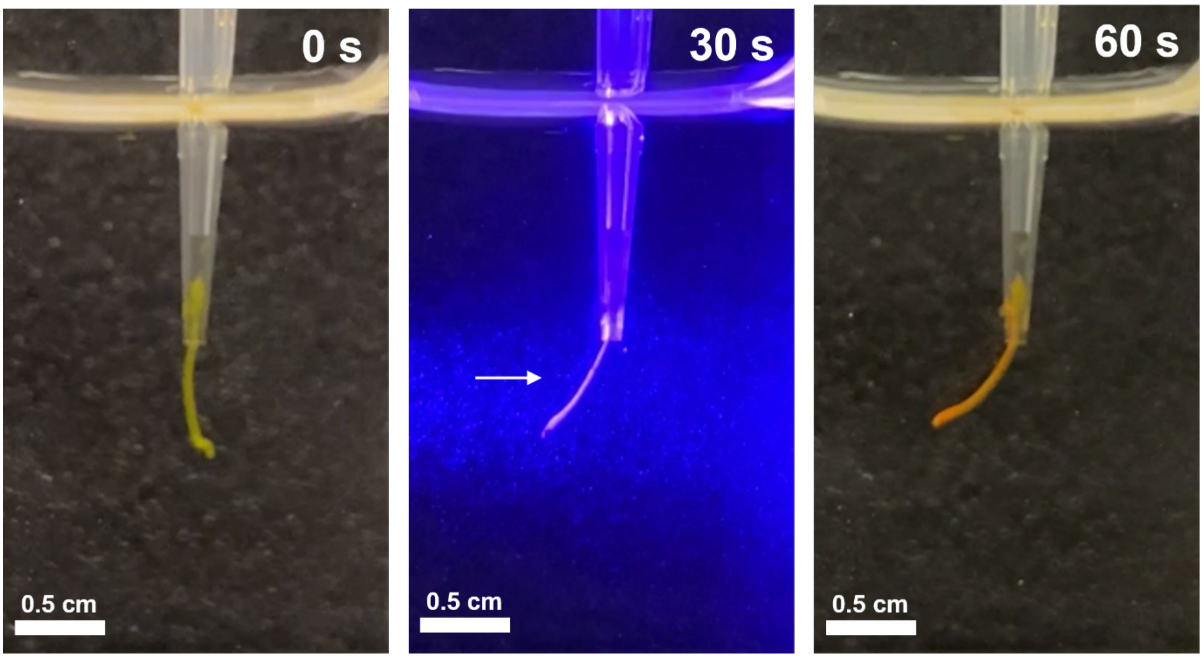

Figure S31. Snapshots of a macroscopic soft scaffold of **PATA*D*** (3.0 wt.%) in $CaCl_2$ (150 mM) before and after irradiation with 430 nm blue-light source from left side for 60 s. The macroscopic soft scaffold of **PATA*D*** bends towards the blue light.

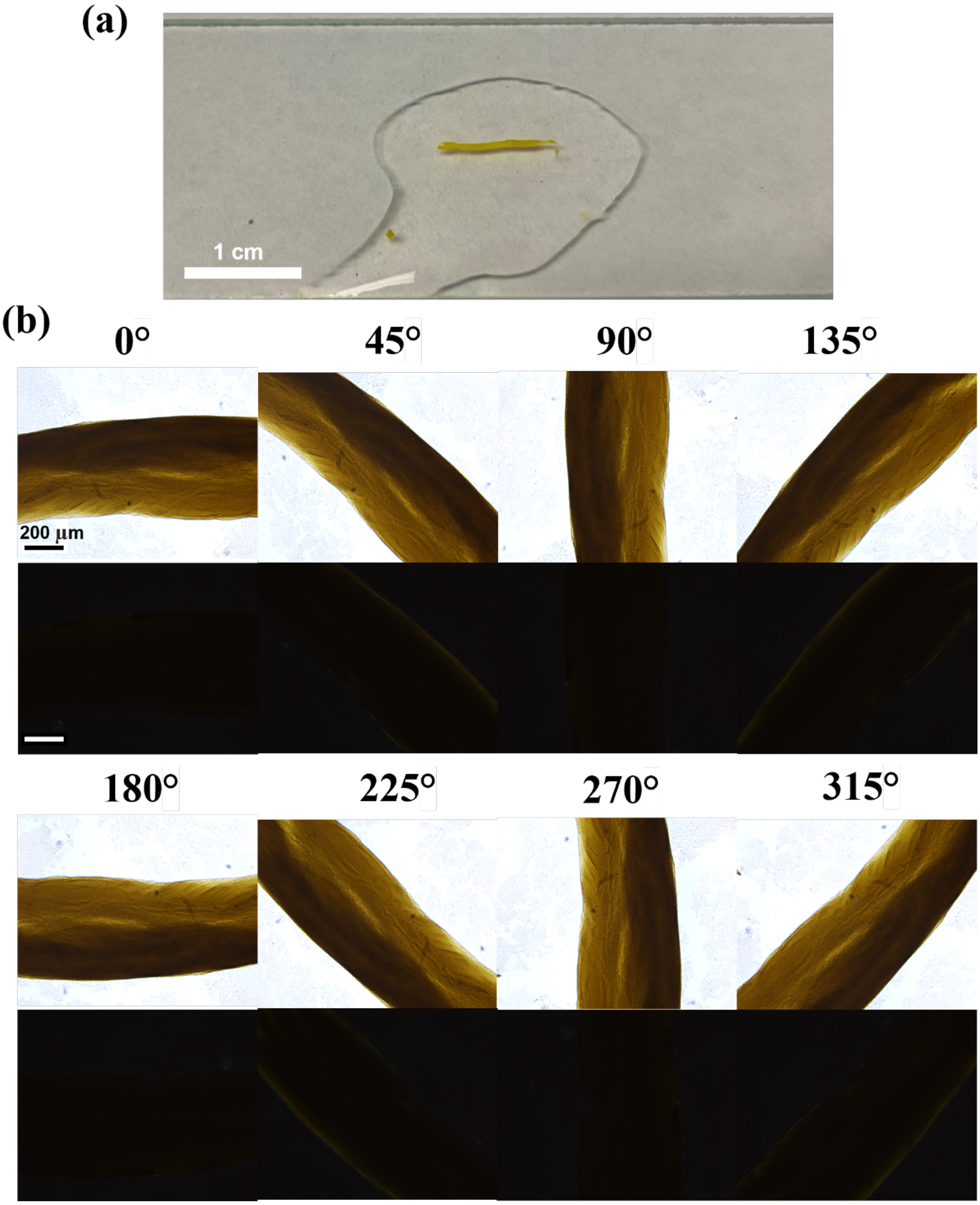

Figure S32. (a) Photograph of a macroscopic soft scaffold of **PATA*L+D*** (5.0 wt.%, 1:1) prepared from an aqueous solution of calcium chloride (150 mM). (b) Optical microscopic images of a macroscopic soft scaffold of **PATA*L+D*** (5.0 wt.%, 1:1) prepared from a solution of $CaCl_2$ (150 mM) under crossed polarizers. The POM and OM images of the soft scaffold were tilted at 0°, 45°, 90°, 135°, 180°, 225°, 270° and 315° relative to the transmission axis of the analyzer. Scale bar 200 μm for all panels.

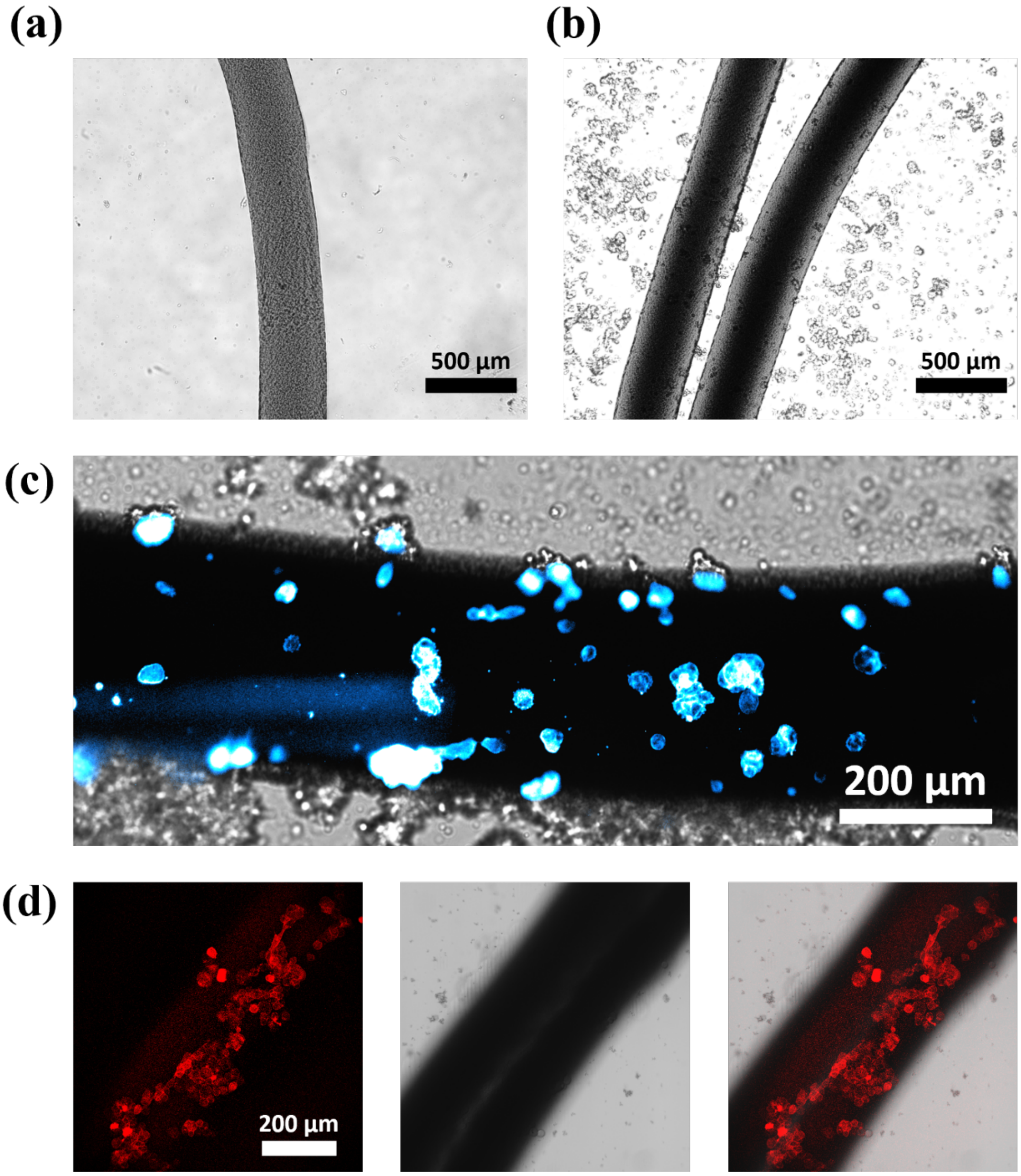

Figure S33. (a) Bright field microscopy representing the formation of macroscopic scaffold of **PATA**$_L$ (3.0 wt.%) in αMEM medium supplemented with 10% Fetal bovine serum and 1% Antibiotic-Antimycotic, scalebar: 500 μm. (b) Bright field microscopy representing cell seeding of human mesenchymal stem cells (hMSCs) with macroscopic scaffold of **PATA**$_L$ (3.0 wt.%), scalebar: 500 μm. (c) Microscopic image representing the SPY555 Fast Act labelled hMSCs (cyanhot) on the **PATA**$_L$ macroscopic scaffold surface after 5h culture using bioinert cell culture dish, scalebar: 200µm. (d) Microscopic images representing the CellMask DeepRed Actin labelled C2C12myoblasts on macroscopic scaffold of **PATA**$_L$ (3.0 wt.%) after 4 h culture using bio-inert cell culture dish. Left: C2C12myoblasts (CellMask DeepRed Actin); Middle: Brightfield image showing the macroscopic scaffold; Right: Merged image, scale bar:200 μm.

Analytical Data

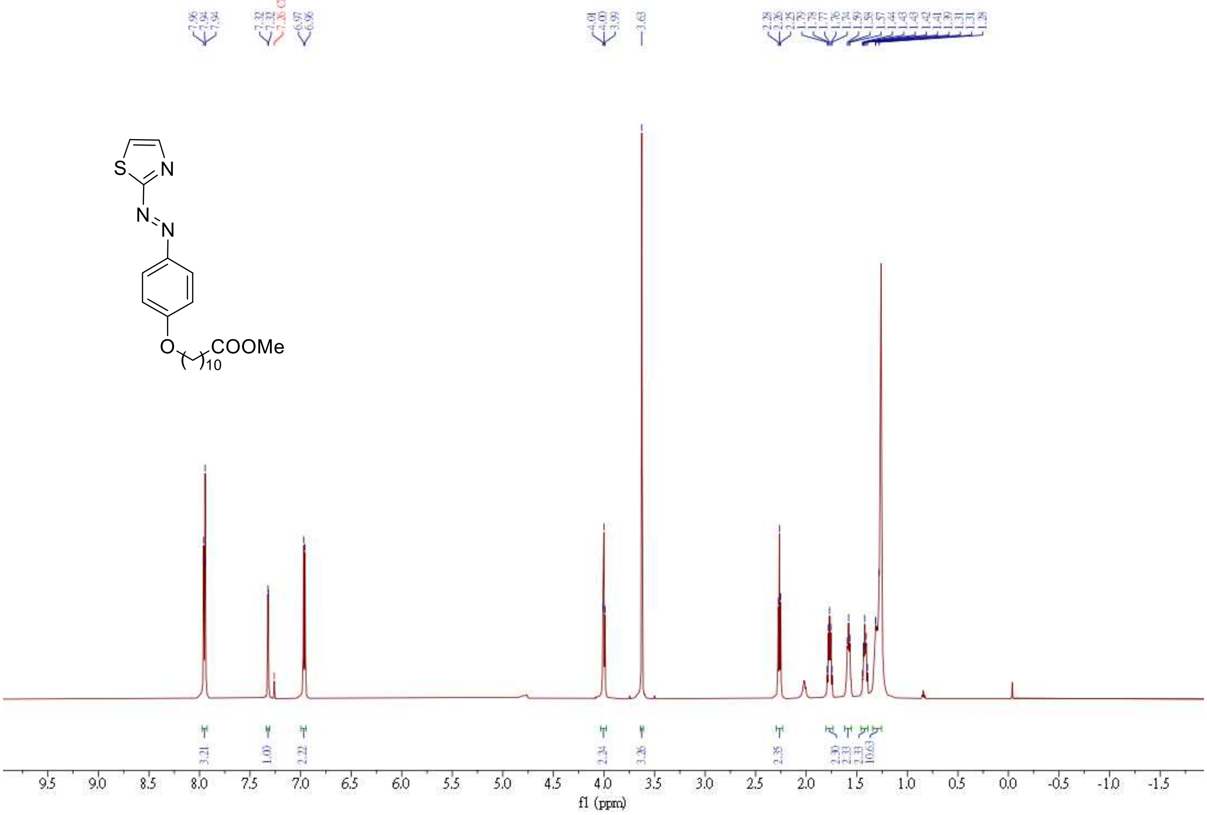

Figure S34. ^{1}H NMR spectrum (600 MHz) of compound **2** in $CDCl_3$ at 25 °C

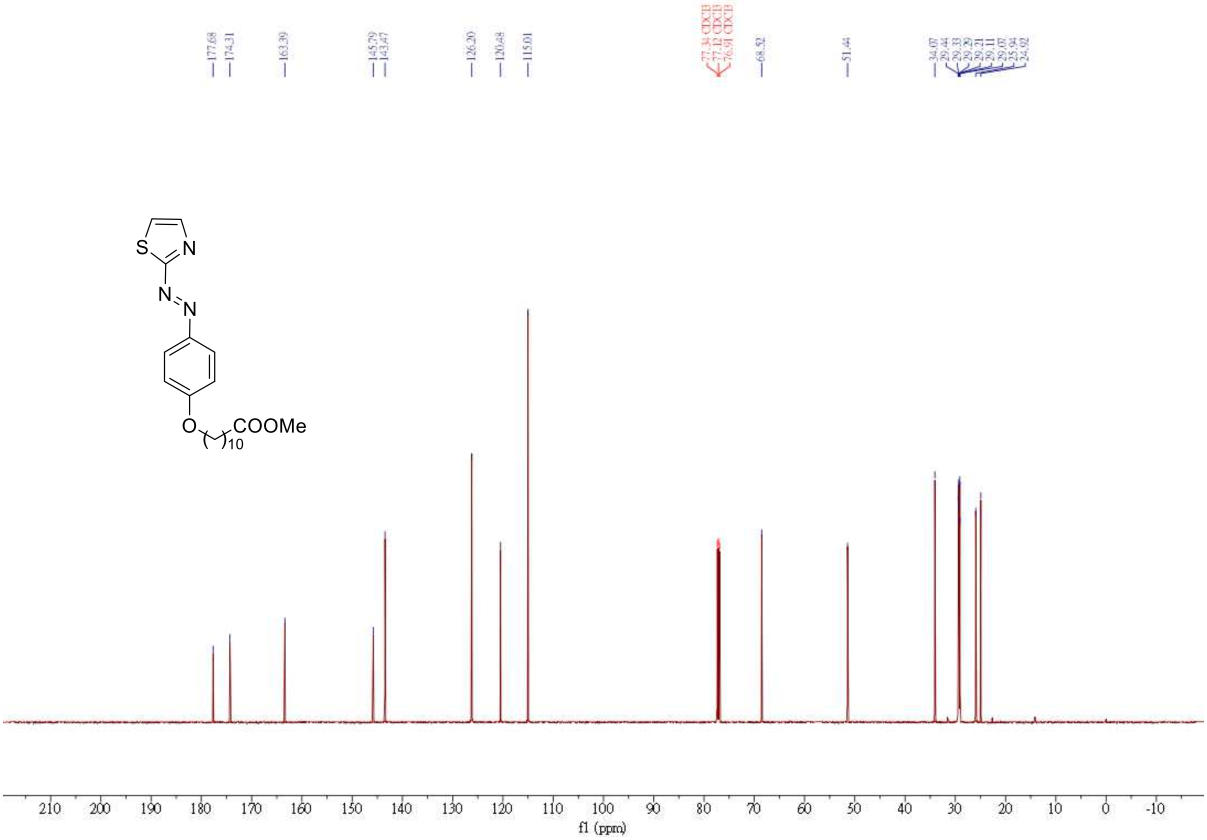

Figure S35. ^{13}C NMR spectrum (151MHz) of compound **2** in $CDCl_3$ at 25 °C.

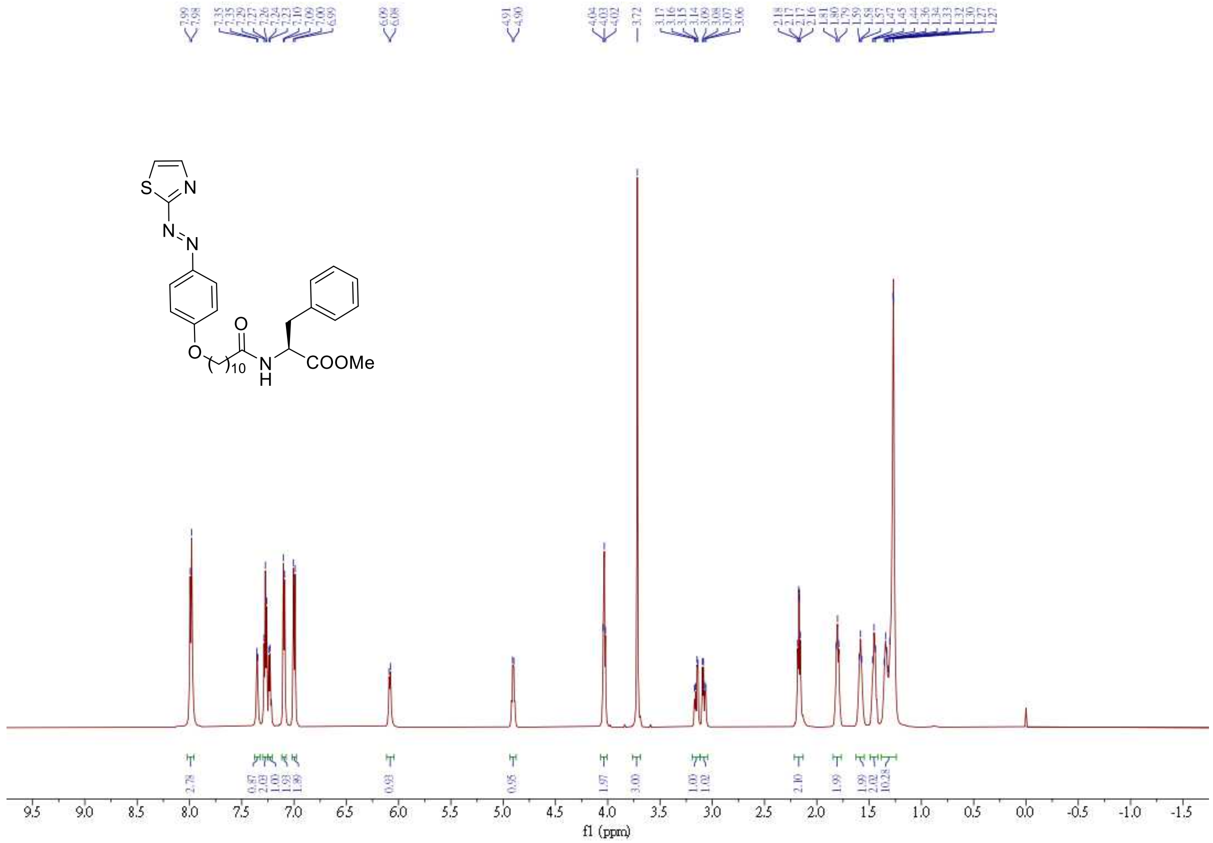

Figure S36. ^{1}H NMR spectrum (600 MHz) of compound **4** in $CDCl_3$ at 25 °C

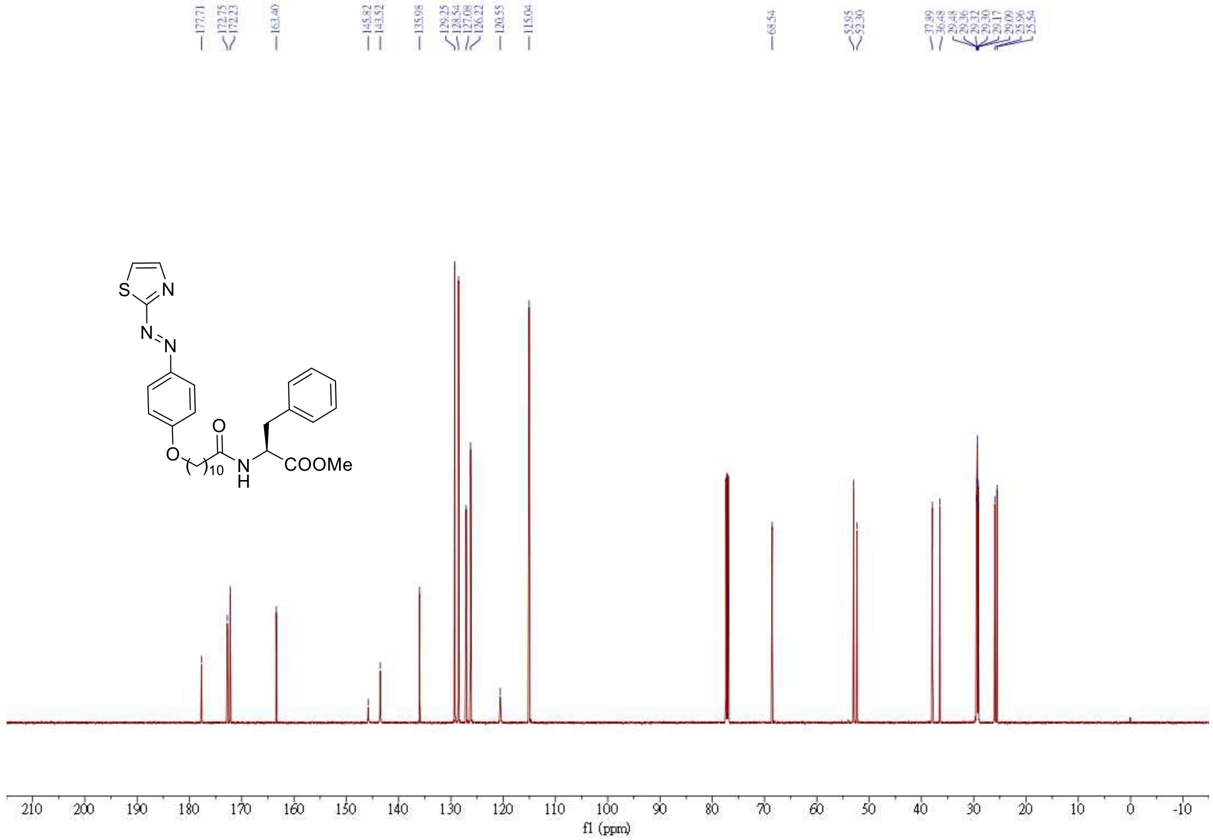

Figure S37. ^{13}C NMR spectrum (151MHz) of compound **4** in $CDCl_3$ at 25 °C.

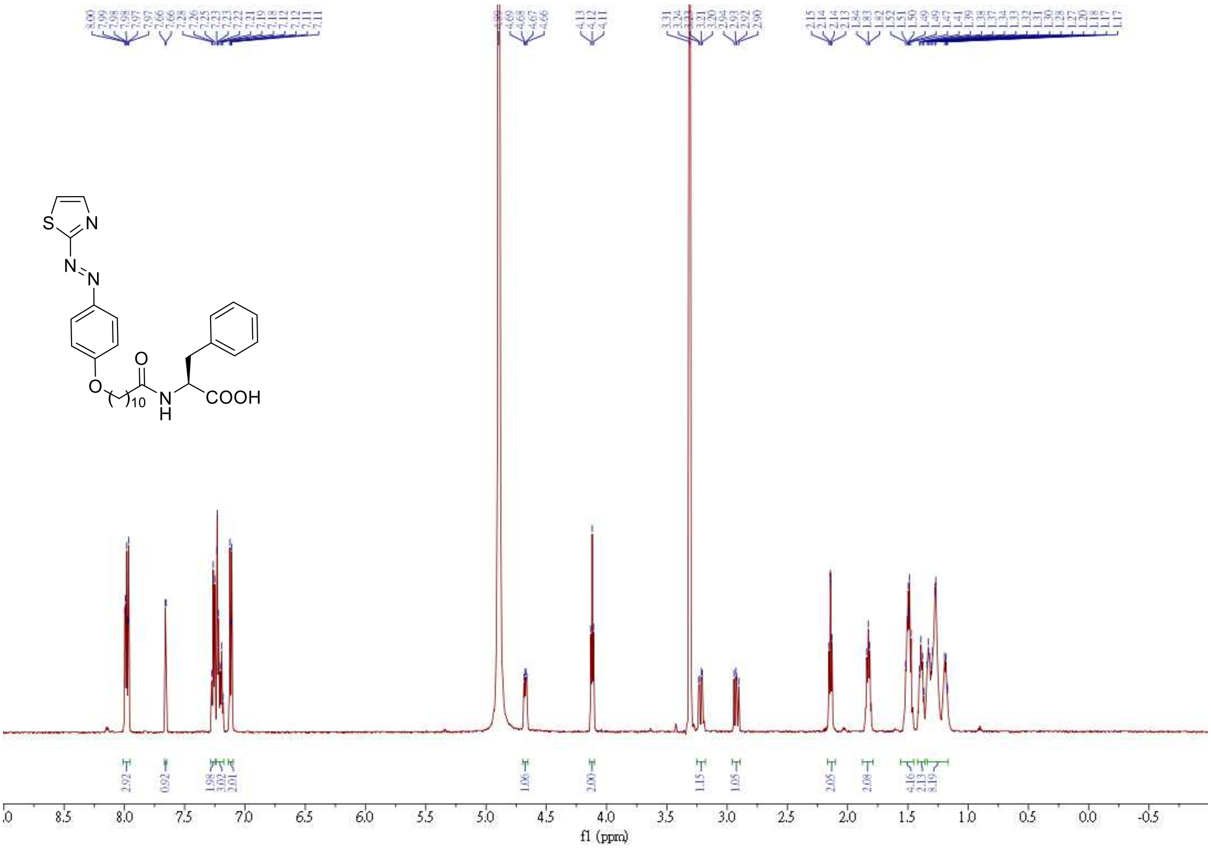

Figure S38. ^{1}H NMR spectrum (400 MHz) of **PATA*L*** in MeOD at 25 °C.

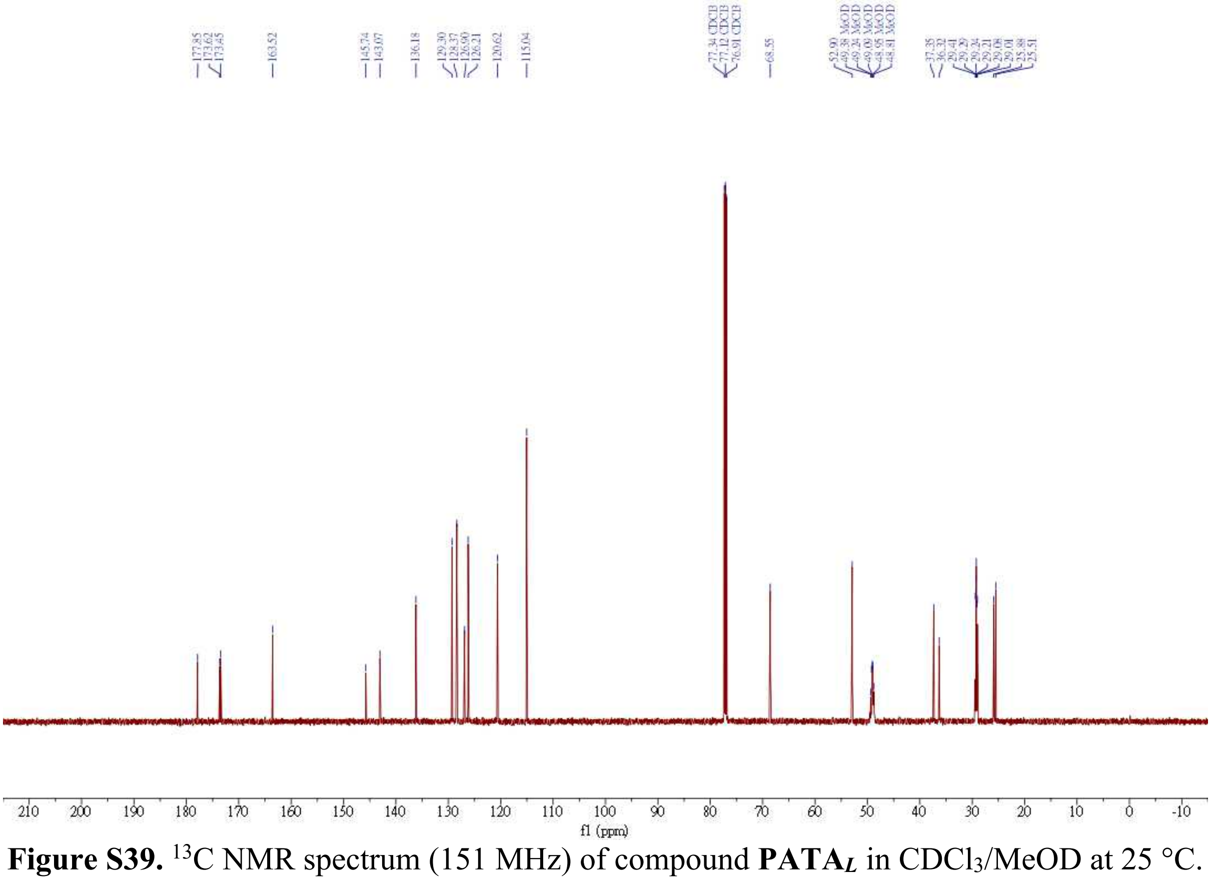

Figure S39. ^{13}C NMR spectrum (151 MHz) of compound **PATA*L*** in $CDCl_3$/MeOD at 25 °C.

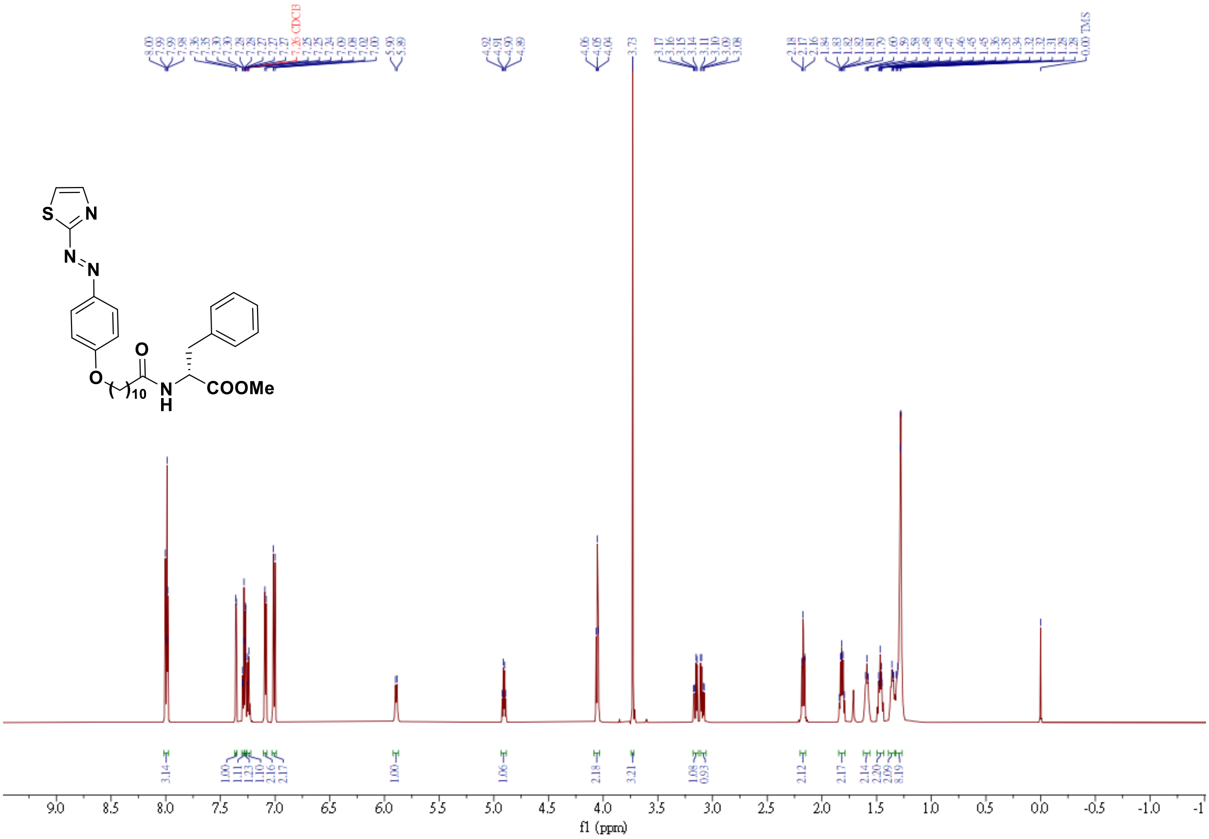

Figure S40. ^{1}H NMR spectrum (600 MHz) of compound **5** in $CDCl_3$ at 25 °C.

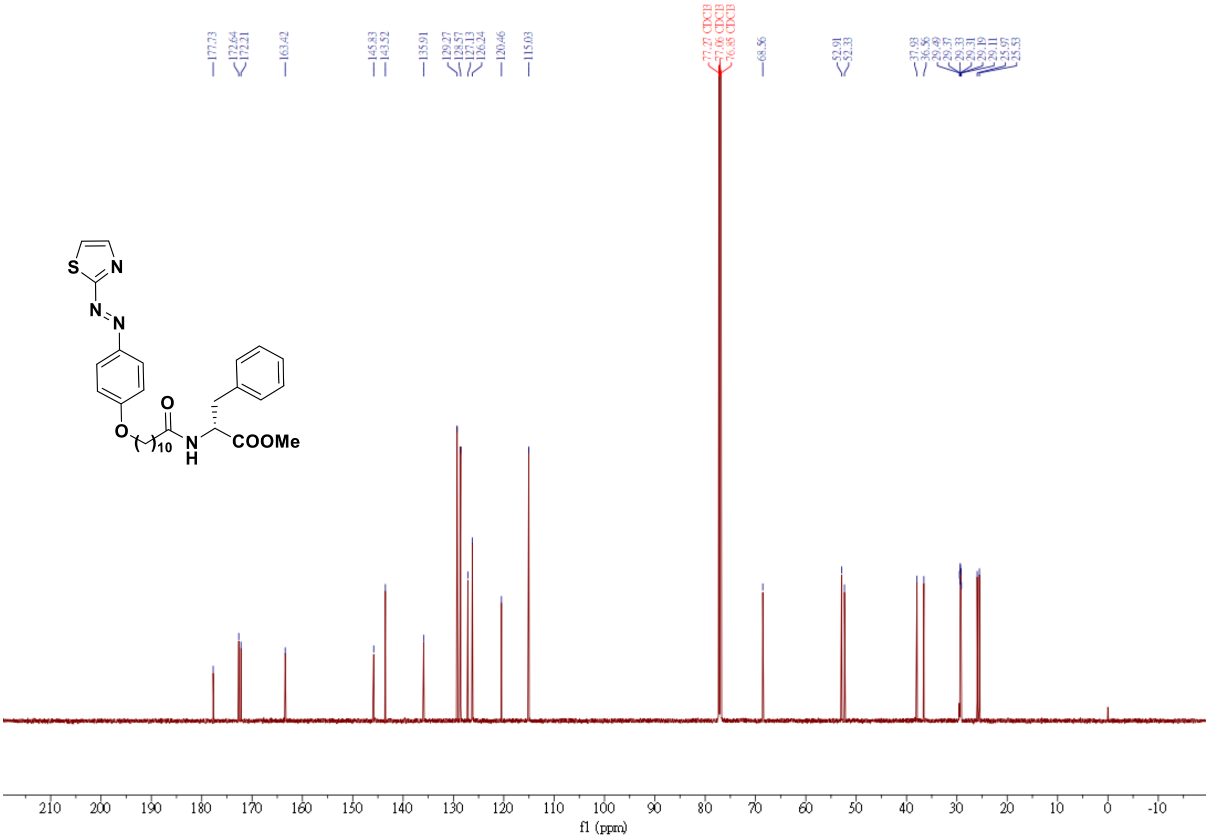

Figure S41. ^{13}C NMR spectrum (151 MHz) of compound **5** in $CDCl_3$ at 25 °C.

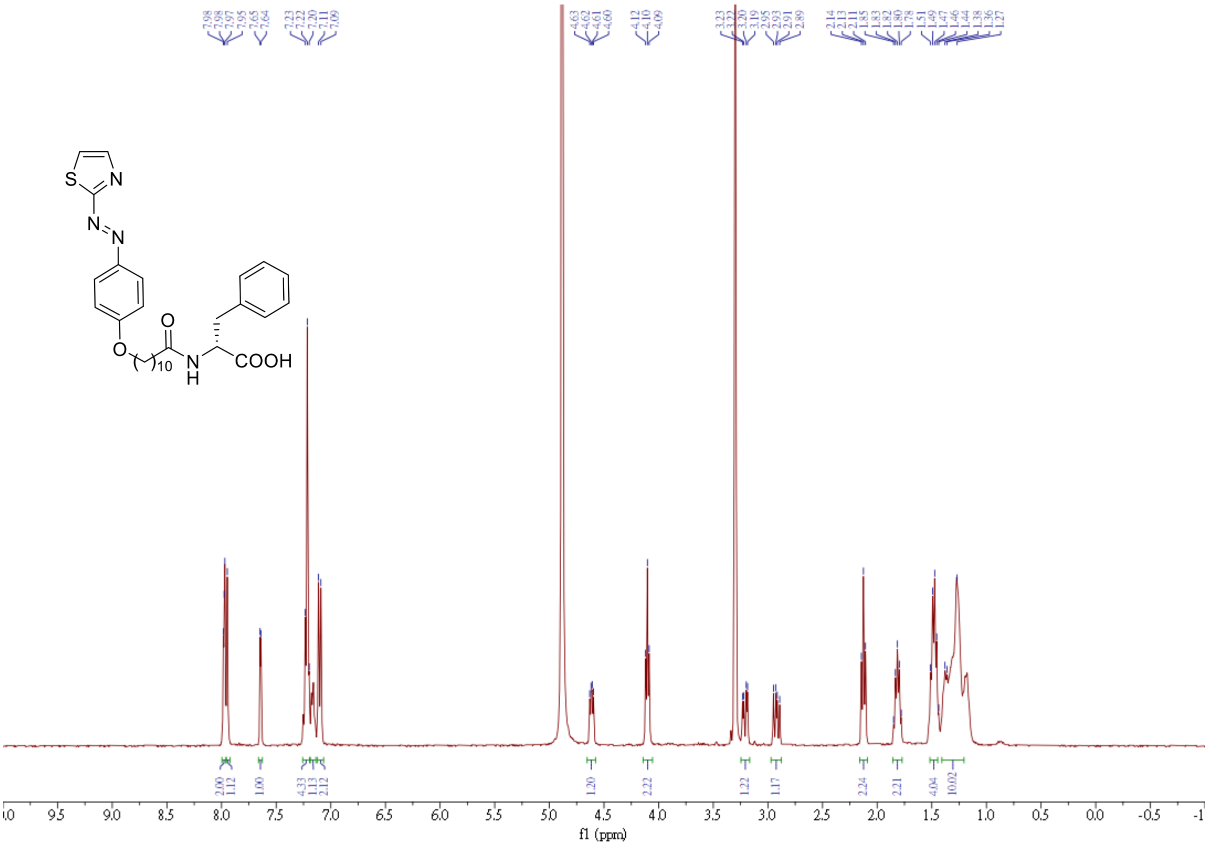

Figure S42. ^{1}H NMR spectrum (600 MHz) of **PATA*D*** in $CDCl_3$ at 25 °C.

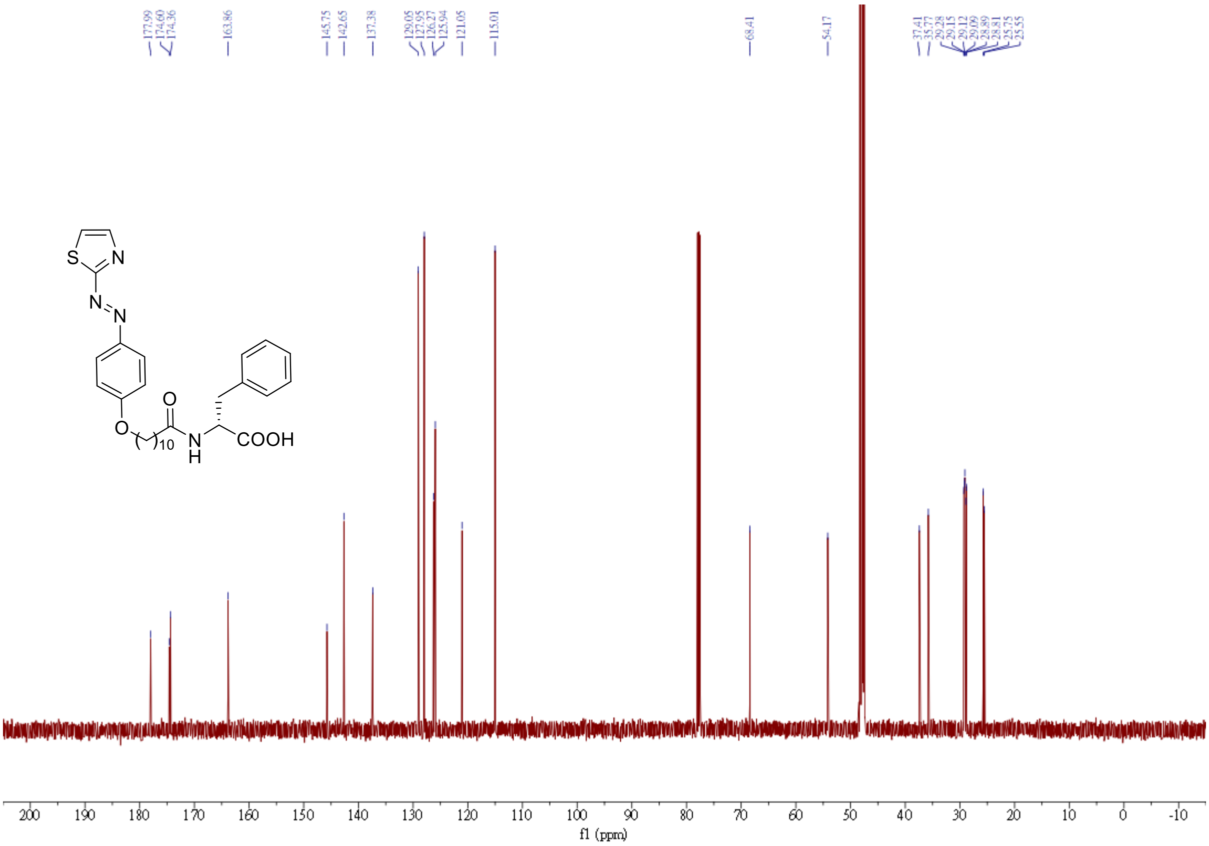

Figure S43. ^{13}C NMR spectrum (151 MHz) of compound **PATA*D*** in $CDCl_3$/MeOD at 25 °C.

References

(1) Tantakitti, F.; Boekhoven, J.; Wang, X.; Kazantsev, R. V.; Yu, T.; Li, J.; Zhuang, E.; Zandi, R.; Ortony, J. H.; Newcomb, C. J.; Palmer, L. C.; Shekhawat, G. S.; de la Cruz, M. O.; Schatz, G. C.; Stupp, S. I. Energy Landscapes and Functions of Supramolecular Systems. *Nat. Mater.* **2016**, *15* (4), 469–476.

(2) Lin, R.; Hashim, P. K.; Sahu, S.; Amrutha, A. S.; Cheruthu, N. M.; Thazhathethil, S.; Takahashi, K.; Nakamura, T.; Kikukawa, T.; Tamaoki, N. Phenylazothiazoles as Visible-Light Photoswitches. *J. Am. Chem. Soc.* **2023**, *145* (16), 9072–9080.
